# FT-GPI, a highly sensitive and accurate predictor of GPI-anchored proteins, reveals the composition and evolution of the GPI proteome in *Plasmodium* species

**DOI:** 10.1186/s12936-022-04430-0

**Published:** 2023-01-25

**Authors:** Lena M. Sauer, Rodrigo Canovas, Daniel Roche, Hosam Shams-Eldin, Patrice Ravel, Jacques Colinge, Ralph T. Schwarz, Choukri Ben Mamoun, Eric Rivals, Emmanuel Cornillot

**Affiliations:** 1 Institute for Virology, Hans-Meerwein-Straße, 35043 Marburg, Germany; 2 Computational Biology Institute, Campus Saint Priest, 161 Rue Ada, 34095 Montpellier, France; 3 LIRMM, CNRS, Université de Montpellier, Campus Saint Priest, 161 Rue Ada, 34095 Montpellier, France; 4 Institut de Recherche en Cancérologie de Montpellier INSERM U1094, ICM, Université de Montpellier, Campus Val d’Aurelle, 208 Avenue Des Apothicaires, 34298 Montpellier, France; 5 Department of Internal Medicine, Section of Infectious Diseases, Yale School of Medicine, New Haven, CT 06520 USA; 6 Institut Français de Bioinformatique, CNRS UAR 3601, 2, rue Gaston Crémieux, 91057 Évry, France; 7 Wespran SAS, 13 Rue de Penthièvre, 75008 Paris, France; 8 Present Address: GRN-Klinik Sinsheim, Alte Waibstadter Straße 2a, 74889 Sinsheim, Germany

**Keywords:** *Plasmodium falciparum*, *P. vivax*, GPI-proteome, GPI-anchored protein, FT-GPI

## Abstract

**Background:**

Protozoan parasites are known to attach specific and diverse group of proteins to their plasma membrane via a GPI anchor. In malaria parasites, GPI-anchored proteins (GPI-APs) have been shown to play an important role in host–pathogen interactions and a key function in host cell invasion and immune evasion. Because of their immunogenic properties, some of these proteins have been considered as malaria vaccine candidates. However, identification of all possible GPI-APs encoded by these parasites remains challenging due to their sequence diversity and limitations of the tools used for their characterization.

**Methods:**

The FT-GPI software was developed to detect GPI-APs based on the presence of a hydrophobic helix at both ends of the premature peptide. FT-GPI was implemented in C ++and applied to study the GPI-proteome of 46 isolates of the order Haemosporida. Using the GPI proteome of *Plasmodium falciparum* strain 3D7 and *Plasmodium vivax* strain Sal-1, a heuristic method was defined to select the most sensitive and specific FT-GPI software parameters.

**Results:**

FT-GPI enabled revision of the GPI-proteome of *P. falciparum* and *P. vivax,* including the identification of novel GPI-APs. Orthology- and synteny-based analyses showed that 19 of the 37 GPI-APs found in the order Haemosporida are conserved among *Plasmodium* species. Our analyses suggest that gene duplication and deletion events may have contributed significantly to the evolution of the GPI proteome, and its composition correlates with speciation.

**Conclusion:**

FT-GPI-based prediction is a useful tool for mining GPI-APs and gaining further insights into their evolution and sequence diversity. This resource may also help identify new protein candidates for the development of vaccines for malaria and other parasitic diseases.

**Supplementary Information:**

The online version contains supplementary material available at 10.1186/s12936-022-04430-0.

## Background

Malaria, a parasitic disease caused by intraerythrocytic parasites of the genus *Plasmodium*, remains one of the deadliest infectious diseases affecting humans. In 2019, the World Health Organization (WHO) recorded 227 million cases, and this figure rose to 241 million in 2020 due to major disruptions to health infrastructures during the COVID-19 epidemic [1]. Africa accounted for the vast majority of cases with 99.7% of infections caused by *Plasmodium falciparum*. About 500,000 malaria cases were fatal, with children under the age of five accounting for two-thirds of deaths. Thanks to comprehensive preventative measures and improved access to artemisinin-based combination therapy, morbidity and mortality showed a downward trend until 2019. However, a partial artemisinin resistance which originally emerged in 2008 at the border between Cambodia and Thailand [2] and subsequently spread throughout Southeast Asia [3–5] could undermine efforts to control the global impact of the disease including in Africa [6, 7]. The identification of new targets, therefore, remains essential for the development of new anti-malarial medication.

The functions of the Glycosylphosphatidylinositol (GPI) moieties and GPI-Anchored proteins (GPI-Aps) are diverse ranging from protective properties to mediating complex endocytosis mechanisms in the cell [8, 9]. While GPI-APs could take on specialized tasks, especially in higher eukaryotes [10], some of them are expressed at very high levels and are predominant on the membranes of parasites including *Plasmodium spp* [11, 12]. GPI-APs have also been found in archaebacteria [13] and plants [14], as well as in association with cancer antigens, herpes viruses and prion proteins [9, 15]. According to current research, about 1–2% of all proteins encoded in the eukaryotic genome or 10–20% of all membrane proteins that transit the secretory pathway are bound in the ER to a GPI anchor [14, 16–18]. In eukaryotic cells, GPI-APs are translated by endoplasmic reticulum (ER)-associated ribosomes and delivered through the secretory pathway to their final destination [19]. The ER-resident GPI transamidase catalyzes the fusion of the target protein to the GPI at the site of anchor addition (site).

Each GPI molecule has a core structure, conserved across species, consisting of myo-inositol, ethanolamine, mannose and a non-acylated glucosamine (GlcN), which is combined with a lipid moiety to form ethanolamine-6-Mannose(α1-2)-Mannose(α1-6)-Mannose(α1-4)-glucosamine(1-6)-myo-inositol-phosphate-lipid [16, 19]. So far, *Babesia* species are the only organisms that lack the conserved core glycan [20, 21]; in its place is a 2(Man)-GlcN resulting from the loss of the PigB gene [22]. The hydrophobic residue consists of diacyl-glycerol, alkyl/acyl-glycerol, monoacyl-glycerol and/or ceramides [23, 24]. Free and bound GPIs are abundant glycolipids in the membranes of protozoan parasites and may function as toxins due to their pro-inflammatory properties in the mammalian host [20, 25]. These proteins can induce apoptosis in the heart, spleen and liver of mice and in this respect are also suspected of being responsible for the myocardial functional impairments in patients with severe episodes of malaria [26]. Chemically synthetized *P. falciparum* GPI were found to be protective against severe symptoms of malaria in mice [27].

Due to their ubiquitous occurrence and their manifold significant functions, GPI-anchored proteins represent an important and worthwhile object of research. Successful recombinant vaccines based on these proteins have been developed against apicomplexan parasites. Vaccines against bovine and dog babesiosis provide protective immunity against the causative Babesia agents in animals [28, 29]. GlaxoSmithKline successfully developed the RTS,S malaria vaccine based on the GPI-anchored circumsporozoite protein (CSP) [30]. The GPI-proteome of the human malaria parasite *P. falciparum* was first described by Gilson and colleagues in the 3D7 strain [17]. The reported proteome consisted of 30 GPI-APs, several of which have been well characterized. The merozoite surface proteins multigene (MSP) family encodes several such members, including MSP1, 2, 4, 5, 8 and 10. MSP1 is a major hub protein interacting with non-GPI MSPs (MSP3, 6, 7 and 9) to form a merozoite surface complex involved in host attachment during invasion [31, 32]. Both the GPI-APs MSP1 and rhoptry-associated membrane antigen RAMA bind to specific rhoptry proteins once the rhoptries discharge their contents during invasion [33]. MSP2 and 4 are merozoite surface proteins that remain unprocessed during parasite invasion [34, 35]. In addition, eight proteins of the 6-cysteine protein family, which also play an important role in host-parasite interaction, are part of the GPI proteome of this parasite [36, 37]. To date, the GPI-proteome of only a few species has been elucidated [38, 39]. Furthermore, despite extensive research efforts, the exact functions of several GPI-APs and their roles in mediating pathogen-host interactions remain unknown. Whether the evolution of these proteins could have contributed to the speciation of *Plasmodium* spp needs to be further examined [40].

Structural patterns common to GPI-AP proteins enable computer algorithms to identify GPI-AP candidates by analyzing protein primary structure. Several algorithms have been developed using well-characterized GPI proteins as a training set. Big-PI [41, 42] and DGPI [43] were designed to detect specific amino acids surrounding the (ω) site, with Big-PI specifically trained to detect GPI-Aps using plant proteins. Later, three software programs were developed that use machine learning based on neural network and/or Hidden Markov Model (HMM). GPI-SOM operates on the basis of a Kohonen-type neural network [44]. The GPI-SOM software was trained using proteins from organisms of major branches of the evolution of eukaryotes. It included the predictions of SignalP 4.0 [45] for the presence of the signal peptide to reduce the number of false positives. GPI-SOM provides the position of the (ω) site and divides the proteins into three categories: non-GPI protein, GPI protein or GPI uncertain protein. FragAnchor uses a tandem prediction of a neural network and a statistical HMM to predict GPI-APs [46]. The neural network selects the putative GPI proteins whereas the HMM establishes the qualitative score. Unlike GPI-SOM and Big-PI, FragAnchor only takes the C-terminal end into account. FragAnchor does not predict the position of the (ω) site. Predictions using this algorithm classify proteins into four groups: very likely, likely, probable and potential false negatives. PredGPI is based on the combined use of a support vector machine (SVM) for the GPI-anchor signal and an HMM method for the position of the (ω) site [47]. The HMM model determines the signal composition of the GPI anchor region, while the SVM takes into consideration the characteristics of the N-terminus of the target protein which make PredGPI independent from SignalP. Among all these programs, PredGPI is the most widely used tool for the prediction of GPI-APs.

Vigilance against a resurgence of drug-resistance among the various *Plasmodium* species demands that we continually improve our understanding of these organisms. The present work sheds light on previously unrecognized *Plasmodium* GPI-APs. A software for the detection of GPI-APs based on features detection was developed, and a heuristic method was defined to choose the software parameters giving the best sensitivity and specificity. This approach was applied to the study of the proteome of 46 PlasmoDB isolates. For the first time, the comparison of the GPI-proteome between apicomplexan parasites demonstrates a new correlation with the evolution of *Plasmodium* parasites.

## Methods

### Sequences

All sequences of each of the 46 isolates were retrieved from PlasmoDB (sequences from release 51, Additional file 1: Table S1) using a written script. OrthoMCL annotation was updated using release 53 [48].

### FT-GPI development

**Fig. 1:**
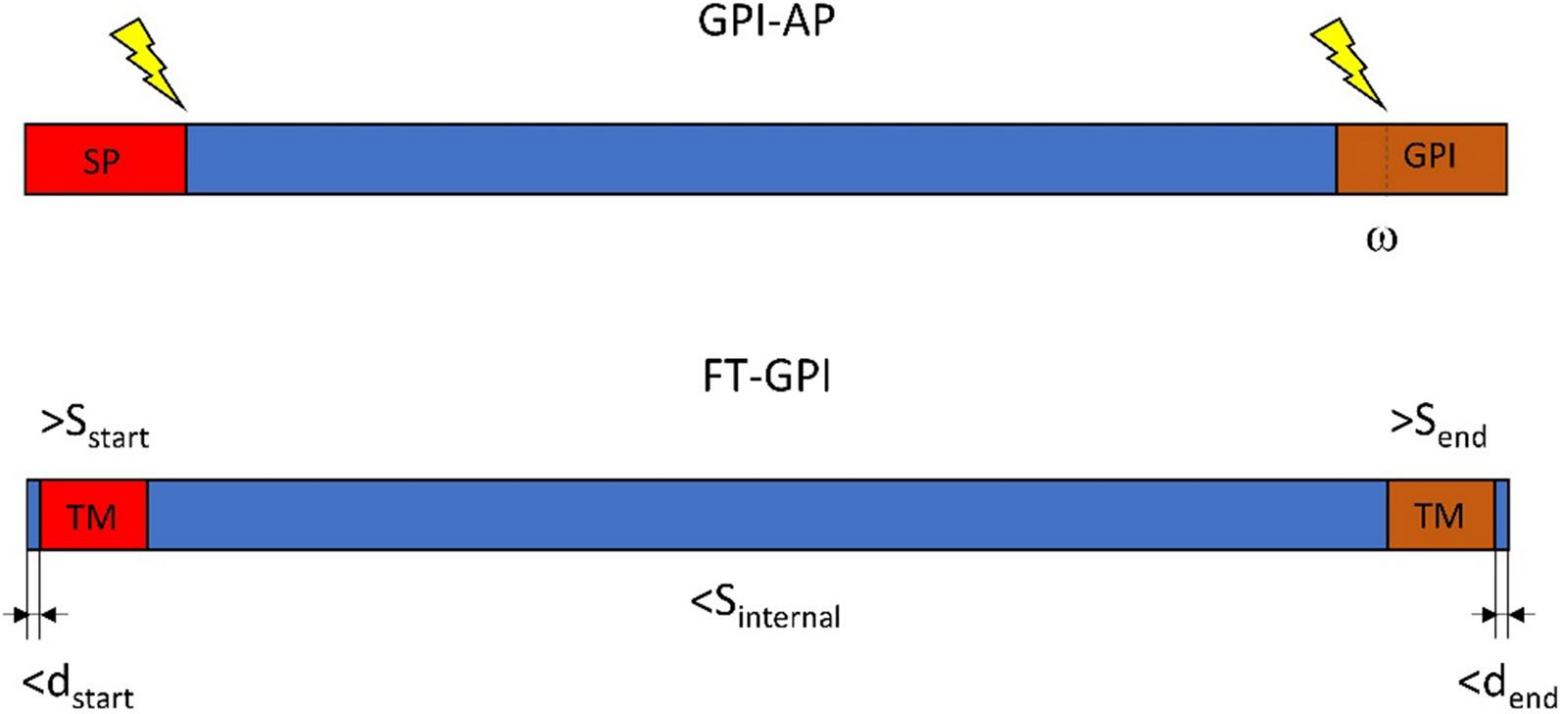
Summary of the main features of GPI-AP and FT-GPI TM. The N-terminal ER-targeting signal and C-terminal GPI-anchoring signal are generally associated with the ER-membrane via a hydrophobic helix. A mature GPI-AP peptide is produced after cleavage of the signal peptide and at site of the GPI-anchoring signals. FT-GPI software uses the TMPred score and distance to the protein extremity as identifying markers of hydrophobic regions at both extremities of the propeptide. The parameters S_start_, S_end_ and S_internal_ correspond to TMPred scores of the N-terminal, C-terminal and all internal hydrophic helices, respectively. They are controlled with -n, -c and -i options of the software. FT-GPI expects to encounter the first amino acid of the N-terminal region between the first residue and position d_start_ of the protein. FT-GPI expects to encounter the last amino acid of the C-terminal region after the d_end_ position on the protein

TMPred was recoded in C +  + and integrated in the FT-GPI detection software. The C +  + version was validated using the EMBOSS server at Expasy. Source files of TMpred are available at https://rcanovas.github.io/project/tmpred/. FT-GPI can be downloaded at https://github.com/rcanovas/FT-GPI. TMPred prediction of transmembrane domain (TM) are based on standard matrix to score the presence of hydrophobic alpha-helix and given in a table and graphical format. Trained programs, such as TMHMM [49] and DeepTMHMM [50] were used to predict TM. A marked difference in C-terminal TMs predicted by TMHMM and hydrophobic helices predicted by TMPred was observed. Almost all C-terminal TMs in PlasmoDB were predicted by TMPred, which was able to predict more C-terminal hydrophobic domains, especially among GPI-APs, compared to other programs. FT-GPI detection used TMPred output to filter TM features according to defined parameters (Additional file 2: Table S2). FT-GPI considered a candidate as likely to be a GPI-AP when it detected hydrophobic helices at the N- and C-terminal ends. These hydrophobic regions correspond to the signal peptide (SP) and GPI attachment sites, respectively (Fig. 1). The N-terminal region is expected to have a minimal score value S_start_ and a distance from the initial methionine of no more than d_start_. FT-GPI automatically runs the analysis from the second start codon (ATG) with the same d_start_ value if no signal is detected using the first methionine. P_end_ parameter defines the minimal value of the TM score at the C-terminal hydrophobic helix, which should also be not far than d_end_ from the end of the sequence. An internal TM should have a score higher than S_internal_ or will otherwise be considered as a hydrophobic helix. Some globular proteins can exhibit such regions at the center of their 3D structure. Furthermore, the length of the hydrophobic helix can be fixed using certain TMPred parameters. FT-GPI generates two files: one file contains the detected proteins according to the given parameters; and another file provides the possibly misdetected proteins using relaxed conditions. In this case, the distance between the TM and the protein end is multiplied by 1.5 and a 10% modification of the threshold is considered to reduce S_start_ and S_end_, and to increase S_internal_.

### Selection of parameters

In total, 7 FT-GPI parameters were selected based on their ability to significantly influence the detection of transmembrane (TM) domains (Additional file 2: Table S2). Each of these parameters defined in the reals or integers (for scores and coordinates respectively) could vary over a range of about ten units or more. Therefore, a heuristic method was developed for the selection of FT-GPI parameters. The 30 GPI-APs of *P. falciparum* 3D7 and *P. vivax* Sal-1 were taken as training sets [17, 38]. TMpred data were compared with reference software such as SignalP and PredGPI (see below). As such, it was possible to determine the minimum and maximum possible values for each parameter. The -t option of FT-GPI allows users to take a training set as input and select the parameters allowing the highest specificity (e.g., largest distance to the end and lowest score of N- and C-terminal hydrophobic regions). In the present study, and in consideration of the observations made in the first step, a more finely-tuned range of values was tested for the different parameters. The information about the origin of the exclusion of benchmark proteins provided by the FT-GPI error file was very useful for testing the relevance of certain parameter values. Consequently, a set of 31 combinations of parameter values (PLA000 to PLA030) were applied in this study (Additional file 2: Table S2). The selection of the most accurate set of parameters was first assessed using *P. falciparum* and *P. vivax* reference sets. The prediction of GPI-AP was compared with cytosolic membrane-associated proteins or the full proteome. The performance of each parameter set was then evaluated according to the ability of FT-GPI to detect orthologs of the *P. falciparum* reference GPI-AP in each of the selected species. Validation of PLA001 and PLA030 as the most sensitive and specific sets of parameters was done by comparing the GPI-proteome evolution with each species’ phylogenetic tree. It was important to consider sequence and annotation errors throughout the process.

### Bioinformatic analysis

FT-GPI prediction was compared with Signal 5.0 or 6.0 and PredGPI using online web sites [47, 51, 52]. FragAnchor and GPI-SOM were used online [44, 46]. FT-GPI prediction was combined with OrthoMCL analysis and synteny to validate the detection of GPI-Aps. Pairwise sequence comparisons were performed using BLAST at NCBI. Protein domain description was obtained using the online version of Pfam [53].

Jacard distance was computed to compare the composition of the GPI-proteome between species based on OrthoMCL annotation. To this end, the presence or absence of orthologs in the OrthoMCL groups presenting more than 4 isolates was transformed in a Boolean variable. The composition of the GPI proteome was characterized with regards to the different taxonomic groups that were selected (Additional file 3: Table S3). One *Hepatocystis* parasite and two species from the *Haemamoeba* subgenus were added to the isolates from the *Laverania*, *Plasmodium* and *Vinckeia* subgenus to complete the analysis of GPI-proteome and evaluate its evolution. Statistical analyses were performed in R.

## Results

### P. falciparum and P. vivax GPI-AP reference sets

Test sets of GPI proteins were generated from the analysis of GPI-AP provided by Gilson and colleagues on the *P. falciparum* 3D7 strain, and by Carlton and colleagues on the *P. vivax* Sal-1 strain (Table 1). These initial sets consisted of 30 GPI-AP proteins [17, 38]. The presence of transmembrane domains I and signal peptides (SP) in the sequence of the propeptide was obtained from PlasmoDB, which uses TMHMM for prediction of TMs. Sequences were examined using PredGPI [47] and SignalP 5.0 [52] (Additional file 4: Table S4). Two properties were essential to validate a GPI-AP candidate: 1) presence of a SP and 2) presence of a GPI-anchoring site (Fig. 1). These criteria are often associated with the presence of a hydrophobic helix that plays a role as a membrane anchor and stabilizes the protein in the luminal leaflet of the ER-membrane [51, 54]. The cleavage of the N- and C-terminal regions take place after stabilization of the interaction with corresponding proteases to generate the mature peptide. TMPred prediction for hydrophobic alpha-helix [55] was evaluated on the basis of these criteria (Additional file 5: Table S5).

**Table 1:**
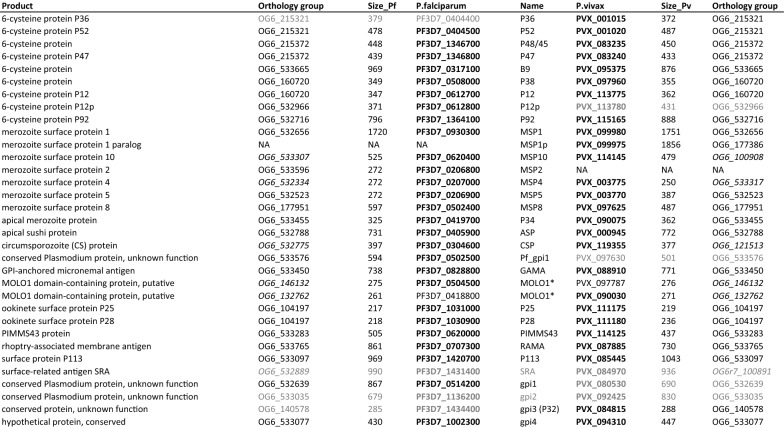
Reference GPI-AP sets of *P. falciparum* 3D7 and *P. vivax* Sal-1

The ID of reference GPI-APs are in bold. GPI-AP- orthologs predicted by FT-GPI are provided (NA: no available orthologs). Genes presenting discrepancy between synteny and OrthoMCL annotation are italicized (different OrthoMCL ID at a conserved locus between *P. falciparum* and *P. vivax*). Proteins which were negative according to FT-GPI detection by selected parameter sets (Additional file 2: Table S2) are in grey. Some proteins had no gene names and were arbitrarily labelled from gpi1 to gpi4. Pf_gpi1 was specific to the list given by Gilson, et al*.* [17]. The gpi3 protein was labelled as P32 in some references. *Genes encoding MOLO1-domain containing proteins from *P. falciparum* and *P. vivax* reference sets were paralogs and were named MOLO1 in the present study

**Fig. 2:**
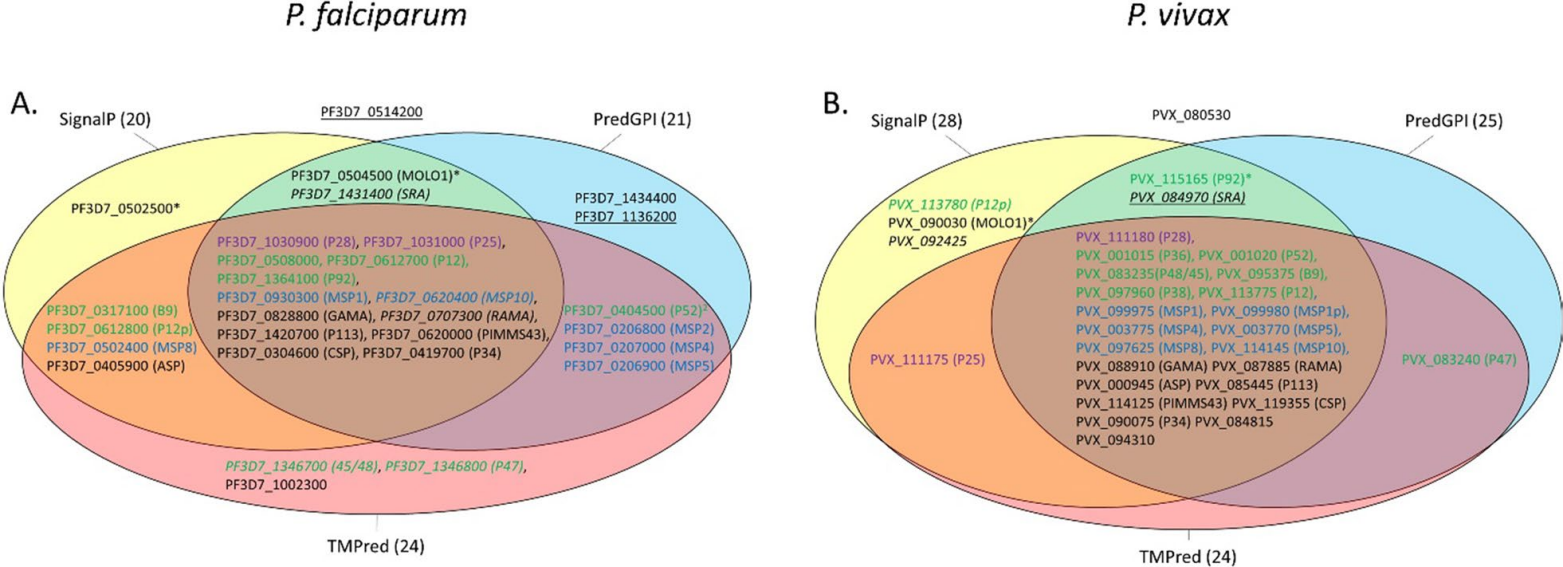
Classifications of reference GPI-APs *in Plasmodium falciparum* and *P. vivax* by three tools: PredGPI, SignalP and TMPred. **A**. *P. falciparum* GPI-AP reference set from Gilson et al. [17] analysis. **B**. *P. vivax* GPI-AP reference set from Carlton et al. [38] analysis. Gene names are given in brackets. Members of the 6-cystein protein family are coloured in green, MSP proteins in blue, and ookinete P25 and P28 proteins in violet. Proteins with an internal TM score > 1200 are underlined. Italics indicate proteins with 0.6 < SignalP 5.0 score < 0.75. The three tools disagreed at times regarding the prediction of the cleavage site for these proteins (Additional file 1: Table S1). C-terminal TM’s detected with d_end_ > 3 were labelled with *. A superscript label ^2^ indicated that the N-terminal TM was detected using the second ATG in ORF (a default option in FT-GPI)

The presence of both features at protein extremities suggest that the detection of a GPI-AP candidate depends heavily on the quality of the annotation. The sensitivity of PredGPI, SignalP and TMPred when detecting these motifs was variable (Fig. 2). One third (10/30) of *P. falciparum* predicted GPI-AP proteins had no predictable SP according to SignalP 5.0 while only three such proteins were found among the *P. vivax* GPI-APs. Nine of the documented *P. falciparum* and five of the *P. vivax* GPI-APs were not predicted by PredGPI. The absence of a consensus site was confirmed for 9 *P. falciparum* and 2 *P. vivax* GPI-APs. TMPred predicted a hydrophobic domain in the N-terminus of 26 out of the 30 recognized *P. falciparum* GPI-APs with scores ranging between 509 and 3175 (Additional file 5: Table S5). The same result was obtained for *P. vivax* proteins with a narrower score range, suggesting that the *P. falciparum* 3D7_0612800 score of 3175 is an outlier. All GPI-APs were positively predicted with TMPred for the presence of the hydrophobic domain at their C-terminal end. The TMPred hydrophobic helix scores ranged from 1140 to 2972 with larger variations in *P. falciparum*. Altogether, these observations suggest that the proteolytic process at both ends of the GPI-AP is facilitated by membrane anchoring.

TMpred predicted a hydrophobic helix in all 325/326 *P. falciparum* and *P. vivax* proteins presenting a C-terminal TM predicted by TMHMM, but TMPred was found to be more sensitive in predicting GPI-APs in *P. falciparum* (Fig. 2A). TMPred yielded very similar values for 24 proteins in both *P. falciparum* and *P. vivax,* suggesting that some proteins of the reference sets may not be GPI-Aps (Additional file 5: Table S5). For most proteins, the distance to the end of the last hydrophobic helix was between 0 and 3. Four proteins (2 for *P. falciparum* and 2 for *P. vivax*) had a membrane-anchoring signal located at 5 to 14 amino acids (aa) away from the C-terminal end (including *P. falciparum* and *P. vivax* MOLO1 domain-containing proteins). Some proteins had a predictable internal hydrophobic helix, and these secondary structures had a score ≤ 1000. PF3D7_0514200, PF3D7_1136200 and PVX_084970 had putative internal TM with TMPred scores greater than 1200. The N-terminus region of P52 6-cystein protein (PF3D7_0404500) was 20 residues away from the initial methionine, but close to the consensus after considering the second ATG in CDS. PF3D7_0514200 and PVX_080530 *gpi1* orthologs had no predictable SP nor a GPI-attachment site (Fig. 2A, B). The *gpi2* orthologs of PF3D7_1136200 and PVX_092425 had no N-terminal hydrophobic region. Meanwhile, PF3D7_1434400 (*gpi3*) and PVX_113780 *(p12p*) lacked GPI-AP signature. TMPred analysis suggests that these proteins are unlikely to be GPI-APs.

Noteworthy, there was limited correlation between PredGPI or SignalP prediction and TMPred scores. For those proteins predicted not to be GPI-APs (GPI-AP-) by TMPred, the SignalP 5.0 score was also less than 0.7 (Additional file 4: Table S4), but some GPI-APs such as MSPs had very low score, highlighting the difficulty of SignalP in identifying candidates in *P. falciparum* in general. The TMPred negative prediction of well characterized surface-related antigen (SRA) orthologs [56, 57] was intriguing. In fact, SRA proteins had a TM downstream of the SP putative cleavage site. Furthermore, selection of the second or third methionine in P52 showed a slightly improved score for a putative SP by SignalP 6.0 [51]. The experimental evidence that P52 is a functional GPI-AP protein suggests that this protein might employ a different signal for its targeting to the ER. The FT-GPI program was thus developed to address specific shortcomings of the existing protein analysis toolchain.

### Feature-based detection of GPI-APs

FT-GPI is a feature-based GPI-AP detection software that searches for the presence of a hydrophobic helix at both ends of the protein and nowhere else (Fig. 1). The program determines the position (d) and score (S) value for each membrane-anchoring region of a GPI-AP propeptide. In a first attempt to optimize the detection of GPI-APs, the parameters were set near the minimum and maximum values given in Additional file 5: Table S5. TMPred predicted scores for hydrophobic regions were generally smaller for the N-terminus than for the C-terminus, but no discernible pattern could be derived from this analysis to be programmed into FT-GPI. The -p option of FT-GPI was not used, as no correlation with protein size was observed. Different parameter settings were used based on the assumption that a combination should be able to detect all GPI-APs (Additional file 2: Table S2). PF3D7_0404500 was detected by all combinations of FT-GPI parameter values tested after the second ATG was automatically considered by the software. Three *P. falciparum* and four *P. vivax* proteins were not detected in the reference sets. Setting S_internal_ at 1200 or below led to rejection of PF3D7_0514200. FT-GPI parameters were also set to reduce the number of false positives. In fact, FragAnchor, GPI-SOM and PredGPI predictions on the full proteomes highlighted the significantly high false positive rate and the major challenges associated with these predictions. The three algorithms predicted 240, 355 and 55 GPI-APs from the *P. falciparum* proteome and 285, 265 and 84 GPI-APs from the *P. vivax* proteome, respectively. The parameter sets used in the present study generated a number of predicted GPI-APs in the range of PredGPI, that varied from 31 to 50 for *P. falciparum* and 30 to 48 for *P. vivax*. The *P. falciparum* SRA-antigen (PF3D7_1431400) was detected only by the parameter sets of PLA025 or and PLA026. However, tuning the parameters to improve SRA-antigen detection produced too many false-positive results. Thus, it remains unclear whether the *Plasmodium falciparum* and *P. vivax* SRA proteins are functional GPI-APs.

The sensitivity and specificity of the various parameter sets (Additional file 2: Table S2) was assessed by cross-referencing the 30 previously predicted GPI-APs of *P. falciparum* and *P. vivax* against known cytosolic proteins, membrane proteins, and the entire proteomes of these parasites (Additional file 6: Table S6). Optimal AUC and sensitivity were generated with the parameter sets PLA011 and PLA012, which detected 26/30 in *P. falciparum* and 25/30 in *P. vivax*. The missing *P. falciparum* proteins were PF3D7_0514200, PF3D7_1136200, PF3D7_1431400 and PF3D7_1434400 for the reasons outlined above. PLA001 and PLA030 values were some of the most specific sets of parameters with slight differences between *P. falciparum* and *P. vivax* (Additional file 6: Table S6). The lower sensitivity of PLA001 and PLA030 was for the C-terminus end of 4 proteins (PF3D7_0502500, PF3D7_0504500, PVX_090030 and PVX_115165). Increasing d_end_ to 4 or more (such as PLA011) increased the number of false positives. PVX_090030 was detected only by the PLA026. PredGPI suggested that this protein may not be a GPI-AP (Additional file 4: Table S4). The interest of PLA001 and PLA030 parameter sets was confirmed by comparing the reference sets of GPI-AP with all putative GPI-APs detected by all combinations of FT-GPI parameters (Additional file 6: Table S6). Lower AUC of PLA011 and PLA012 in this experiment suggest that many of the false positives may indeed not be GPI-APs. The GPI-proteome of *P. falciparum* and *P. vivax* was thus reannotated on the basis of information provided by FT-GPI.

**Fig. 3:**
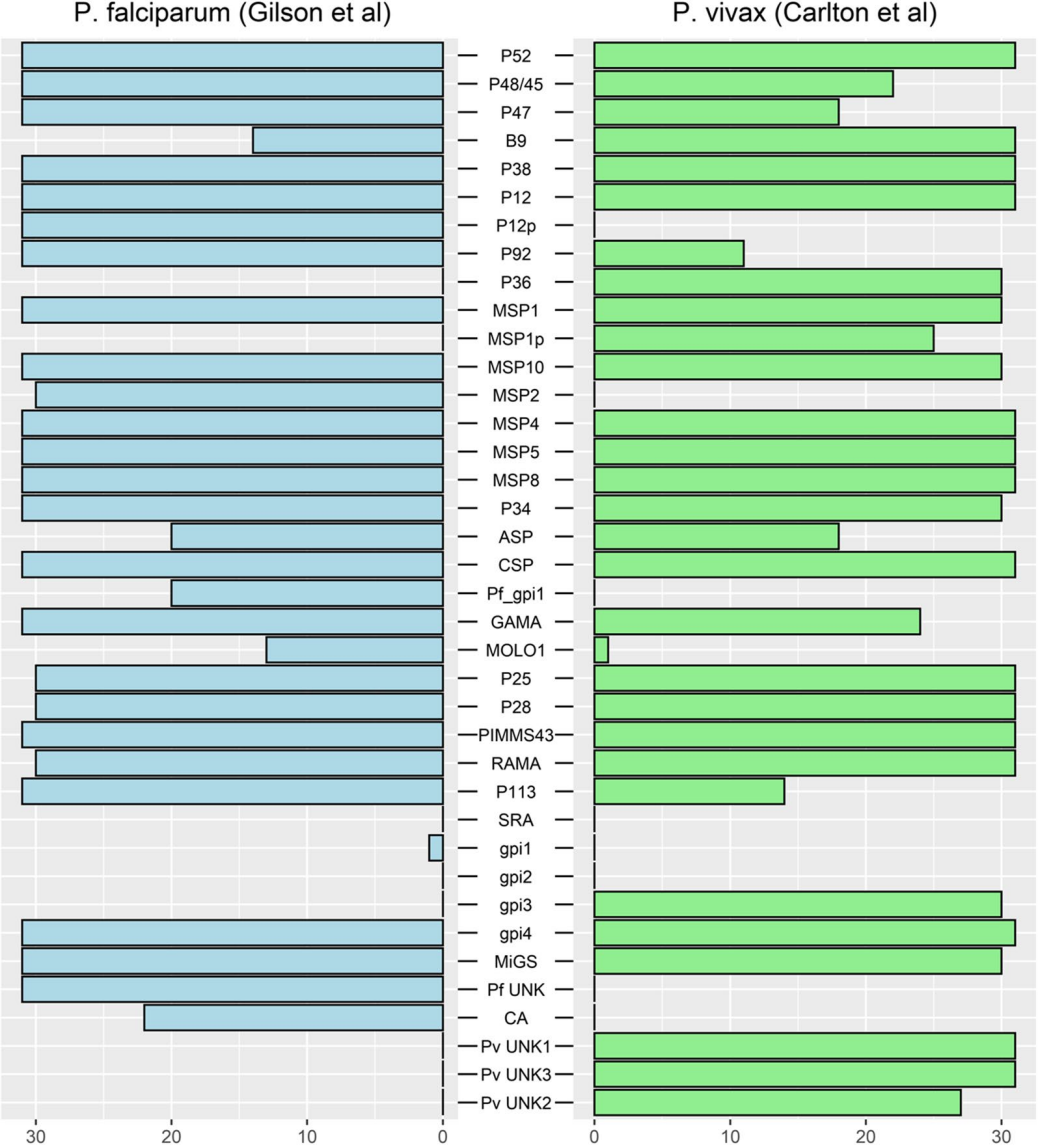
*Plasmodium falciparum* and *P. vivax* FT-GPI-detection of reference GPI-AP. Each protein listed in the reference set (Table 1) was tested using 31 different combinations of FT-GPI parameter values (Additional file 2: Table S2). For each protein, the horizontal-colored bar depicts the distinct number of parameters sets yielding prediction of GPI-AP + (as given in Additional file 6: Table S6). Of particular note for follow-up study are the six proteins at the bottom of the list, which are new putative GPI-APs detected by FT-GPI and discussed herein. MSP1p from *P. vivax* and MSP2 from *P. falciparum* had no ortholog in the corresponding species. *Plasmodium falciparum* P36 ortholog (not shown) was not in the list reported by Gilson, et al*.* [17] and was classified as GPI-AP- by all FT-GPI parameter sets. Some proteins had no gene names and were arbitrarily labelled from gpi1 to gpi4. The *P. vivax* ortholog of Pf_gpi1 was absent from the list reported by Carlton, et al*.* [38] and was also GPI-AP- using FT-GPI analysis. The gpi3 protein was labelled as P32 in some references. FT-GPI classified all SRAs as GPI-AP- with all parameter sets. MOLO1_domain-containing protein encoding genes in *P. falciparum* and *P. vivax* reference sets were in fact paralogs. CA: carbonic anhydrase; UNK mean that the protein was of unknown function t

**Fig. 4:**
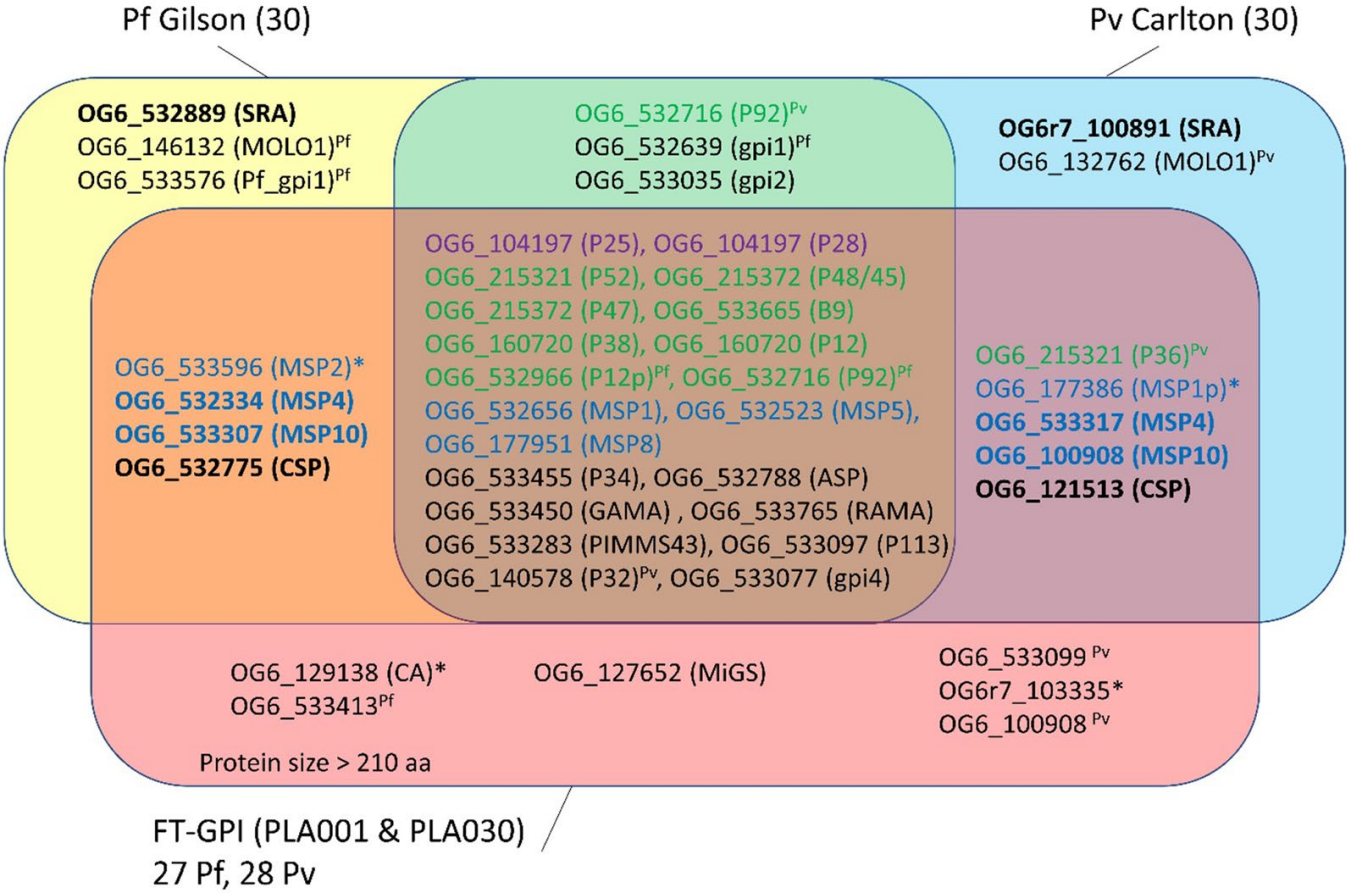
*P. falciparum* and *P. vivax* proteins classified as GPI-AP + by FT-GPI when using both PLA001 or PLA030 parameter sets (which tended to be the most restrictive sets). A set of 27 GPI-APs was confirmed in *P. falciparum* 3D7. There were 28 confirmed GPI-APs in *P. vivax* Sal-1 analysis. Twenty-one proteins were conserved between the initial prediction of *P. falciparum* and *P. vivax* [17, 38] and FT-GPI detection using PLA001 or PLA030. *Plasmodium falciparum* label is for *P. falciparum* proteins whose ortholog in *P. vivax* is not GPI-AP. *Plasmodium vivax* indicated *P. vivax* proteins with ortholog in *P. falciparum* that was not GPI-AP. Proteins with no ortholog in *P. falciparum* or *P. vivax* are marked with *. Proteins expressed at a conserved locus, but with different OrthoMCL ID, are in bold. The *P. falciparum* 3D7 GPI-proteome is composed of 24 previously described GPI-APs [17] and three new putative GPI-APs. The *P. vivax* Sal-1 GPI-proteome composition encompassed 24 previously-identified GPI-APs [38] and four new proteins. The 6-cystein protein and corresponding OrthoMCL IDs are in green. Members of the MSP multigene family are in blue. Ookinete proteins are in violet

The GPI-proteomes of *P. falciparum* and *P. vivax* share 28 orthologs (Table 1 and [38]). The sensitivity and specificity of FT-GPI was evaluated for each protein (Fig. 3). FT-GPI was able to correctly identify eight 6-cystein proteins in *P. falciparum* and *P. vivax*. All, except *P. vivax* P92, were identified by PLA001 and PLA030 combinations of parameters). This subset of GPI-APs differs on P12p and P36, whose respective orthologs are not GPI-APs (Fig. 4). Accordingly, *P. vivax* P12p (PVX_113780) had a weak SignalP signal and PredGPI score. The GPI-proteome of both *Plasmodium* species contained 6 MSP proteins. MSP2 is known to be restricted to the *Laverania* subgenus [58]. In the same manner, the *msp1* gene had a paralog, (*msp1p)* only in *P. vivax* [59]. A group of nine well-annotated GPI-APs were detected by FT-GPI (GPI-AP +): ASP, CSP, GAMA, RAMA, PIMMS43, P34, P113 and the two ookinete surface paralogs P25 and P28. The SRA proteins were incorrectly rejected as GPI-AP candidates because of their unusual N-terminal ER-targeting signal, as described above. Additionally, gpi4 hypothetical conserved protein was predicted to be a GPI-AP candidate in both species (OrthoMCL orthology group OG6_533077).

Our analysis further sheds light on the evolution of the *P. falciparum* and *P. vivax* GPI-proteome. PlasmoDB reports that *msp1p* and *msp2* are the result of a local tandem duplication, indicating that the protein-driven cell targeting mechanism in some GPI-APs (and their paralogs and/or orthologs) may be under evolutionary selection. The P36 and P52 6-cystein proteins are paralogs at the same location and same orientation on *Plasmodium* genomes. The PVX_001015 labelled as P36 by Carlton, et al*.* (Additional file 4: Table S4 and [38]) is a paralog present only in parasites from the subgenus *Plasmodium* (Additional file 3: Table S3). PVX_001015 and P52 orthologs diverged from P36 orthologs by the presence of a predictable signal peptide. A divergence in the first 12 amino acids may explain why the *P. falciparum* gpi3 ortholog (OG6_140578, PF3D7_1434400) was not predicted as a GPI-AP by FT-GPI. PVX_097630 gpi1 ortholog diverged at both extremities of the proteins from *P. falciparum* (OG6_533576, PF3D7_0502500) and was not predicted as GPI-AP by any software (GPI-AP-). It should be noticed that PF3D7_0502500 was GPI-AP + with more than 20 FT-GPI parameter sets, but PLA001 and PLA030 were not due to the value of d_end_ (Additional file 4: Table S4, Additional file 5:Table S5). Sequence evolution and shared domains often made elucidation of the relationship among OrthoMCL annotation, synteny, and GPI-AP ± challenging. MSP4 of *P. vivax* and its orthologs in other species were grouped with a family of serine/threonine kinases for unclear reasons. The OG6_100908 group of *P. vivax* MSP10 comprised several paralogs in a dozen of *Plasmodium* isolates. Each MOLO1-domain protein in the reference datasets had a paralog in different OrthoMCL groups (OG6_132762 and OG6_146132), which was located at a different place in the genome. PF3D7_0504500 was one of four MOLO1-domain proteins found in this study to have a “Probable” PredGPI score.

Finally, FT-GPI parameter combinations PLA001 and PLA030 predicted 24 GPI-APs in agreement with previous analyses performed in *P. falciparum* and *P. vivax* (Fig. 4), with 21 of these 24 GPI-APs common to both species. *Plasmodium falciparum*-specific GPI-APs were P12p and P92 6-cystein proteins and MSP2. P36, MSP1p and OG6_140578 ortholog (PVX_084815) were specific to *P. vivax*. Overall, these results highlight the high sensitivity and specificity of the FT-GPI parameter combinations PLA001 and PLA030.

### Novel P. falciparum and P. vivax GPI-APs predicted by FT-GPI

Although highly specific, PLA001, PLA030 and other parameter combinations detected new GPI-APs in addition to those described previously in the reference sets. However, 25% of the GPI-AP candidates identified by FT-GPI were smaller than the ookinete surface protein P25, which itself was the smallest GPI-AP described so far in any *Plasmodium* species with 217–218 aa. Size distribution of GPI-APs generated by UNIPROT showed a bimodal distribution that could be modeled well with the sum of two normal distributions (Additional file 10: Fig. S1). The first percentile for the larger normal distributions was approximately 223 residues. This was very close to the size of P25, which corresponds to one-third of all GPI-APs, but 25% of the reviewed GPI-APs in UNIPROT, including smaller ones. None of the 314 reviewed small UNIPROT proteins had significant homology (as reported by BLAST) with *Plasmodium* proteins. Therefore, proteins below the size of the P25 ookinete protein should be analyzed with caution. So, although *P. falciparum* site appears to be very different from the consensus, the presence of an amino acid characteristic of this site was observed at the expected position in PF3D7_0114500 and PF3D7_1477100 proteins that belong to the *hyp* multigene families [60].

As the most restrictive parameter combinations, the candidates identified as GPI-AP + by PLA001 and PLA030 are also each identified as such by all other parameter combinations. The most significant new GPI-AP + candidates were the microgamete surface protein MiGS present in *P. falciparum* and *P. vivax* (OG6_127652), two *P. falciparum* specific GPI-AP (the carbonic anhydrase, PF3D7_1140000, and one protein of unknown function) and three *P. vivax* GPI-AP, all of unknown functions (Figs. 3, 4). The most striking difference between *P. falciparum* and *P. vivax* was the presence of the carbonic anhydrase (CA) among *P. falciparum* GPI-APs. Many parameter combinations incorrectly identified the GPI-anchor transamidase as GPI-AP + , but transamidase was correctly identified as GPI-AP- by PLA001 and PLA030. Despite very good prediction by FT-GPI, the ER-Golgi proteins Kish and Yos1 (72 and 76 residues, respectively) and the TM protein TMEM222 (OG6_103330, 172 aa) were manually removed from the list of GPI-APs based on their function and small size (as discussed previously).

Rifin genes encode membrane proteins with one SP and either one or two TMs at their C-terminal end [61]. No previous results supported the existence of GPI-anchored members in these families, but some truncated members could be classified GPI-AP + by FT-GPI with certain parameter combinations. No consensus site could be observed for these proteins. Interestingly, PVX_113245 was classified GPI-AP- by PLA030 because of a long C-terminal TM (25 residues). suggesting possible TM-based GPI-AP partitioning of proteins, which is a common feature in some ER-specific processes [62]. In conclusion, the GPI-proteomes of *P. falciparum* and *P. vivax* are highly conserved in size (27 and 28 GPI-AP + candidates, respectively) and composition (22 conserved orthologs).

### Evolution of the GPI-proteome among Plasmodium species

**Fig. 5:**
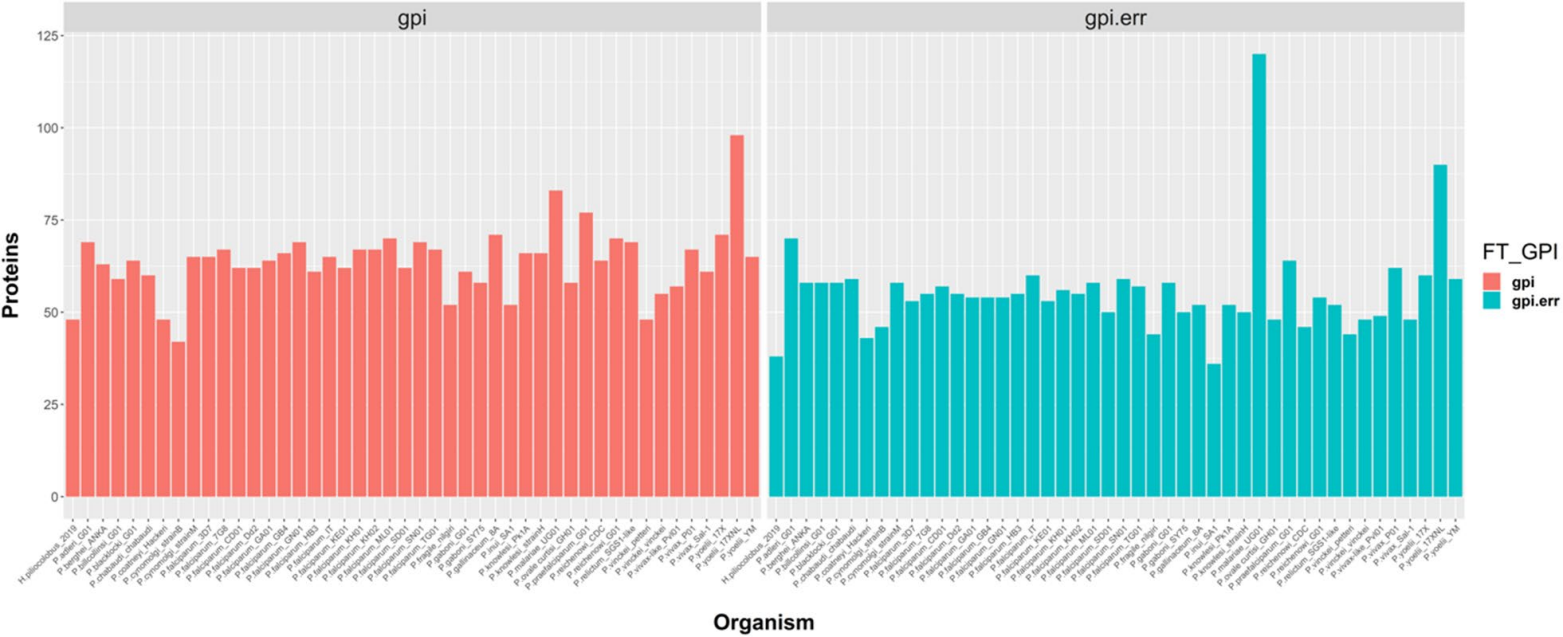
Total number of proteins classified as GPI-AP + by FT-GPI. Proteins were classified as GPI-AP + or GPI-AP- using the combinations of parameters described in Additional file 2: Table S2, collected in a list with duplicates removed, and counted (Y-axis). FT-GPI generated two files. The result file listed putative GPI-AP candidates (left panel). The error file (right panel) summarized proteins that failed to be detected for one parameter and used more relaxed conditions to suggest possible changes of the gene model or parameters used in combination. The number of proteins classified as GPI-AP + varies with choice of parameters from 13 to 32 (Additional file 7: Table S7)

**Fig. 6:**
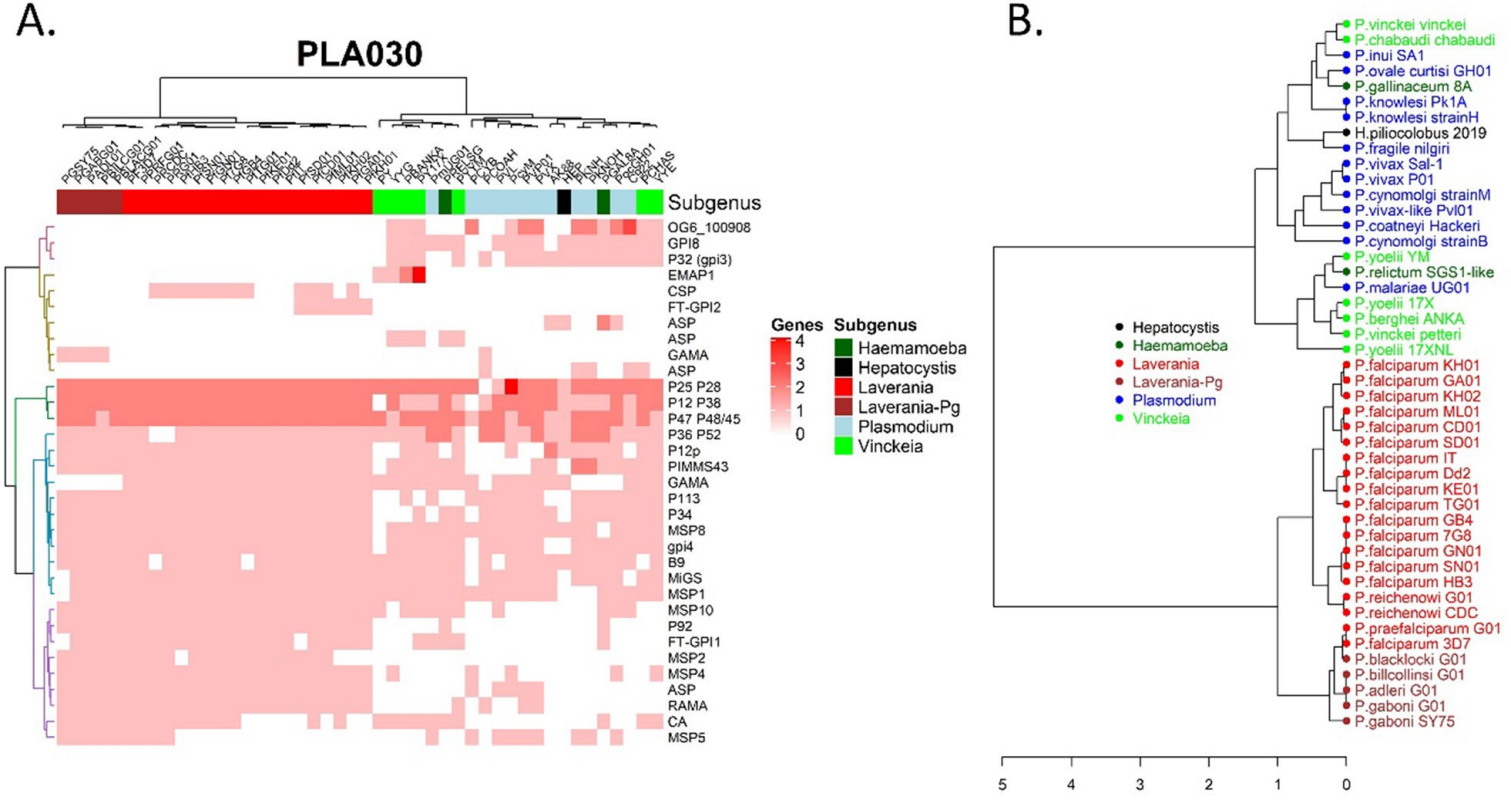
Using FT-GPI configured with PLA030 to guide investigation of the evolution of the GPI-Proteome among Haemosporida. Gene-encoding proteins with a size over 210 aa were selected for this analysis. **A**. Heatmap representing the distribution of genes among species. The presence of orthologs and paralogs was established using OrthoMCL annotation. Only orthology groups presenting orthologs in more than four species were included in the present analysis. The presence of paralogs were detected for some genes and are quantified in red. A GPI-AP was absent (white) either because it was not detected by FT-GPI using PLA030, or the gene was absent from the genome. Some genes were represented by more than one OrthoMCL group. The discrepancy between synteny and orthology groups may be due to rapid sequence evolution and shared homologies. The complete species name is given in panel B and Additional file 3: Table S3. Laverania-Pg differentiated *P. gaboni* and close species from the *P. falciparum*/*P*. *reichenowi* group of parasites [68]. **B**. Evolution of the GPI-proteome is related to speciation. With the same sets of genes as in Panel A, the number of paralogs was set to 1 to compute Jaccard distance. The Ward-2 method was used to build the tree

Composition of the GPI-proteome was further analyzed in 46 Haemosporida isolates, including *P. falciparum* 3D7 and *P. vivax* Sal-1 strains described above. FT-GPI detected 2932 GPI-AP + candidates among 252,551 proteins. The total number of GPI-AP + candidates was low in organisms such as *Hepatocystis piliocolobus* and *Plasmodium cynomolgi* strain B (Fig. 5). The ability to detect orthologs of the *P. falciparum* 3D7 reference set of GPI-APs agreed with the initial prediction, except for *Plasmodium yoelii* 17XNL strain, which presented the highest number of detected GPI-APs (between 38 and 75 GPI-AP + candidates, depending on choice of parameter combinations, Additional file 7: Table S7) and one of the lowest numbers of predicted orthologs (Additional file 10: Fig. S2). Genome assembly and annotation partially explained this variation. No relationship could be established between the GPI-proteome composition of 17XNL clone and its selection process [63]. Ookinete P25 and 28 proteins had highly conserved size, but genome annotation of *Plasmodium coatneyi* Hackeri fused both genes in one model, leading to a predicted protein of 429 residues. In the genome annotation of *P. cynomolgi* strain B, P25 was spliced into two genes. For the comparative analysis of GPI-proteomes and according to the observations above concerning the unlikelihood of extremely small proteins being GPI-AP + in *P. falciparum* and *P. vivax*, small proteins were filtered out, eliminating close to 25% of the proteins, 748 out of 2932 candidates. The remaining 2184 GPI-AP candidates were distributed among 269 OrthoMCL groups. The parameter combinations PLA001 and PLA030 identified GPI-AP + candidates belonging to 146 and 149 OrthoMCL groups, respectively (Additional file 8: Table S8, Additional file 9: Table S9). One third of proteins in a particular OrthoMCL group included proteins from the *P. yoelii* 17XNL clone. A heatmap representation is provided to show the distribution of GPI-AP + candidates among the most conserved OrthoMCL groups (Fig. 6A and Additional file 10: Fig. S3A for PLA030 and PLA001, respectively).

All 24 validated GPI-APs from the *P. falciparum* and *P. vivax* reference sets (Fig. 4), as well as MiGS and the two additional candidate *P. falciparum* GPI-APs (CA and OG6_533413 labelled as FT-GPI1), had orthologs in a significant number of isolates. *Plasmodium vivax*-specific *msp1p*, PVX_086272 and PVX_115460 had orthologs in OrthoMCL groups with four or fewer GPI-APs. The *msp1p* gene was found in species from *Haemamoeba* and *Plasmodium* subgenera after combining annotations with synteny (Additional file 8: Table S8, Additional file 9: Table S9). PVX_115460 orthologs were restricted to the three *P. vivax* isolates. The *P. falciparum* 3D7- and *P. vivax* Sal-1 CSP-associated OrthoMCL groups consist of less than four proteins. The largest group of CSP orthologs encompassed 12 proteins encoded by the genome of *P. falciparum* isolates and closely-related species, such as *Plasmodium reichenowi*. The detection of SRA proteins in *Haemamoeba* species, as well as in all *Plasmodium gaboni* and *P. yoelii* isolates, suggests the possible evolution of the N-terminal region of this protein in other parasites. MOLO1 domain-containing proteins were detected by FT-GPI PLA001 and PLA030 parameter sets in only one organism (different proteins from different OrthoMCL groups), confirming that this group of putative antigens are in fact not GPI-Aps. *Laverania* subgenus was associated with the presence of MSP2 and the specific prediction of P92 (Fig. 6A and Additional file 10: Fig. S3A). Furthermore, P92 was predicted as GPI-AP in the two *Haemamoeba* isolates as well. The *Vinckeia* subgenus was characterized by the absence of MSP5 along with the presence of EMAP1 GPI-AP orthologs and paralogs. Analysis of 46 isolates confirmed the presence of four new GPI-AP labelled as FT-GPI1 to 4, FT-GPI2 and 4 being absent from *P. falciparum* 3D7 and *P. vivax* Sal-1.

OrthoMCL groups with proteins in more than four isolates (Additional file 8: Table S8, Additional file 9: Table S9) were used to compute a binary distance and identify a possible relationship between speciation and composition of the GPI-proteome. The classification of GPI-proteomes deduced from this distance matrix was highly consistent with the species tree (Fig. 6B and Additional file 10: Fig. S3B). Various evolutionary events could be observed including gene duplication or deletion and a shift in the CDS, allowing TM proteins to become GPI and vice versa. The internal evolution of GAMA and ASP sequences could have helped separate the *P. falciparum* and *P. gaboni* groups of *Laverania* species (Fig. 6A). The prediction of P92 as GPI-AP was associated with the *Laverania* and not *Plasmodium* subgenus, but the gene was absent in *Vinckeia* isolates. P36 was not a GPI-AP in *Laverania*, but the gene encoding this protein and its paralog P52 were present in all genomes. PLA001 was more sensitive than PLA030 in classifying P52 as GPI-AP + in *P. reichenowi* isolates.

The present study provides a more complete description of the diversity of GPI-anchored MSP proteins, confirming many observations, including the following: Synteny confirmed that (1) *msp1* duplication leading to *msp1p* was absent in the *Laverania* subgenus as suggested before [64], and (2) the genes were assembled in different, small OrthoMCL groups. The rapid evolution of the protein sequence led to similar observations in MSP2, 4, 5 and 10. Nevertheless, MSP2 was restricted to the subgenus *Laverania*. Putative MSP2 proteins were only 162–164 aa in length in *Haemamoeba* parasites (OG6r7_367261 and OG6_117344). Three putative MSP4 were less than 210 aa in size, including genes annotated as *msp4/5* in *Plasmodium relictum* SGS1-like (209 aa), *Plasmodium chabaudi chabaudi* (209 aa) and *Plasmodium berghei* strain ANKA (201 aa). Further work is needed to investigate the cellular targeting of these putative proteins. Should this be confirmed, *Plasmodium gallinaceum* and *P. relictum* SGS1-like would have the most complete set of GPI-AP MSPs. The locus overlapping the MSP2, MSP4 and MSP5 genes was highly conserved in *Laverania* parasites, suggesting sequence or annotation errors of MSP5 encoding genes in all *P. falciparum* isolates outside 3D7. In MSP5 gene of *P. falciparum*, a second intron was found to overlap with the expected ATG start codon in the gene model. This annotation error results in an altered amino acid composition in the N-terminus region, of the encoded protein leading to its classification as GPI-AP- by all FT-GPI parameter combinations. Conservation of these sequences among these parasites precludes most other explanations.

The CA of *P. falciparum* is a protein of about 600 aa. The C-terminal part is highly conserved and characterized by the presence of Pfam domain (Carb_anhydrase). The corresponding OrthoMCL group OG6_129138 contains all expected 26 proteins, one per isolate including the 16 *P. falciparum* isolates. FT-GPI did not detect the N-terminal hydrophobic region of CA in 8/16 *P. falciparum* isolates (Additional file 8: Table S8, Additional file 9: Table S9). In fact, the region encoding the SP was absent in these proteins, presenting a size of 524 aa. As related above, this shows how bioinformatic detection of GPI proteins is very sensitive to the specification of the correct START codon. DNA sequence analysis revealed that, in the genome of isolates expressing the shorter form of CA, an extra A was inserted into the polyA sequence which in the 3D7 reference genome consists of 25 nucleotides. Furthermore, a 3 bp insertion could be observed in isolates CD01 and GN01, resulting in a single-lysine extension of the lysine residues encoded by the polyA sequence and true classification as GPI-AP + by FT-GPI. The absence of frameshift in these natural variants suggests a possible sequencing error in the genomes missing GPI-AP CA. That this is the only discrepancy between these species and the 3D7 sequence bouys the sequencing error hypothesis. *Hepatocystis* sp. CA had a very long N-terminal extension without SP. The large open reading frame of the gene lacks ATG, suggesting that the sequence was not complete. The gene is in fact at the extremity of a 17 kbp contiguous region and was absent from genomes of the *Plasmodium* subgenus. The evolutionary event can be classified as a gene loss considering the taxonomic position of this group of phylogenetically-related organisms.

In conclusion, the GPI-proteome is conserved among *Plasmodium* species with 25 to 30 GPI-APs in most isolates and a total of 37 different GPI-AP + candidates identified in Haemosporida parasites by FT-GPI.

## Discussion

### FT-GPI detection software

The FT-GPI software, described herein, estimates the likelihood that a candidate protein anchors to GPI with high sensitivity and specificity. Those proteins assigned a likelihood above a certain threshold are classified as GPI-AP + , and those assigned a likelihood below the threshold are classified as GPI-AP-. The likelihood estimate is driven by a combination of parameters (labeled PLA001 through PLA030); these parameters inform the model as to the relationship between protein structure and the likelihood that the protein anchors to GPI. In the present study, it is assumed that there exists one particular set of parameters that maximize both sensitivity and specificity. The heuristic method defined to select the most sensitive and specific sets of parameters was illustrated by the analysis of 46 proteomes of the order *Haemosporida*. One advantage of FT-GPI is that the values of the parameter have biological relevance, which helps make interpretation of the results possible. In fact, FT-GPI is the first GPI-AP detection software based on select protein annotation features (*i.e.* SP and GPI anchoring signal). In this work, the two features considered for the detection of GPI-APs were the N- and C-terminal cell targeting domains, working under the hypothesis that the N-terminal domain acts as a signal peptide and addresses the protein to the endoplasmic reticulum, while the C-terminal domain, with which the transamidase complex interacts, enables the protein to be linked to the GPI anchor after a specific cleavage reaction. The hydrophobic helices associated with these N- and C-terminal domains may be essential for the protein maturation process. These domains are often too small and/or divergent to be predicted as TM by trained methods such as TMHMM [49, 50]. Therefore, the N- and C-terminal hydrophobic regions predicted by TMPred were used as markers for the features. The absence of internal hydrophobic helices was also taken into consideration for GPI-AP classification. To this end, seven FT-GPI parameters were combined to describe the positions and hydrophobicities of these regions. Present analysis suggests that the C-terminal end of the protein is more hydrophobic than the end associated with the signal peptide in Haemosporida parasites. This enabled FT-GPI classification to achieve higher sensitivity with respect to the *P. falciparum* reference GPI-AP list than did SignalP, which incorrectly classified one-third of the same reference proteins as GPI-AP- (Additional file 1: Table S1). PredGPI showed lower sensitivity than FT-GPI on the same *P. falciparum* reference set as well, with 80% overlap with SignalP proteins classified as GPI-AP + . The presence of the C-terminal hydrophobic region was present in all GPI-AP + candidates identified by TMpred, but the sensitivity and specificity of FT-GPI was reliant on the d_end_ parameter measuring the distance of the helix to the end of the protein. Fixing d_end_ at 4, such as in PLA030 (Additional file 2: Table S2), endowed FT-GPI with a higher specificity. Another important criterion for FT-GPI was the TMPred score describing the central hydrophobic helix. Setting S_internal_ = 1200 appears to offer the best compromise between specificity and sensitivity.

We initially set out to validate our approach (that is, configuring FT-GPI using the above-described heuristic method to select the optimal parameter combinations), using GPI-AP reference sets described by Gilson, et al. for *P. falciparum* 3D7 [17] and Carlton, et al*.* for *P. vivax* Sal-1 [38]. We ultimately validated this approach by comparing the evolution of the GPI-proteome composition and species tree (Fig. 6B and Additional file 10: Fig. S3B). FT-GPI configured with a number of parameter combinations was used to analyse the proteomes of 46 isolates of *Plasmodium,* Proteins with greater than 210 residues were selected (Additional file 8: Table S8, Additional file 9: Table S9). FT-GPI classified 1,130 and 1,141 candidates as GPI-AP + using the parameter combinations PLA001 and PLA030, respectively. The estimated size of the GPI-proteome ranged from 14 to 32 with these two sets of parameters, with 75% of the 46 isolates having between 25 and 30 GPI-AP + candidates (33/46 isolates between 25 and 30 candidates for PLA001 and 34/46 isolates with between 25 and 30 candidates with PLA030). FT-GPI’s classification performance was highly dependent on the quality of the genome assembly and/or annotation. For example, we described above how a possible sequence error could have strongly affected the detection of CA. Sequence validation is needed for those *P. falciparum* isolates proteins not classified as GPI-AP + by FT-GPI. MSP5 gene models may also need to be confirmed by transcriptomic analyzes in most *P. falciparum* isolates. Partial genome assembly or annotation could be responsible for the incomplete description of the GPI-proteome of *H. piliocolobus*, *P. coatneyi* Hackeri, *P. cynomolgi* strainB and *P. yoelii* 17XNL. The data obtained using FT-GPI analyses suggest that further annotation improvements may be needed for these genomes.

The biological relevance of FT-GPI's parameters also made possible an informative comparative analysis of 46 Haemosporida isolates. Parameter combinations PLA001 and PLA030 classified as GPI-AP + 37 candidates in 33 OrthoMCL groups. Four OrthoMCL groups included two GPI-AP + candidates each. GPI-AP + ookinete proteins P25 and P28 and 6-cystein proteins P12, P38, P47 and P48/45 were present in nearly all isolates. The P36 protein from the OrthoMCL group OG6_215321 corresponded to orthologs in parasites from *Haemamoeba* and *Plasmodium* subgenus only, whereas a P52 paralog was ubiquitously present. There were 21 GPI-AP + candidates in common between *P. falciparum* 3D7 and *P. vivax* Sal-1. A total of 22 GPI-AP + candidates were conserved in most species including the microgamete surface protein MiGS. We conducted synteny analysis for each locus expressing a GPI-AP + candidate (Additional file 8: Table S8, Additional file 9: Table S9). Some orthologs were found in different OrthoMCL groups. The highest number of OrthoMCL groups was associated with the subgenus *Plasmodium*. This may be due to more distantly-related species and/or the rapid evolution of the gene sequences. Conversely, there were very few examples of OrthoMCL groups overlapping different functions. This point specifically concerns the OG6_100908 group, whose proteins could be separated between FT-GPI3, MSP10 and RAMA based on synteny analysis. The SRA protein was the most interesting false GPI-AP- result of FT-GPI, which rejected it on the basis of the evolution of its N-terminal end and a possible alternative process of ER targeting. The GPI-anchor transamidase GPI8 was classified as GPI-AP + , but manually rejected due to its function and how its detection was related to the protein sequence from the *Plasmodium* subgenus isolates. Carbonic anhydrase was the only enzyme classified as GPI-AP + among the species in the *Plasmodium* and *Vinckeia* subgenus.

### Multigene families of the GPI-proteome

The composition of the GPI-proteome of 46 Haemosporida isolates was confirmed with the help of the OrthoMCL groups of orthologs. All GPI-AP + candidates also present in *P. falciparum* and *P. vivax* reference sets were conserved at the level of subgenus or throughout the order. Feature description and genomic information were used to investigate the absence of GPI-AP orthologs in some species. About half of the GPI-proteome consists of members of multigene families resulting either from more ancient or more recent duplication events. The two ookinete surface proteins are part of the same group OG6_104197. Genes were located at the same locus and well conserved in most species. A rearrangement was observed in *P. cynomolgi* and *P. vivax* Sal-1 genomes. Fourteen genes are annotated as 6-cystein proteins or putative 6-cystein proteins in *P. falciparum* 3D7 [65]. Seven of these were found to be GPI-AP. Their expression is tightly regulated in *P. falciparum* 3D7. Several of these proteins are expressed on the surface of gametes [65]. The P36 and P52 paralogs are the results of a local duplication that was absent in *H. piliocolobus*. The P36 protein was not GPI-AP in the Haemosporida, but the gene was duplicated in some species of the *Plasmodium* subgenus. This new paralog encodes a GPI-AP 6-cystein protein such as PVX_001015 (while PVX_001025 original P36 remains not GPI-AP, see Table 1). This second duplication event occurred in *Haemamoeba* parasites as well. Interestingly, it was only partial in *P. chabaudi* and did not result in an additional GPI-AP. The duplication was not found in a parasite of the Vinckeia subgenus. OrthoMCL OG6_160720 is the largest group of 6-cystein orthologs. They are located at four different loci, two of which encode the GPI-AP P12 and P58 proteins. A P12 paralog P12p emerged after duplication from an *Hepatocystis piliocolobus* ancestor. The proteins are shorter in *Plasmodium* parasites. The shortest form of the proteins found in *P. cynomolgi* strain B and *Plasmodium malariae* UG01 was not GPI-AP. The two paralogs P47 and P48/45 (OG6_215372) are expressed in tandem in *Plasmodium* genomes. This locus is remarkably well conserved, except in *Plasmodium knowlesi,* where a large insertion between the two genes was observed. The insertion was also present in *P. vivax* Pl01 as well, but to a lesser extent. The insertion events did not appear to be material to the structure or function of the encoded proteins. Evidently, only a few isolates had a P47 ortholog with predictable N-terminal domain (Additional file 8: Table S8, Additional file 9: Table S9).

The merozoite surface proteins MSP1, MSP2, MSP4, MSP5, MSP8, and MSP10 are variable antigens containing one or two copies of an epidermal growth factor (EGF)-like domain and are anchored to the membrane via GPI. The present analysis provides more details regarding their evolution within the order Haemosporida. The merozoite surface protein 1 (MSP1) plays an essential role during the erythrocytic life cycle and is a major malaria vaccine candidate [31, 66, 67]. It is expressed as a large precursor that is processed into four subunits by specific proteases. Furthermore, MSP1 is the most abundant protein at the surface of the parasite. The MSP1p gene emerged as a paralog of MSP1 after duplication at the locus in parasites from the *Haemamoeba* and *Plasmodium* subgenus. The locus was not present in *H. pilicolobus*. In *P. gaboni* SY75 and *P. knowlesi* strain H and Pk1A, MSP1 is classified as GPI-AP- by FT-GPI with the PLA001 and PLA030 parameter combinations (Additional file 8: Table S8, Additional file 9: Table S9). The *P. knowlesi* protein was classified as GPI-AP- by all FT-GPI parameter combinations. The *P. gaboni* protein lacked the C-terminal TM and classified as GPI-AP- by PredGPI. *P. gaboni* is believed to have split from the *P. falciparum*/*P. reichenowi* lineage over 21 million years ago [68]. It is not known whether this variation is due to a specific evolution of the protein or an error in genome sequencing and/or annotation.

MSP2, 4 and 5 are adjacent genes and are tandemly expressed on chromosome 2. Striking similarities among their structural features suggest that they arose from a genetic duplication event [69]. In fact, only the msp4 gene is present in *H. piliocolobus*. In the rodent parasites *P. yoelii* and *P. berghei* only one gene is detectable, and it appears to be homologous to MSP4 and MSP5 [70]. The present analysis confirmed that this organization was in fact associated with subgenus *Vinckeia*. The sequence diversity of MSP4 is reported to be greater than MSP5 [71]. This was confirmed by the number of OrthoMCL groups associated with each protein (Additional file 8: Table S8, Additional file 9: Table S9). *Plasmodium coatneyi*, *Plasmodium fragile* and *Plasmodium inui* msp4 genes could not be identified. *Plasmodium knowlesi* MSP4 proteins were only 201 aa long in both isolates. FT-GPI classified *Plasmodium vivax* Pvl01 ortholog as GPI-AP + only when using PLA000 (the default parameter combination). Otherwise, MSP-5 was shown to have no sequence variation between *P. falciparum* isolates [72]. However, FT-GPI classifies all of the *P. falciparum* 3D7 proteins as GPT-AI-, potentially because all *P. falciparum* MSP5 proteins except 3D7 lack an SP. The protein reference sequence of PF3D7_0206900 is 272 aa long, while others are 261 aa long. Two transcripts have been described in Pf3D7. The presence of an intron overlapping the N-terminus of the coding region leads to the expression of the shortest and GPI-ignoring isoform of MSP5. Further investigation of the protein’s specific location is required [73]. The gene models of *P. coatneyi* Hackeri and *P. fragile* need to be reevaluated on the basis of the different annotations generated for their orthologs. *Plasmodium inui* SA1 lacks an msp5 gene. While GPI anchoring of MSP2 is found in the subgenus Laverania and in the avian parasite *P. gallinaceum* and *P. relictum*, the genomes of rodent parasites and pathogenic parasites of humans and/or non-human primates of the *Plasmodium* subgenus do not encode this protein. OrthoMCL group OG6_533307 encompassed most of the MSP10 proteins. The group included PCOAH_00034770 from *P. coatneyi* Hackeri and YYE_02513 from *P. vinckei* strain vinckei that FT-GPI classified as GP-API- using PLA001 and PLA030. YYE_02513 was classified as GPI-AP + using PLA013 through PLA029 (Additional file 3: Table S3). MSP10 orthologs from isolates of *Plasmodium* subgenus were associated with several OrthoMCL groups confirming previous observations and greater evolutionary distance between GPI-AP in this group of parasites. Interestingly, MSP10 protein orthologs found in *P. cynomolgi* strain and *P. vivax* P01 and Sal-1 strains are members of the OG6_100908 OrthoMCL group, which also includes paralogs of RAMA and FT-GPI3 putative GPI-APs. MSP8 distribution in a single OrthoMCL group is consistent with previous reports that the gene is subject to evolutionary pressures[74].

### GPI-AP specific proteins

The GPI-APs ASP, CSP, GAMA, MiGS, RAMA, P113, P32, P34, PIMMS43 and OG6_533077 (gpi4) have orthologs in most isolates/species. With few exceptions, no paralogs were found. Notably, the gene encoding PIMMS43 was duplicated 20 kbp away from the initial copy on the same chromosome in the *P. knowlesi* genome. The microgamete surface protein MiGS is not currently considered a GPI-AP by other analyses. MiGS of the subgenera *Plasmodium* and *Vinckeia* were characterized by the presence of the Pfam domain PF00026 aspartyl protease. Pfam annotation was absent from proteins of the subgenus *Laverania,* suggesting a specific evolution of the sequence. Knockout of MiGS in *P. yoelii* impaired exflagellation of male gametes [75]. Further research is needed to determine whether this function is related to enzymatic activity in *P. yoelii* and other species. It would be the second enzymatic activity described in *Plasmodium* GPI-AP after the CA presented here. ASP, CSP, GAMA, RAMA and P34 have orthologs in different OrthoMCL groups. Present analysis indicated that a high number of groups is related to sequence diversity and evolution. The highest level of sequence variability was observed for the CSP circumsporozoite protein (Additional file 8: Table S8, Additional file 9: Table S9). Four CSP OrthoMCL groups were associated with *P. falciparum* isolates, which could be surprising as this protein is a major component of the RTS,S vaccine [30]. CSP and MSP2 were the only GPI-AP candidates that showed sequence variation in the *Laverania* subgenus according to OrthoMCL. The present study indicates that several GPI-APs are conserved in the human malaria parasites *P. falciparum*, *P. knowlesi*, *P. malariae*, *Plasmodium ovale* and *Plasmodium vivax*. These parasites have specific modes of interaction with the human host and result in unique clinical presentations, but the identification of a common target for diagnosis, prophylaxis or therapy could be of great interest in the fight against malaria. Some gene models of this group of conserved GPI-APs may need to be reconsidered.

Some *Plasmodium* GPI-APs characterized in the present study are subgenus-specific. For example, CA and FT-GPI1 indicate different relationships between speciation and the evolution of the GPI-proteome. CA was absent from the subgenus *Plasmodium* (Additional file 8: Table S8, Additional file 9: Table S9). Synteny analysis showed that the gene was lost in *P. knowlesi* and *P. vivax*, but the locus was characterized as a pseudogene in *P. cynomolgi* and *P. ovale*. This analysis suggests that the locus alteration may have originated from a common ancestor between *P. malariae,* which still express CA and other members of the subgenus. The FT-GPI1 locus is conserved in the *Plasmodium* species of the Haemosporida order, but proteins are present in three different forms. The longer form (980–1000 aa), classified as GPI-AP + by FT-GPI configured with PLA001 and PLA030, is present in 27 isolates. A form with (780–900 aa) was classified as GPI-AP + by FT-GPI configured with PLA009 through PLA015 (Additional file 3: Table S3). These four proteins were found in the isolates of *P. berghei*, *P. chabaudi* and *Plasmodium vinckei*. They were also classified as GPI-AP + by PredGPI and SignalP. The third group was classified as GPI-AP- by FT-GPI configured with all settings, as well as by other techniques, evidently due to the absence of the N-terminal ER-targeting signal and modification of the C-terminal end. A hydrophobic region was present at the C-terminus but still resulted in GPI-AP- classification by PredGPI. These results suggest that these proteins may be attached to the plasma membrane with a TM.

Analysis of certain GPI-AP candidates by FT-GPI and other techniques raised the possibility of specific sequence and/or annotation errors in the published genomes. This also applies to other GPI-AP candidates. The fpbA-domain FT-GPI2 protein was detected in a considerable number of *P. falciparum* isolates (Additional file 8: Table S8, Additional file 9: Table S9). It is a member of the OG6_146176 group with conserved orthologs in all isolates included in the present study. Candidates from this group classified as GPI-AP + by FT-GPI were also classified as GPI-AP + by PredGPI. One difference between these and other *P. falciparum* isolates is an A insertion in a polyA region, leading to a frameshift and the loss of the terminal hydrophobic domain. FT-GPI3 is part of an OrthoMCL group sharing orthologs at loci located on different chromosomes and encoding the MSP10 or SRA proteins. It is associated with PF3D7_1013800 gene in *P. falciparum* 3D7 and PVX_094905 in *P. vivax* Sal-1. The gene model of FT-GPI3 showed a sequence variation and modification of intron splicing that influenced whether the protein was GPI-AP or not. The gene model organization was generally conserved between taxonomically-close isolates. The gene encoding FT-GPI4 was located in a subtelomeric region of Laverania genomes. FT-GPI configured with PLA001 was more sensitive than PLA030 when classifying FT-GPI4. FT-GPI configured with PLA014 to PLA016 classified all FT-GPI4 proteins as GPI-AP + . Finally, FT-GPI classifies P32 (gpi3) as GPI-AP- in *Laverania* parasites, but PredGPI classifies it as GPI-AP + . This positive prediction by FT-GPI was conserved for most orthologs (OG6_140578). In fact, FT-GPI positive orthologs presented a signal peptide absent from other proteins. The last group of candidates classified as GPI-AP + by FT-GPI included EMAP1 proteins, but only in Vinckeia parasites. EMAP1 is a large group of fam-a proteins [76]. The 829 orthologs from OG6_100943 OrthoMCL group are encoded by *Vinckeia* genome. No experimental evidence exists from the literature for the emergence of GPI-AP from a large multigene family even though a similar prediction among the Rifin and hyp multigene families of the *Laverania* species could be made.

## Conclusions

The present study on the GPI-proteome of Haemosporida parasites was performed at the level of the entire order. The FT-GPI software validation came from its classification of more than a thousand proteins as likely to be GPI-AP + or – and comparing the results with the predictions of other models, as well as reference lists. This approach has shed new light on the evolution of the GPI proteome among *Plasmodium* species. This work and these results were made possible in large part thanks to the continuous effort of the genome sequencing community, and to the community building and maintaining the PlasmoDB database. Both groups have contributed remarkably valuable work by interfacing and making available their detailed data sets. The descriptive approach adopted with FT-GPI, wherein its parameters display a meaningful ontology with our current understanding of biological functions, has helped classify proteins as GPI anchoring. FT-GPI's predictions are based on subtle differences in the positioning of hydrophobic sequences associated with the signal peptide and GPI-anchoring region. This approach enables insightful classification of the GPI-anchoring properties of even those proteins whose encoding genes have evolved sufficiently to evade detection by software trained on amino acid sequence composition.

## Supplementary Information

**Additional file 1: Table S1.** Downloaded proteomes from PlasmoDB database. Amino acid sequences were automatically submitted to FT-GPI analysis.

**Additional file 2: Table S2.** FT-GPI parameter sets. A. FT-GPI combination of FT-GPI parameters values used form the analysis of *Plasmodium* proteomes. Seven parameters were set according to FT-GPI and TMPred options available in the FT-GPI software. B. FT-GPI software options. Parameters are available as options of the software. Colours establish the correspondance between A and B tables. Green corresponds to the options fixing the N-terminal TM parameters. Blue indicates C-terminal TM detection parameters corresponding options. Pink indicates the central TM score and brown shows TMpred TM specific parameters.

**Additional file 3: Table S3.** Taxonomic description of 46 isolates from the Haemosporida order. The proteomes of 46 isolates distributed over the four major subgenera of *Plasmodium* species and one Hepatocystis parasite were downloaded from PlasmoDB database. Colours indicate the main taxonomic groups found in the literature. Laverania-Pg differentiated *P. gaboni* and close species from the *P. falciparum*/*P*. *reichenowi* group of parasites [68]. Human-infecting parasites are in bold. PlasmoDB and other source of information were used to establish the host and geographic origin of the different isolates. *P. faciparum* 3D7 is a reference laboratory strain and first described GPI-proteome in apicomplexa [17]. Cloned: genome sequence was obtained from long-term, established laboratory strains that often pass through a purification step. All *P. falciparum* isolates are of clinical origin. Clinical: non-*P. falciparum* isolates that were recovered from malaria patients

**Additional file 4: Table S4.** Description of proteins from the GPI-AP reference sets. A. *P. falciparum* reference set from Gilson, *et al.* [17]. Protein size, number of TM and signal 4.0 prediction were recovered from PlasmoDB. SignalP 5.0 and PredGPI were performed online [47,52]. Essential genes were described by a Mutation Index Score (MIS) and Fitness score (MFS) from transposon saturation mutagenesis experiments [77]. Sequences were downloaded from PlasmoDB release 51. B. *P. vivax* reference set from Carlton *et al* [38]. The list of GPI-APs was given in Suppl. Table 8 of the *P. vivax* genome paper. Sequence analysis was performed as in (A). No information was available concerning gene essentialness in *P. vivax*. Negative predictions are in red. * Some proteins had no gene names and were arbitrarily labelled from gpi1 to gpi4. *P. falciparum* Pf_gpi1 *P. vivax* ortholog was absent from Carlton *et al* list [38]. *P. falciparum* P36 ortholog was not in Gilson et al list [17]. The gpi3 protein was labelled as P32 in some references.

**Additional file 5: Table S5.** TMPred analysis of GPI-AP reference proteins. TMPred provided three parameters for each predicted TM: the first and last coordinates as well as a score. Data are presented for the first and last TM, called N-term and C-term TM respectively. The middle of the table provided information for all TM predicted in between. The distance to end parameter of the last TM was computed as the difference between the protein length and last coordinate. Proteins in bold indicate those which were positively detected by TMpred for the presence of two TM at both ends of the protein, by PredGPI for being a GPI-AP and by SignalP for the presence of a signal peptide. Missing TM and outliers with extreme TM values are in red. A. *P. falciparum* GPI-APs reference set. Genes with no *P. vivax* orthologs were in italic. B. *P. vivax* GPI-APs reference set. Genes with no *P. falciparum* orthologs are in italic.

**Additional file 6: Table S6.** FT-GPI parameter set efficacy. The Positive and Negative prediction of each combination of FT-GPI parameters was evaluated based on the detection of the 30 reference GPI-APs of *P. falciparum* and *P. vivax* in combination with cytosolic, membrane or among the full proteome. Cytosolic proteins had no TM and signal peptide according to PlasmoDB data (Suppl. Table 4). Membrane proteins presented more than 3 TM according to the same source of information. The formula used to compute the parameters of the test are given with the title of each subsection of the Table. AUC was computed using the binary output of FT-GPI (1 for positive detection and 0 if not).

**Additional file 7: Table S7.** GPI-APs detection using different FT-GPI settings in 46 Haemosporida isolates and comparison with orthologs of the *P. falciparum* 3D7 reference set from Gilson et al [17]. The 31 combination of FT-GPI parameters are described in Suppl. Table 2. Orthologs were obtained from OrthoMCL analysis at PlasmoDB. Efficacy of the FT-GPI set of parameters was computed using the formula described in Suppl. Table 8.

**Additional file 8: Table S8.** Composition of the GPI-proteome of 46 Haemosporida isolates according to FT-GPI PLA001 parameter set. Proteomes were analysed using FT-GPI software. The OrthoMCL annotation (cog_id) and synteny available at PlasmoDB were used to assess the presence of genes in the different isolates. Our analysis was based on proteins presenting more that 210 amino acids. Colour coding was used to determine OrthoMCL group encoding orthologs. The table refers to the number of paralogs found in each genome. Lines merging information from the different orthoMCL groups are marked as Summary in column G. The left part of the table displays the number of orthologs present in PlasmoDB, the FT-GPI detection with the 31 combinations of FT-GPI parameters and the specific detection of PLA001 and PLA030. The GPI-AP provided gene names of the 33 GPI-AP candidates validated in the present study. Four were new GPI-AP compared with *P. falciparum* 3D7 and *P. vivax* Sal-1 GPI-proteome (Fig. 4). SRA detection as GPI-AP was species specific. The sum of validated GPI-AP is given at the top of the table as well as the not-validated putative GPI-AP (from line 121 to 174). Some orthologs with size below 210 aa were introduced in the analysis and are in italics. *P. falciparum* 3D7 and *P. vivax* Sal-1 columns are in bold. The right part of the table provides information about mean, median and standard deviation of the size of protein sequences in recorded per line. * indicates the duplicated OrthoMCL IDs encompassing genes from different loci.

**Additional file 9: Table S9.** Composition of the GPI-proteome of 46 Haemosporida isolates according to FT-GPI PLA030 parameter set. Proteomes were analysed using FT-GPI software. OrthoMCL annotation (cog_id) and synteny available at PlasmoDB were used to assess the presence of genes in the different isolates. Analysis was based on protein presenting more than 210 amino acids. Colour coding was used to determine OrthoMCL group encoding orthologs. The table refers to the number of paralogs found in each genome. Lines merging information from the different orthoMCL groups are marked as summary in column G. The left part of the table displays the number of orthologs present in PlasmoDB, FT-GPI detection with the 31 combinations of FT-GPI parameters and specific detection of PLA001 and PLA030. GPI-AP provided gene names of the 33 GPI-AP candidates validated in the present study. Four were new GPI-AP compared with *P. falciparum* 3D7 and *P. vivax* Sal-1 GPI-proteome (Fig. 4). SRA detection as GPI-AP was species specific. The sum of validated GPI-AP is given at the top of the table as well as the sum not-validated putative GPI-AP (from line 121 to 177). Some orthologs with size below 210 aa were introduced in the analysis and are in italics. *P. falciparum* 3D7 and *P. vivax* Sal-1 columns are in bold. The right part of the table provides information about mean, median and standard deviation of the size of protein sequences in recorded per line. * indicates the duplicated OrthoMCL IDs encompassing genes from different loci.

**Additional file 10: Fig. S1. **Size distribution of GPI-AP present in UNIPROT database. The 29,901 proteins recovered using a query (keyword:KW-0336) were distributed among 2,136 organisms. **A.** Distribution of the log10 of GPI-AP protein sizes. The graph was generated with ggplot. The colour gradients distinguish organisms within each vertical bar. **B.** Distribution density based on the hypothesis that the histogram in (A) is a mixture of two normal distributions. Functions were obtained using normalmixEM function from the mixtools library in R. Similar results were obtained using the Mclust function (not shown). **Fig. S2.** Detection of orthologs of the *P. falciparum* 3D7 reference set from Gilson, et al. [17] using FT-GPI with varying parameter combinations (miniature vertical axes) in 46 Haemosporida isolates. These 31 FT-GPI parameter sets are described in Suppl. Table S2. The horizontal bars depict the number of GPI-AP orthologs detected by each setting of FT-GPI in each isolate. Bars colours are arbitrary. **Fig. S3.** Evolution of the GPI-Proteome among Haemosprida using PLA001 FT-GPI parameters set. Gene encoding proteins with size over 210 aa were selected for this analysis. A. Heatmap representing the distribution of genes among species. The presence of orthologs and paralogs was established using OrthoMCL annotation. Only orthology groups presenting orthologs in more than four species were included in the present analysis. Presence of paralogs were detected for some genes and represented by the red colour scale. A GPI-AP was absent (white) either because it was not detected by PLA001 or the gene was not present in the genome. Some genes were represented by more than one OrthoMCL group. The discrepancy between synteny and orthology groups was due to rapid sequence evolution and shared homologies. Complete species name is given in given in B and suppl Table 3. Laverania-Pg differentiated *P. gaboni* and close species from the *P. falciparum*/*P*. *reichenowi* group of parasites [68]. B. Evolution of the GPI-proteome is related to speciation. The genes were the same as in A. The number of paralogs was set to 1 to compute Jaccard distance. The Ward-2 method was used to build the tree.

## Data Availability

FT-GPI software is available to the scientific community on the https://github.com/rcanovas/FT-GPI.

## Abbreviations

CA: Carbonic anhydrase
CSP: Circumsporozoite protein
ER: Endoplasmic reticulum
GlcN: Glucosamine
GPI: Glycosylphosphatidylinositol
GPI-AP(s): GPI-associated protein(s)
MSP: Merozoite surface multigene
SP: Signal peptide
SVM: Support vector machine
TM: Transmembrane domain
