# Supplementary material for: FT-GPI, a highly sensitive and accurate predictor of GPI-anchored proteins, reveals the composition and evolution of the GPI proteome in Plasmodium species
Source: Malar J. 2023 Jan 25;22:27. doi: 10.1186/s12936-022-04430-0 (PMC9876418; doi:10.1186/s12936-022-04430-0)
Supplement: Supplementary file 7 — Additional file 7: Table S7. GPI-APs detection using different FT-GPI settings in 46 Haemosporida isolates and comparison with orthologs of the P. falciparum 3D7 reference set from Gilson et al [17]. The 31 combination of FT-GPI parameters are described in Suppl. Table 2. Orthologs were obtained from OrthoMCL analysis at PlasmoDB. Efficacy of the FT-GPI set of parameters was computed using the formula described in Suppl. Table 8. [file 12936_2022_4430_MOESM7_ESM.pdf]

Plasmodium fragile strain nilgiri

	Total	TP	FP	TN	FN	Sensitivity	Speficity	Recall	Precision	Error rate	AUC
PLA000	29	6	23	23	18	0.25	0.5	0.5	0.206896551724138	0.585714285714286	0.310714285714286
PLA001	30	13	17	22	14	0.481481481481481	0.564102564102564	0.435897435897436	0.433333333333333	0.46969696969697	0.46547619047619
PLA002	30	14	16	22	13	0.518518518518518	0.578947368421053	0.421052631578947	0.466666666666667	0.446153846153846	0.500595238095238
PLA003	27	12	15	25	15	0.444444444444444	0.625	0.375	0.444444444444444	0.447761194029851	0.473214285714286
PLA004	30	14	16	22	13	0.518518518518518	0.578947368421053	0.421052631578947	0.466666666666667	0.446153846153846	0.500595238095238
PLA005	27	12	15	25	15	0.444444444444444	0.625	0.375	0.444444444444444	0.447761194029851	0.473214285714286
PLA006	30	14	16	22	13	0.518518518518518	0.578947368421053	0.421052631578947	0.466666666666667	0.446153846153846	0.500595238095238
PLA007	30	13	17	22	14	0.481481481481481	0.564102564102564	0.435897435897436	0.433333333333333	0.46969696969697	0.46547619047619
PLA008	30	14	16	22	13	0.518518518518518	0.578947368421053	0.421052631578947	0.466666666666667	0.446153846153846	0.500595238095238
PLA009	36	14	22	16	13	0.518518518518518	0.421052631578947	0.578947368421053	0.388888888888889	0.538461538461538	0.414880952380952
PLA010	35	14	21	17	13	0.518518518518518	0.447368421052632	0.552631578947368	0.4	0.523076923076923	0.429166666666667
PLA011	38	15	23	14	12	0.555555555555556	0.378378378378378	0.621621621621622	0.394736842105263	0.546875	0.421428571428571
PLA012	37	15	22	15	12	0.555555555555556	0.405405405405405	0.594594594594595	0.405405405405405	0.53125	0.435714285714286
PLA013	40	11	29	12	15	0.423076923076923	0.292682926829268	0.707317073170732	0.275	0.656716417910448	0.2875
PLA014	40	11	29	12	15	0.423076923076923	0.292682926829268	0.707317073170732	0.275	0.656716417910448	0.2875
PLA015	40	11	29	12	15	0.423076923076923	0.292682926829268	0.707317073170732	0.275	0.656716417910448	0.2875
PLA016	39	11	28	13	15	0.423076923076923	0.317073170731707	0.682926829268293	0.282051282051282	0.641791044776119	0.301785714285714
PLA017	28	11	17	24	15	0.423076923076923	0.585365853658537	0.414634146341463	0.392857142857143	0.477611940298507	0.444642857142857
PLA018	32	11	21	20	15	0.423076923076923	0.48780487804878	0.51219512195122	0.34375	0.537313432835821	0.3875
PLA019	33	13	20	19	14	0.481481481481481	0.487179487179487	0.512820512820513	0.393939393939394	0.515151515151515	0.422619047619048
PLA020	33	13	20	19	14	0.481481481481481	0.487179487179487	0.512820512820513	0.393939393939394	0.515151515151515	0.422619047619048
PLA021	33	11	22	19	15	0.423076923076923	0.463414634146341	0.536585365853659	0.333333333333333	0.552238805970149	0.373214285714286
PLA022	33	11	22	19	15	0.423076923076923	0.463414634146341	0.536585365853659	0.333333333333333	0.552238805970149	0.373214285714286
PLA023	39	11	28	13	15	0.423076923076923	0.317073170731707	0.682926829268293	0.282051282051282	0.641791044776119	0.301785714285714
PLA024	37	11	26	15	15	0.423076923076923	0.365853658536585	0.634146341463415	0.297297297297297	0.611940298507463	0.330357142857143
PLA025	41	14	27	11	13	0.518518518518518	0.289473684210526	0.710526315789474	0.341463414634146	0.615384615384615	0.357738095238095
PLA026	39	14	25	13	13	0.518518518518518	0.342105263157895	0.657894736842105	0.358974358974359	0.584615384615385	0.386309523809524
PLA027	34	14	20	18	13	0.518518518518518	0.473684210526316	0.526315789473684	0.411764705882353	0.507692307692308	0.443452380952381
PLA028	32	14	18	20	13	0.518518518518518	0.526315789473684	0.473684210526316	0.4375	0.476923076923077	0.47202380952381
PLA029	30	14	16	22	13	0.518518518518518	0.578947368421053	0.421052631578947	0.466666666666667	0.446153846153846	0.500595238095238
PLA030	29	14	15	23	13	0.518518518518518	0.605263157894737	0.394736842105263	0.482758620689655	0.430769230769231	0.514880952380952

Plasmodium inui San Antonio 1

	Total	TP	FP	TN	FN	Sensitivity	Speficity	Recall	Precision	Error rate	AUC
PLA000	31	8	23	21	17	0.32	0.477272727272727	0.522727272727273	0.258064516129032	0.579710144927536	0.3125
PLA001	27	14	13	25	11	0.56	0.657894736842105	0.342105263157895	0.518518518518518	0.380952380952381	0.604166666666667
PLA002	29	14	15	23	11	0.56	0.605263157894737	0.394736842105263	0.482758620689655	0.412698412698413	0.589015151515151
PLA003	29	14	15	23	11	0.56	0.605263157894737	0.394736842105263	0.482758620689655	0.412698412698413	0.573863636363636
PLA004	31	14	17	21	11	0.56	0.552631578947368	0.447368421052632	0.451612903225806	0.444444444444444	0.558712121212121
PLA005	29	14	15	23	11	0.56	0.605263157894737	0.394736842105263	0.482758620689655	0.412698412698413	0.573863636363636
PLA006	31	14	17	21	11	0.56	0.552631578947368	0.447368421052632	0.451612903225806	0.444444444444444	0.558712121212121
PLA007	27	14	13	25	11	0.56	0.657894736842105	0.342105263157895	0.518518518518518	0.380952380952381	0.604166666666667
PLA008	29	14	15	23	11	0.56	0.605263157894737	0.394736842105263	0.482758620689655	0.412698412698413	0.589015151515151
PLA009	33	14	19	19	11	0.56	0.5	0.5	0.424242424242424	0.476190476190476	0.528409090909091
PLA010	33	14	19	19	11	0.56	0.5	0.5	0.424242424242424	0.476190476190476	0.528409090909091
PLA011	36	15	21	16	10	0.6	0.432432432432432	0.567567567567568	0.416666666666667	0.5	0.518939393939394
PLA012	36	15	21	16	10	0.6	0.432432432432432	0.567567567567568	0.416666666666667	0.5	0.518939393939394
PLA013	39	14	25	13	11	0.56	0.342105263157895	0.657894736842105	0.358974358974359	0.571428571428571	0.452651515151515
PLA014	40	14	26	12	11	0.56	0.315789473684211	0.684210526315789	0.35	0.587301587301587	0.4375
PLA015	39	14	25	13	11	0.56	0.342105263157895	0.657894736842105	0.358974358974359	0.571428571428571	0.452651515151515
PLA016	39	14	25	13	11	0.56	0.342105263157895	0.657894736842105	0.358974358974359	0.571428571428571	0.452651515151515
PLA017	27	14	13	25	11	0.56	0.657894736842105	0.342105263157895	0.518518518518518	0.380952380952381	0.619318181818182
PLA018	31	14	17	21	11	0.56	0.552631578947368	0.447368421052632	0.451612903225806	0.444444444444444	0.558712121212121
PLA019	31	15	16	21	10	0.6	0.567567567567568	0.432432432432432	0.483870967741935	0.419354838709677	0.59469696969697
PLA020	31	15	16	21	10	0.6	0.567567567567568	0.432432432432432	0.483870967741935	0.419354838709677	0.59469696969697
PLA021	33	14	19	19	11	0.56	0.5	0.5	0.424242424242424	0.476190476190476	0.528409090909091
PLA022	33	14	19	19	11	0.56	0.5	0.5	0.424242424242424	0.476190476190476	0.528409090909091
PLA023	39	14	25	13	11	0.56	0.342105263157895	0.657894736842105	0.358974358974359	0.571428571428571	0.452651515151515
PLA024	39	14	25	13	11	0.56	0.342105263157895	0.657894736842105	0.358974358974359	0.571428571428571	0.452651515151515
PLA025	40	15	25	12	10	0.6	0.324324324324324	0.675675675675676	0.375	0.564516129032258	0.473484848484849
PLA026	36	15	21	16	10	0.6	0.432432432432432	0.567567567567568	0.416666666666667	0.5	0.534090909090909
PLA027	32	15	17	20	10	0.6	0.540540540540541	0.459459459459459	0.46875	0.435483870967742	0.579545454545454
PLA028	30	14	16	22	11	0.56	0.578947368421053	0.421052631578947	0.466666666666667	0.428571428571429	0.573863636363636
PLA029	28	14	14	24	11	0.56	0.631578947368421	0.368421052631579	0.5	0.396825396825397	0.604166666666667
PLA030	30	14	16	22	11	0.56	0.578947368421053	0.421052631578947	0.466666666666667	0.428571428571429	0.573863636363636

Hepatocystis Piliocolobus 2019

	Total	TP	FP	TN	FN	Sensitivity	Speficity	Recall	Precision	Error rate	AUC
PLA000	31	5	26	17	19	0.208333333333333	0.395348837209302	0.604651162790698	0.161290322580645	0.671641791044776	0.284722222222222
PLA001	26	7	19	22	17	0.291666666666667	0.536585365853659	0.463414634146341	0.269230769230769	0.553846153846154	0.395833333333333
PLA002	25	7	18	23	17	0.291666666666667	0.560975609756098	0.439024390243902	0.28	0.538461538461538	0.409722222222222
PLA003	26	8	18	22	16	0.333333333333333	0.55	0.45	0.307692307692308	0.53125	0.430555555555556
PLA004	26	8	18	22	16	0.333333333333333	0.55	0.45	0.307692307692308	0.53125	0.430555555555556
PLA005	26	8	18	22	16	0.333333333333333	0.55	0.45	0.307692307692308	0.53125	0.430555555555556
PLA006	26	8	18	22	16	0.333333333333333	0.55	0.45	0.307692307692308	0.53125	0.430555555555556
PLA007	26	7	19	22	17	0.291666666666667	0.536585365853659	0.463414634146341	0.269230769230769	0.553846153846154	0.395833333333333
PLA008	25	7	18	23	17	0.291666666666667	0.560975609756098	0.439024390243902	0.28	0.538461538461538	0.409722222222222
PLA009	27	7	20	21	17	0.291666666666667	0.51219512195122	0.48780487804878	0.259259259259259	0.569230769230769	0.381944444444444
PLA010	25	7	18	23	17	0.291666666666667	0.560975609756098	0.439024390243902	0.28	0.538461538461538	0.409722222222222
PLA011	29	8	21	19	16	0.333333333333333	0.475	0.525	0.275862068965517	0.578125	0.388888888888889
PLA012	27	8	19	21	16	0.333333333333333	0.525	0.475	0.296296296296296	0.546875	0.416666666666667
PLA013	36	8	28	12	16	0.333333333333333	0.3	0.7	0.222222222222222	0.6875	0.319444444444444
PLA014	36	8	28	12	16	0.333333333333333	0.3	0.7	0.222222222222222	0.6875	0.319444444444444
PLA015	35	8	27	13	16	0.333333333333333	0.325	0.675	0.228571428571429	0.671875	0.333333333333333
PLA016	35	8	27	13	16	0.333333333333333	0.325	0.675	0.228571428571429	0.671875	0.333333333333333
PLA017	24	7	17	24	17	0.291666666666667	0.585365853658537	0.414634146341463	0.291666666666667	0.523076923076923	0.423611111111111
PLA018	24	7	17	24	17	0.291666666666667	0.585365853658537	0.414634146341463	0.291666666666667	0.523076923076923	0.423611111111111
PLA019	24	7	17	24	17	0.291666666666667	0.585365853658537	0.414634146341463	0.291666666666667	0.523076923076923	0.423611111111111
PLA020	24	7	17	24	17	0.291666666666667	0.585365853658537	0.414634146341463	0.291666666666667	0.523076923076923	0.423611111111111
PLA021	26	8	18	22	16	0.333333333333333	0.55	0.45	0.307692307692308	0.53125	0.430555555555556
PLA022	26	8	18	22	16	0.333333333333333	0.55	0.45	0.307692307692308	0.53125	0.430555555555556
PLA023	36	8	28	12	16	0.333333333333333	0.3	0.7	0.222222222222222	0.6875	0.319444444444444
PLA024	34	8	26	14	16	0.333333333333333	0.35	0.65	0.235294117647059	0.65625	0.347222222222222
PLA025	36	8	28	12	16	0.333333333333333	0.3	0.7	0.222222222222222	0.6875	0.319444444444444
PLA026	37	9	28	11	15	0.375	0.282051282051282	0.717948717948718	0.243243243243243	0.682539682539683	0.340277777777778
PLA027	28	8	20	20	16	0.333333333333333	0.5	0.5	0.285714285714286	0.5625	0.402777777777778
PLA028	27	8	19	21	16	0.333333333333333	0.525	0.475	0.296296296296296	0.546875	0.416666666666667
PLA029	26	8	18	22	16	0.333333333333333	0.55	0.45	0.307692307692308	0.53125	0.430555555555556
PLA030	26	8	18	22	16	0.333333333333333	0.55	0.45	0.307692307692308	0.53125	0.430555555555556

Plasmodium adleri G01

	Total	TP	FP	TN	FN	Sensitivity	Speficity	Recall	Precision	Error rate	AUC
PLA000	47	19	28	22	6	0.76	0.44	0.56	0.404255319148936	0.453333333333333	0.523648648648649
PLA001	32	21	11	37	6	0.777777777777778	0.770833333333333	0.229166666666667	0.65625	0.226666666666667	0.726351351351351
PLA002	33	21	12	36	6	0.777777777777778	0.75	0.25	0.636363636363636	0.24	0.712837837837838
PLA003	34	22	12	35	5	0.814814814814815	0.74468085106383	0.25531914893617	0.647058823529412	0.22972972972973	0.733671171171171
PLA004	35	22	13	34	5	0.814814814814815	0.723404255319149	0.276595744680851	0.628571428571429	0.243243243243243	0.720157657657658
PLA005	34	22	12	35	5	0.814814814814815	0.74468085106383	0.25531914893617	0.647058823529412	0.22972972972973	0.733671171171171
PLA006	35	22	13	34	5	0.814814814814815	0.723404255319149	0.276595744680851	0.628571428571429	0.243243243243243	0.720157657657658
PLA007	32	21	11	37	6	0.777777777777778	0.770833333333333	0.229166666666667	0.65625	0.226666666666667	0.726351351351351
PLA008	33	21	12	36	6	0.777777777777778	0.75	0.25	0.636363636363636	0.24	0.712837837837838
PLA009	39	22	17	30	5	0.814814814814815	0.638297872340426	0.361702127659574	0.564102564102564	0.297297297297297	0.666103603603604
PLA010	39	22	17	30	5	0.814814814814815	0.638297872340426	0.361702127659574	0.564102564102564	0.297297297297297	0.666103603603604
PLA011	45	23	22	24	4	0.851851851851852	0.521739130434783	0.478260869565217	0.511111111111111	0.356164383561644	0.619369369369369
PLA012	45	23	22	24	4	0.851851851851852	0.521739130434783	0.478260869565217	0.511111111111111	0.356164383561644	0.619369369369369
PLA013	56	23	33	13	4	0.851851851851852	0.282608695652174	0.717391304347826	0.410714285714286	0.506849315068493	0.551801801801802
PLA014	56	22	34	13	5	0.814814814814815	0.276595744680851	0.723404255319149	0.392857142857143	0.527027027027027	0.517454954954955
PLA015	56	22	34	13	5	0.814814814814815	0.276595744680851	0.723404255319149	0.392857142857143	0.527027027027027	0.517454954954955
PLA016	55	22	33	14	5	0.814814814814815	0.297872340425532	0.702127659574468	0.4	0.513513513513513	0.530968468468468
PLA017	31	20	11	38	7	0.740740740740741	0.775510204081633	0.224489795918367	0.645161290322581	0.236842105263158	0.705518018018018
PLA018	39	21	18	30	6	0.777777777777778	0.625	0.375	0.538461538461538	0.32	0.631756756756757
PLA019	42	22	20	27	5	0.814814814814815	0.574468085106383	0.425531914893617	0.523809523809524	0.337837837837838	0.625563063063063
PLA020	42	22	20	27	5	0.814814814814815	0.574468085106383	0.425531914893617	0.523809523809524	0.337837837837838	0.625563063063063
PLA021	45	22	23	24	5	0.814814814814815	0.51063829787234	0.48936170212766	0.488888888888889	0.378378378378378	0.585022522522523
PLA022	45	22	23	24	5	0.814814814814815	0.51063829787234	0.48936170212766	0.488888888888889	0.378378378378378	0.585022522522523
PLA023	53	22	31	16	5	0.814814814814815	0.340425531914894	0.659574468085106	0.415094339622642	0.486486486486487	0.557995495495495
PLA024	52	22	30	17	5	0.814814814814815	0.361702127659574	0.638297872340426	0.423076923076923	0.472972972972973	0.571509009009009
PLA025	52	22	30	17	5	0.814814814814815	0.361702127659574	0.638297872340426	0.423076923076923	0.472972972972973	0.571509009009009
PLA026	52	23	29	17	4	0.851851851851852	0.369565217391304	0.630434782608696	0.442307692307692	0.452054794520548	0.619369369369369
PLA027	40	22	18	29	5	0.814814814814815	0.617021276595745	0.382978723404255	0.55	0.310810810810811	0.65259009009009
PLA028	35	22	13	34	5	0.814814814814815	0.723404255319149	0.276595744680851	0.628571428571429	0.243243243243243	0.720157657657658
PLA029	33	21	12	36	6	0.777777777777778	0.75	0.25	0.636363636363636	0.24	0.712837837837838
PLA030	35	22	13	34	5	0.814814814814815	0.723404255319149	0.276595744680851	0.628571428571429	0.243243243243243	0.720157657657658

Plasmodium berghei ANKA

	Total	TP	FP	TN	FN	Sensitivity	Speficity	Recall	Precision	Error rate	AUC
PLA000	43	11	32	20	15	0.423076923076923	0.384615384615385	0.615384615384615	0.255813953488372	0.602564102564103	0.343055555555556
PLA001	35	15	20	28	11	0.576923076923077	0.583333333333333	0.416666666666667	0.428571428571429	0.418918918918919	0.559722222222222
PLA002	35	15	20	28	11	0.576923076923077	0.583333333333333	0.416666666666667	0.428571428571429	0.418918918918919	0.559722222222222
PLA003	34	15	19	29	11	0.576923076923077	0.604166666666667	0.395833333333333	0.441176470588235	0.405405405405405	0.570833333333333
PLA004	35	15	20	28	11	0.576923076923077	0.583333333333333	0.416666666666667	0.428571428571429	0.418918918918919	0.559722222222222
PLA005	34	15	19	29	11	0.576923076923077	0.604166666666667	0.395833333333333	0.441176470588235	0.405405405405405	0.570833333333333
PLA006	35	15	20	28	11	0.576923076923077	0.583333333333333	0.416666666666667	0.428571428571429	0.418918918918919	0.559722222222222
PLA007	35	15	20	28	11	0.576923076923077	0.583333333333333	0.416666666666667	0.428571428571429	0.418918918918919	0.559722222222222
PLA008	35	15	20	28	11	0.576923076923077	0.583333333333333	0.416666666666667	0.428571428571429	0.418918918918919	0.559722222222222
PLA009	43	15	28	20	11	0.576923076923077	0.416666666666667	0.583333333333333	0.348837209302326	0.527027027027027	0.470833333333333
PLA010	41	15	26	22	11	0.576923076923077	0.458333333333333	0.541666666666667	0.365853658536585	0.5	0.493055555555556
PLA011	48	16	32	15	10	0.615384615384615	0.319148936170213	0.680851063829787	0.333333333333333	0.575342465753425	0.447222222222222
PLA012	46	16	30	17	10	0.615384615384615	0.361702127659574	0.638297872340426	0.347826086956522	0.547945205479452	0.469444444444444
PLA013	51	16	35	12	10	0.615384615384615	0.25531914893617	0.74468085106383	0.313725490196078	0.616438356164384	0.425
PLA014	50	16	34	13	10	0.615384615384615	0.276595744680851	0.723404255319149	0.32	0.602739726027397	0.436111111111111
PLA015	50	16	34	13	10	0.615384615384615	0.276595744680851	0.723404255319149	0.32	0.602739726027397	0.436111111111111
PLA016	50	16	34	13	10	0.615384615384615	0.276595744680851	0.723404255319149	0.32	0.602739726027397	0.436111111111111
PLA017	34	15	19	29	11	0.576923076923077	0.604166666666667	0.395833333333333	0.441176470588235	0.405405405405405	0.570833333333333
PLA018	40	15	25	23	11	0.576923076923077	0.479166666666667	0.520833333333333	0.375	0.486486486486487	0.504166666666667
PLA019	44	16	28	19	10	0.615384615384615	0.404255319148936	0.595744680851064	0.363636363636364	0.520547945205479	0.491666666666667
PLA020	44	16	28	19	10	0.615384615384615	0.404255319148936	0.595744680851064	0.363636363636364	0.520547945205479	0.491666666666667
PLA021	44	16	28	19	10	0.615384615384615	0.404255319148936	0.595744680851064	0.363636363636364	0.520547945205479	0.491666666666667
PLA022	44	16	28	19	10	0.615384615384615	0.404255319148936	0.595744680851064	0.363636363636364	0.520547945205479	0.491666666666667
PLA023	49	16	33	14	10	0.615384615384615	0.297872340425532	0.702127659574468	0.326530612244898	0.589041095890411	0.447222222222222
PLA024	47	15	32	16	11	0.576923076923077	0.333333333333333	0.666666666666667	0.319148936170213	0.581081081081081	0.4375
PLA025	49	15	34	14	11	0.576923076923077	0.291666666666667	0.708333333333333	0.306122448979592	0.608108108108108	0.415277777777778
PLA026	49	15	34	14	11	0.576923076923077	0.291666666666667	0.708333333333333	0.306122448979592	0.608108108108108	0.415277777777778
PLA027	44	15	29	19	11	0.576923076923077	0.395833333333333	0.604166666666667	0.340909090909091	0.540540540540541	0.459722222222222
PLA028	40	14	26	23	12	0.538461538461538	0.469387755102041	0.530612244897959	0.35	0.506666666666667	0.472222222222222
PLA029	32	14	18	31	12	0.538461538461538	0.63265306122449	0.36734693877551	0.4375	0.4	0.561111111111111
PLA030	35	15	20	28	11	0.576923076923077	0.583333333333333	0.416666666666667	0.428571428571429	0.418918918918919	0.559722222222222

Plasmodium billcollinsi G01

	Total	TP	FP	TN	FN	Sensitivity	Speficity	Recall	Precision	Error rate	AUC
PLA000	40	15	25	19	10	0.6	0.431818181818182	0.568181818181818	0.375	0.507246376811594	0.420698924731183
PLA001	33	22	11	26	4	0.846153846153846	0.702702702702703	0.297297297297297	0.666666666666667	0.238095238095238	0.739247311827957
PLA002	33	22	11	26	4	0.846153846153846	0.702702702702703	0.297297297297297	0.666666666666667	0.238095238095238	0.739247311827957
PLA003	32	21	11	27	4	0.84	0.710526315789474	0.289473684210526	0.65625	0.238095238095238	0.739247311827957
PLA004	33	22	11	26	4	0.846153846153846	0.702702702702703	0.297297297297297	0.666666666666667	0.238095238095238	0.739247311827957
PLA005	32	21	11	27	4	0.84	0.710526315789474	0.289473684210526	0.65625	0.238095238095238	0.739247311827957
PLA006	33	22	11	26	4	0.846153846153846	0.702702702702703	0.297297297297297	0.666666666666667	0.238095238095238	0.739247311827957
PLA007	33	22	11	26	4	0.846153846153846	0.702702702702703	0.297297297297297	0.666666666666667	0.238095238095238	0.739247311827957
PLA008	33	22	11	26	4	0.846153846153846	0.702702702702703	0.297297297297297	0.666666666666667	0.238095238095238	0.739247311827957
PLA009	36	22	14	23	4	0.846153846153846	0.621621621621622	0.378378378378378	0.611111111111111	0.285714285714286	0.690860215053763
PLA010	36	22	14	23	4	0.846153846153846	0.621621621621622	0.378378378378378	0.611111111111111	0.285714285714286	0.690860215053763
PLA011	41	23	18	18	3	0.884615384615385	0.5	0.5	0.560975609756098	0.338709677419355	0.647177419354839
PLA012	41	23	18	18	3	0.884615384615385	0.5	0.5	0.560975609756098	0.338709677419355	0.647177419354839
PLA013	48	23	25	11	3	0.884615384615385	0.305555555555556	0.694444444444444	0.479166666666667	0.451612903225806	0.566532258064516
PLA014	48	22	26	11	4	0.846153846153846	0.297297297297297	0.702702702702703	0.458333333333333	0.476190476190476	0.529569892473118
PLA015	47	22	25	12	4	0.846153846153846	0.324324324324324	0.675675675675676	0.468085106382979	0.46031746031746	0.545698924731183
PLA016	45	22	23	14	4	0.846153846153846	0.378378378378378	0.621621621621622	0.488888888888889	0.428571428571429	0.577956989247312
PLA017	32	21	11	27	5	0.807692307692308	0.710526315789474	0.289473684210526	0.65625	0.25	0.718413978494624
PLA018	34	21	13	25	5	0.807692307692308	0.657894736842105	0.342105263157895	0.617647058823529	0.28125	0.686155913978495
PLA019	38	21	17	21	5	0.807692307692308	0.552631578947368	0.447368421052632	0.552631578947368	0.34375	0.621639784946237
PLA020	38	21	17	21	5	0.807692307692308	0.552631578947368	0.447368421052632	0.552631578947368	0.34375	0.621639784946237
PLA021	39	22	17	20	4	0.846153846153846	0.540540540540541	0.459459459459459	0.564102564102564	0.333333333333333	0.64247311827957
PLA022	39	22	17	20	4	0.846153846153846	0.540540540540541	0.459459459459459	0.564102564102564	0.333333333333333	0.64247311827957
PLA023	46	22	24	13	4	0.846153846153846	0.351351351351351	0.648648648648649	0.478260869565217	0.444444444444444	0.561827956989247
PLA024	44	21	23	15	5	0.807692307692308	0.394736842105263	0.605263157894737	0.477272727272727	0.4375	0.557123655913978
PLA025	44	21	23	15	5	0.807692307692308	0.394736842105263	0.605263157894737	0.477272727272727	0.4375	0.557123655913978
PLA026	40	21	19	19	5	0.807692307692308	0.5	0.5	0.525	0.375	0.621639784946237
PLA027	34	20	14	25	6	0.769230769230769	0.641025641025641	0.358974358974359	0.588235294117647	0.307692307692308	0.649193548387097
PLA028	31	20	11	28	6	0.769230769230769	0.717948717948718	0.282051282051282	0.645161290322581	0.261538461538462	0.69758064516129
PLA029	30	20	10	29	6	0.769230769230769	0.743589743589744	0.256410256410256	0.666666666666667	0.246153846153846	0.713709677419355
PLA030	33	22	11	26	4	0.846153846153846	0.702702702702703	0.297297297297297	0.666666666666667	0.238095238095238	0.739247311827957

Plasmodium blacklocki G01

	Total	TP	FP	TN	FN	Sensitivity	Speficity	Recall	Precision	Error rate	AUC
PLA000	47	16	31	17	9	0.64	0.354166666666667	0.645833333333333	0.340425531914894	0.547945205479452	0.429166666666667
PLA001	30	21	9	34	6	0.777777777777778	0.790697674418605	0.209302325581395	0.7	0.214285714285714	0.725
PLA002	32	21	11	32	6	0.777777777777778	0.744186046511628	0.255813953488372	0.65625	0.242857142857143	0.725
PLA003	29	20	9	35	6	0.769230769230769	0.795454545454545	0.204545454545455	0.689655172413793	0.214285714285714	0.725
PLA004	33	22	11	31	5	0.814814814814815	0.738095238095238	0.261904761904762	0.666666666666667	0.231884057971014	0.745833333333333
PLA005	29	20	9	35	6	0.769230769230769	0.795454545454545	0.204545454545455	0.689655172413793	0.214285714285714	0.725
PLA006	33	22	11	31	5	0.814814814814815	0.738095238095238	0.261904761904762	0.666666666666667	0.231884057971014	0.745833333333333
PLA007	30	21	9	34	6	0.777777777777778	0.790697674418605	0.209302325581395	0.7	0.214285714285714	0.725
PLA008	32	21	11	32	6	0.777777777777778	0.744186046511628	0.255813953488372	0.65625	0.242857142857143	0.725
PLA009	40	22	18	24	5	0.814814814814815	0.571428571428571	0.428571428571429	0.55	0.333333333333333	0.6625
PLA010	40	22	18	24	5	0.814814814814815	0.571428571428571	0.428571428571429	0.55	0.333333333333333	0.6625
PLA011	46	22	24	18	5	0.814814814814815	0.428571428571429	0.571428571428571	0.478260869565217	0.420289855072464	0.6125
PLA012	46	22	24	18	5	0.814814814814815	0.428571428571429	0.571428571428571	0.478260869565217	0.420289855072464	0.6125
PLA013	51	21	30	13	6	0.777777777777778	0.302325581395349	0.697674418604651	0.411764705882353	0.514285714285714	0.508333333333333
PLA014	49	21	28	15	6	0.777777777777778	0.348837209302326	0.651162790697674	0.428571428571429	0.485714285714286	0.508333333333333
PLA015	49	21	28	15	6	0.777777777777778	0.348837209302326	0.651162790697674	0.428571428571429	0.485714285714286	0.508333333333333
PLA016	46	21	25	18	6	0.777777777777778	0.418604651162791	0.581395348837209	0.456521739130435	0.442857142857143	0.558333333333333
PLA017	28	20	8	36	7	0.740740740740741	0.818181818181818	0.181818181818182	0.714285714285714	0.211267605633803	0.720833333333333
PLA018	34	21	13	30	6	0.777777777777778	0.697674418604651	0.302325581395349	0.617647058823529	0.271428571428571	0.691666666666667
PLA019	39	22	17	25	5	0.814814814814815	0.595238095238095	0.404761904761905	0.564102564102564	0.318840579710145	0.679166666666667
PLA020	39	22	17	25	5	0.814814814814815	0.595238095238095	0.404761904761905	0.564102564102564	0.318840579710145	0.679166666666667
PLA021	39	21	18	25	6	0.777777777777778	0.581395348837209	0.418604651162791	0.538461538461538	0.342857142857143	0.641666666666667
PLA022	39	21	18	25	6	0.777777777777778	0.581395348837209	0.418604651162791	0.538461538461538	0.342857142857143	0.641666666666667
PLA023	43	21	22	21	6	0.777777777777778	0.488372093023256	0.511627906976744	0.488372093023256	0.4	0.575
PLA024	41	21	20	23	6	0.777777777777778	0.534883720930233	0.465116279069767	0.51219512195122	0.371428571428571	0.575
PLA025	41	21	20	23	6	0.777777777777778	0.534883720930233	0.465116279069767	0.51219512195122	0.371428571428571	0.575
PLA026	40	22	18	24	5	0.814814814814815	0.571428571428571	0.428571428571429	0.55	0.333333333333333	0.629166666666667
PLA027	34	22	12	30	5	0.814814814814815	0.714285714285714	0.285714285714286	0.647058823529412	0.246376811594203	0.695833333333333
PLA028	29	22	7	35	5	0.814814814814815	0.833333333333333	0.166666666666667	0.758620689655172	0.173913043478261	0.779166666666667
PLA029	28	21	7	36	6	0.777777777777778	0.837209302325581	0.162790697674419	0.75	0.185714285714286	0.758333333333333
PLA030	33	22	11	31	5	0.814814814814815	0.738095238095238	0.261904761904762	0.666666666666667	0.231884057971014	0.745833333333333

Plasmodium chabaudi chabaudi

	Total	TP	FP	TN	FN	Sensitivity	Speficity	Recall	Precision	Error rate	AUC
PLA000	38	13	25	22	13	0.5	0.468085106382979	0.531914893617021	0.342105263157895	0.520547945205479	0.436483739837398
PLA001	32	17	15	28	10	0.62962962962963	0.651162790697674	0.348837209302326	0.53125	0.357142857142857	0.608739837398374
PLA002	33	17	16	27	10	0.62962962962963	0.627906976744186	0.372093023255814	0.515151515151515	0.371428571428571	0.596544715447155
PLA003	30	14	16	30	13	0.518518518518518	0.652173913043478	0.347826086956522	0.466666666666667	0.397260273972603	0.534044715447154
PLA004	32	15	17	28	12	0.555555555555556	0.622222222222222	0.377777777777778	0.46875	0.402777777777778	0.542682926829268
PLA005	30	14	16	30	13	0.518518518518518	0.652173913043478	0.347826086956522	0.466666666666667	0.397260273972603	0.534044715447154
PLA006	32	15	17	28	12	0.555555555555556	0.622222222222222	0.377777777777778	0.46875	0.402777777777778	0.542682926829268
PLA007	32	17	15	28	10	0.62962962962963	0.651162790697674	0.348837209302326	0.53125	0.357142857142857	0.608739837398374
PLA008	33	17	16	27	10	0.62962962962963	0.627906976744186	0.372093023255814	0.515151515151515	0.371428571428571	0.596544715447155
PLA009	41	17	24	19	10	0.62962962962963	0.441860465116279	0.558139534883721	0.414634146341463	0.485714285714286	0.498983739837398
PLA010	40	17	23	20	10	0.62962962962963	0.465116279069767	0.534883720930233	0.425	0.471428571428571	0.511178861788618
PLA011	45	17	28	15	10	0.62962962962963	0.348837209302326	0.651162790697674	0.377777777777778	0.542857142857143	0.46239837398374
PLA012	44	17	27	16	10	0.62962962962963	0.372093023255814	0.627906976744186	0.386363636363636	0.528571428571429	0.474593495934959
PLA013	49	16	33	11	10	0.615384615384615	0.25	0.75	0.326530612244898	0.614285714285714	0.401422764227642
PLA014	52	16	36	8	10	0.615384615384615	0.181818181818182	0.818181818181818	0.307692307692308	0.657142857142857	0.364837398373984
PLA015	50	16	34	10	10	0.615384615384615	0.227272727272727	0.772727272727273	0.32	0.628571428571429	0.389227642276423
PLA016	48	16	32	12	10	0.615384615384615	0.272727272727273	0.727272727272727	0.333333333333333	0.6	0.413617886178862
PLA017	30	16	14	30	10	0.615384615384615	0.681818181818182	0.318181818181818	0.533333333333333	0.342857142857143	0.620934959349594
PLA018	37	16	21	23	10	0.615384615384615	0.522727272727273	0.477272727272727	0.432432432432432	0.442857142857143	0.535569105691057
PLA019	40	16	24	20	11	0.592592592592593	0.454545454545455	0.545454545454545	0.4	0.492957746478873	0.478150406504065
PLA020	40	16	24	20	11	0.592592592592593	0.454545454545455	0.545454545454545	0.4	0.492957746478873	0.478150406504065
PLA021	40	16	24	20	10	0.615384615384615	0.454545454545455	0.545454545454545	0.4	0.485714285714286	0.498983739837398
PLA022	40	16	24	20	10	0.615384615384615	0.454545454545455	0.545454545454545	0.4	0.485714285714286	0.498983739837398
PLA023	48	16	32	12	10	0.615384615384615	0.272727272727273	0.727272727272727	0.333333333333333	0.6	0.413617886178862
PLA024	46	15	31	14	11	0.576923076923077	0.311111111111111	0.688888888888889	0.326086956521739	0.591549295774648	0.404979674796748
PLA025	49	16	33	11	11	0.592592592592593	0.25	0.75	0.326530612244898	0.619718309859155	0.380589430894309
PLA026	48	16	32	12	11	0.592592592592593	0.272727272727273	0.727272727272727	0.333333333333333	0.605633802816901	0.392784552845528
PLA027	40	16	24	20	11	0.592592592592593	0.454545454545455	0.545454545454545	0.4	0.492957746478873	0.478150406504065
PLA028	36	15	21	24	12	0.555555555555556	0.533333333333333	0.466666666666667	0.416666666666667	0.458333333333333	0.49390243902439
PLA029	31	15	16	29	12	0.555555555555556	0.644444444444444	0.355555555555556	0.483870967741935	0.388888888888889	0.554878048780488
PLA030	30	15	15	30	12	0.555555555555556	0.666666666666667	0.333333333333333	0.5	0.375	0.567073170731707

Plasmodium coatneyi Hackeri

	Total	TP	FP	TN	FN	Sensitivity	Speficity	Recall	Precision	Error rate	AUC
PLA000	33	8	25	15	16	0.333333333333333	0.375	0.625	0.242424242424242	0.640625	0.291666666666667
PLA001	22	12	10	26	14	0.461538461538462	0.722222222222222	0.277777777777778	0.545454545454545	0.387096774193548	0.552083333333333
PLA002	24	13	11	24	13	0.5	0.685714285714286	0.314285714285714	0.541666666666667	0.39344262295082	0.557291666666667
PLA003	22	13	9	26	13	0.5	0.742857142857143	0.257142857142857	0.590909090909091	0.360655737704918	0.588541666666667
PLA004	23	13	10	25	13	0.5	0.714285714285714	0.285714285714286	0.565217391304348	0.377049180327869	0.572916666666667
PLA005	22	13	9	26	13	0.5	0.742857142857143	0.257142857142857	0.590909090909091	0.360655737704918	0.588541666666667
PLA006	23	13	10	25	13	0.5	0.714285714285714	0.285714285714286	0.565217391304348	0.377049180327869	0.572916666666667
PLA007	22	12	10	26	14	0.461538461538462	0.722222222222222	0.277777777777778	0.545454545454545	0.387096774193548	0.552083333333333
PLA008	24	13	11	24	13	0.5	0.685714285714286	0.314285714285714	0.541666666666667	0.39344262295082	0.557291666666667
PLA009	28	14	14	20	12	0.538461538461538	0.588235294117647	0.411764705882353	0.5	0.433333333333333	0.53125
PLA010	28	14	14	20	12	0.538461538461538	0.588235294117647	0.411764705882353	0.5	0.433333333333333	0.53125
PLA011	31	15	16	17	11	0.576923076923077	0.515151515151515	0.484848484848485	0.483870967741935	0.457627118644068	0.520833333333333
PLA012	31	15	16	17	11	0.576923076923077	0.515151515151515	0.484848484848485	0.483870967741935	0.457627118644068	0.520833333333333
PLA013	35	14	21	13	12	0.538461538461538	0.382352941176471	0.617647058823529	0.4	0.55	0.4375
PLA014	34	13	21	14	13	0.5	0.4	0.6	0.382352941176471	0.557377049180328	0.416666666666667
PLA015	34	13	21	14	13	0.5	0.4	0.6	0.382352941176471	0.557377049180328	0.416666666666667
PLA016	34	13	21	14	13	0.5	0.4	0.6	0.382352941176471	0.557377049180328	0.416666666666667
PLA017	21	11	10	27	15	0.423076923076923	0.72972972972973	0.27027027027027	0.523809523809524	0.396825396825397	0.53125
PLA018	25	12	13	23	14	0.461538461538462	0.638888888888889	0.361111111111111	0.48	0.435483870967742	0.505208333333333
PLA019	26	11	15	22	15	0.423076923076923	0.594594594594595	0.405405405405405	0.423076923076923	0.476190476190476	0.453125
PLA020	26	11	15	22	15	0.423076923076923	0.594594594594595	0.405405405405405	0.423076923076923	0.476190476190476	0.453125
PLA021	28	13	15	20	13	0.5	0.571428571428571	0.428571428571429	0.464285714285714	0.459016393442623	0.494791666666667
PLA022	28	13	15	20	13	0.5	0.571428571428571	0.428571428571429	0.464285714285714	0.459016393442623	0.494791666666667
PLA023	34	13	21	14	13	0.5	0.4	0.6	0.382352941176471	0.557377049180328	0.416666666666667
PLA024	32	13	19	16	13	0.5	0.457142857142857	0.542857142857143	0.40625	0.524590163934426	0.447916666666667
PLA025	33	14	19	15	12	0.538461538461538	0.441176470588235	0.558823529411765	0.424242424242424	0.516666666666667	0.46875
PLA026	32	14	18	16	12	0.538461538461538	0.470588235294118	0.529411764705882	0.4375	0.5	0.484375
PLA027	25	12	13	23	14	0.461538461538462	0.638888888888889	0.361111111111111	0.48	0.435483870967742	0.505208333333333
PLA028	21	12	9	27	14	0.461538461538462	0.75	0.25	0.571428571428571	0.370967741935484	0.567708333333333
PLA029	18	12	6	30	14	0.461538461538462	0.833333333333333	0.166666666666667	0.666666666666667	0.32258064516129	0.614583333333333
PLA030	21	13	8	27	13	0.5	0.771428571428571	0.228571428571429	0.619047619047619	0.344262295081967	0.604166666666667

Plasmodium cynomolgi strain B

	Total	TP	FP	TN	FN	Sensitivity	Speficity	Recall	Precision	Error rate	AUC
PLA000	30	8	22	12	17	0.32	0.352941176470588	0.647058823529412	0.266666666666667	0.661016949152542	0.283764367816092
PLA001	18	10	8	24	15	0.4	0.75	0.25	0.555555555555556	0.403508771929825	0.566810344827586
PLA002	18	10	8	24	15	0.4	0.75	0.25	0.555555555555556	0.403508771929825	0.566810344827586
PLA003	18	10	8	24	15	0.4	0.75	0.25	0.555555555555556	0.403508771929825	0.566810344827586
PLA004	18	10	8	24	15	0.4	0.75	0.25	0.555555555555556	0.403508771929825	0.566810344827586
PLA005	18	10	8	24	15	0.4	0.75	0.25	0.555555555555556	0.403508771929825	0.566810344827586
PLA006	18	10	8	24	15	0.4	0.75	0.25	0.555555555555556	0.403508771929825	0.566810344827586
PLA007	18	10	8	24	15	0.4	0.75	0.25	0.555555555555556	0.403508771929825	0.566810344827586
PLA008	18	10	8	24	15	0.4	0.75	0.25	0.555555555555556	0.403508771929825	0.566810344827586
PLA009	21	10	11	21	15	0.4	0.65625	0.34375	0.476190476190476	0.456140350877193	0.515086206896552
PLA010	21	10	11	21	15	0.4	0.65625	0.34375	0.476190476190476	0.456140350877193	0.515086206896552
PLA011	25	11	14	17	14	0.44	0.548387096774194	0.451612903225806	0.44	0.5	0.484195402298851
PLA012	25	11	14	17	14	0.44	0.548387096774194	0.451612903225806	0.44	0.5	0.484195402298851
PLA013	34	10	24	8	15	0.4	0.25	0.75	0.294117647058824	0.684210526315789	0.308189655172414
PLA014	34	10	24	8	15	0.4	0.25	0.75	0.294117647058824	0.684210526315789	0.308189655172414
PLA015	32	10	22	10	15	0.4	0.3125	0.6875	0.3125	0.649122807017544	0.342672413793103
PLA016	31	10	21	11	15	0.4	0.34375	0.65625	0.32258064516129	0.631578947368421	0.359913793103448
PLA017	16	9	7	26	16	0.36	0.787878787878788	0.212121212121212	0.5625	0.396551724137931	0.563218390804598
PLA018	19	9	10	23	16	0.36	0.696969696969697	0.303030303030303	0.473684210526316	0.448275862068966	0.511494252873563
PLA019	21	9	12	21	16	0.36	0.636363636363636	0.363636363636364	0.428571428571429	0.482758620689655	0.477011494252874
PLA020	21	9	12	21	16	0.36	0.636363636363636	0.363636363636364	0.428571428571429	0.482758620689655	0.477011494252874
PLA021	22	10	12	20	15	0.4	0.625	0.375	0.454545454545455	0.473684210526316	0.497844827586207
PLA022	22	10	12	20	15	0.4	0.625	0.375	0.454545454545455	0.473684210526316	0.497844827586207
PLA023	32	9	23	10	16	0.36	0.303030303030303	0.696969696969697	0.28125	0.672413793103448	0.304597701149425
PLA024	31	9	22	11	16	0.36	0.333333333333333	0.666666666666667	0.290322580645161	0.655172413793103	0.32183908045977
PLA025	31	9	22	11	16	0.36	0.333333333333333	0.666666666666667	0.290322580645161	0.655172413793103	0.32183908045977
PLA026	24	9	15	18	16	0.36	0.545454545454545	0.454545454545455	0.375	0.53448275862069	0.442528735632184
PLA027	17	8	9	25	17	0.32	0.735294117647059	0.264705882352941	0.470588235294118	0.440677966101695	0.507902298850575
PLA028	15	8	7	27	17	0.32	0.794117647058823	0.205882352941176	0.533333333333333	0.406779661016949	0.542385057471264
PLA029	13	8	5	29	17	0.32	0.852941176470588	0.147058823529412	0.615384615384615	0.372881355932203	0.576867816091954
PLA030	17	10	7	25	15	0.4	0.78125	0.21875	0.588235294117647	0.385964912280702	0.584051724137931

Plasmodium cynomolgi strain M

	Total	TP	FP	TN	FN	Sensitivity	Speficity	Recall	Precision	Error rate	AUC
PLA000	45	14	31	20	13	0.518518518518518	0.392156862745098	0.607843137254902	0.311111111111111	0.564102564102564	0.395833333333333
PLA001	36	19	17	29	9	0.678571428571429	0.630434782608696	0.369565217391304	0.527777777777778	0.351351351351351	0.633928571428571
PLA002	36	19	17	29	9	0.678571428571429	0.630434782608696	0.369565217391304	0.527777777777778	0.351351351351351	0.633928571428571
PLA003	37	19	18	28	9	0.678571428571429	0.608695652173913	0.391304347826087	0.513513513513513	0.364864864864865	0.62202380952381
PLA004	37	19	18	28	9	0.678571428571429	0.608695652173913	0.391304347826087	0.513513513513513	0.364864864864865	0.62202380952381
PLA005	37	19	18	28	9	0.678571428571429	0.608695652173913	0.391304347826087	0.513513513513513	0.364864864864865	0.62202380952381
PLA006	37	19	18	28	9	0.678571428571429	0.608695652173913	0.391304347826087	0.513513513513513	0.364864864864865	0.62202380952381
PLA007	36	19	17	29	9	0.678571428571429	0.630434782608696	0.369565217391304	0.527777777777778	0.351351351351351	0.633928571428571
PLA008	36	19	17	29	9	0.678571428571429	0.630434782608696	0.369565217391304	0.527777777777778	0.351351351351351	0.633928571428571
PLA009	40	19	21	25	9	0.678571428571429	0.543478260869565	0.456521739130435	0.475	0.405405405405405	0.586309523809524
PLA010	40	19	21	25	9	0.678571428571429	0.543478260869565	0.456521739130435	0.475	0.405405405405405	0.586309523809524
PLA011	44	20	24	21	8	0.714285714285714	0.466666666666667	0.533333333333333	0.454545454545455	0.438356164383562	0.571428571428571
PLA012	44	20	24	21	8	0.714285714285714	0.466666666666667	0.533333333333333	0.454545454545455	0.438356164383562	0.571428571428571
PLA013	50	19	31	15	9	0.678571428571429	0.326086956521739	0.673913043478261	0.38	0.540540540540541	0.479166666666667
PLA014	50	19	31	15	9	0.678571428571429	0.326086956521739	0.673913043478261	0.38	0.540540540540541	0.479166666666667
PLA015	48	18	30	17	10	0.642857142857143	0.361702127659574	0.638297872340426	0.375	0.533333333333333	0.470238095238095
PLA016	47	18	29	18	10	0.642857142857143	0.382978723404255	0.617021276595745	0.382978723404255	0.52	0.482142857142857
PLA017	30	17	13	35	11	0.607142857142857	0.729166666666667	0.270833333333333	0.566666666666667	0.315789473684211	0.639880952380952
PLA018	34	17	17	31	11	0.607142857142857	0.645833333333333	0.354166666666667	0.5	0.368421052631579	0.592261904761905
PLA019	36	17	19	29	11	0.607142857142857	0.604166666666667	0.395833333333333	0.472222222222222	0.394736842105263	0.568452380952381
PLA020	36	17	19	29	11	0.607142857142857	0.604166666666667	0.395833333333333	0.472222222222222	0.394736842105263	0.568452380952381
PLA021	38	18	20	27	10	0.642857142857143	0.574468085106383	0.425531914893617	0.473684210526316	0.4	0.577380952380952
PLA022	38	18	20	27	10	0.642857142857143	0.574468085106383	0.425531914893617	0.473684210526316	0.4	0.577380952380952
PLA023	48	18	30	17	10	0.642857142857143	0.361702127659574	0.638297872340426	0.375	0.533333333333333	0.470238095238095
PLA024	46	18	28	19	10	0.642857142857143	0.404255319148936	0.595744680851064	0.391304347826087	0.506666666666667	0.494047619047619
PLA025	48	18	30	17	10	0.642857142857143	0.361702127659574	0.638297872340426	0.375	0.533333333333333	0.470238095238095
PLA026	45	18	27	20	10	0.642857142857143	0.425531914893617	0.574468085106383	0.4	0.493333333333333	0.505952380952381
PLA027	36	17	19	29	11	0.607142857142857	0.604166666666667	0.395833333333333	0.472222222222222	0.394736842105263	0.568452380952381
PLA028	35	17	18	30	11	0.607142857142857	0.625	0.375	0.485714285714286	0.381578947368421	0.580357142857143
PLA029	32	17	15	33	11	0.607142857142857	0.6875	0.3125	0.53125	0.342105263157895	0.616071428571428
PLA030	36	19	17	29	9	0.678571428571429	0.630434782608696	0.369565217391304	0.527777777777778	0.351351351351351	0.633928571428571

Plasmodium falciparum 3D7

	Total	TP	FP	TN	FN	Sensitivity	Speficity	Recall	Precision	Error rate	AUC
PLA000	50	21	29	15	5	0.807692307692308	0.340909090909091	0.659090909090909	0.42	0.485714285714286	0.486742424242424
PLA001	34	24	10	31	3	0.888888888888889	0.75609756097561	0.24390243902439	0.705882352941177	0.191176470588235	0.785984848484849
PLA002	34	24	10	31	3	0.888888888888889	0.75609756097561	0.24390243902439	0.705882352941177	0.191176470588235	0.785984848484849
PLA003	34	24	10	31	3	0.888888888888889	0.75609756097561	0.24390243902439	0.705882352941177	0.191176470588235	0.785984848484849
PLA004	34	24	10	31	3	0.888888888888889	0.75609756097561	0.24390243902439	0.705882352941177	0.191176470588235	0.785984848484849
PLA005	34	24	10	31	3	0.888888888888889	0.75609756097561	0.24390243902439	0.705882352941177	0.191176470588235	0.785984848484849
PLA006	34	24	10	31	3	0.888888888888889	0.75609756097561	0.24390243902439	0.705882352941177	0.191176470588235	0.785984848484849
PLA007	34	24	10	31	3	0.888888888888889	0.75609756097561	0.24390243902439	0.705882352941177	0.191176470588235	0.785984848484849
PLA008	34	24	10	31	3	0.888888888888889	0.75609756097561	0.24390243902439	0.705882352941177	0.191176470588235	0.785984848484849
PLA009	39	25	14	26	2	0.925925925925926	0.65	0.35	0.641025641025641	0.238805970149254	0.761363636363636
PLA010	39	25	14	26	2	0.925925925925926	0.65	0.35	0.641025641025641	0.238805970149254	0.761363636363636
PLA011	44	26	18	21	1	0.962962962962963	0.538461538461538	0.461538461538462	0.590909090909091	0.287878787878788	0.721590909090909
PLA012	44	26	18	21	1	0.962962962962963	0.538461538461538	0.461538461538462	0.590909090909091	0.287878787878788	0.721590909090909
PLA013	49	25	24	16	2	0.925925925925926	0.4	0.6	0.510204081632653	0.388059701492537	0.640151515151515
PLA014	49	24	25	16	3	0.888888888888889	0.390243902439024	0.609756097560976	0.489795918367347	0.411764705882353	0.604166666666667
PLA015	48	24	24	17	3	0.888888888888889	0.414634146341463	0.585365853658537	0.5	0.397058823529412	0.619318181818182
PLA016	47	24	23	18	3	0.888888888888889	0.439024390243902	0.560975609756098	0.51063829787234	0.382352941176471	0.634469696969697
PLA017	31	22	9	34	5	0.814814814814815	0.790697674418605	0.209302325581395	0.709677419354839	0.2	0.759469696969697
PLA018	35	23	12	30	4	0.851851851851852	0.714285714285714	0.285714285714286	0.657142857142857	0.231884057971014	0.75
PLA019	40	24	16	25	3	0.888888888888889	0.609756097560976	0.390243902439024	0.6	0.279411764705882	0.710227272727273
PLA020	40	24	16	25	3	0.888888888888889	0.609756097560976	0.390243902439024	0.6	0.279411764705882	0.710227272727273
PLA021	40	24	16	25	3	0.888888888888889	0.609756097560976	0.390243902439024	0.6	0.279411764705882	0.710227272727273
PLA022	40	24	16	25	3	0.888888888888889	0.609756097560976	0.390243902439024	0.6	0.279411764705882	0.710227272727273
PLA023	45	24	21	20	3	0.888888888888889	0.48780487804878	0.51219512195122	0.533333333333333	0.352941176470588	0.664772727272727
PLA024	43	24	19	22	3	0.888888888888889	0.536585365853659	0.463414634146341	0.558139534883721	0.323529411764706	0.695075757575758
PLA025	44	25	19	21	2	0.925925925925926	0.525	0.475	0.568181818181818	0.313432835820896	0.715909090909091
PLA026	42	25	17	23	2	0.925925925925926	0.575	0.425	0.595238095238095	0.283582089552239	0.731060606060606
PLA027	36	24	12	29	3	0.888888888888889	0.707317073170732	0.292682926829268	0.666666666666667	0.220588235294118	0.770833333333333
PLA028	33	24	9	32	3	0.888888888888889	0.780487804878049	0.219512195121951	0.727272727272727	0.176470588235294	0.816287878787879
PLA029	31	23	8	34	4	0.851851851851852	0.80952380952381	0.19047619047619	0.741935483870968	0.173913043478261	0.795454545454546
PLA030	32	24	8	33	3	0.888888888888889	0.804878048780488	0.195121951219512	0.75	0.161764705882353	0.816287878787879

Plasmodium falciparum 7G8

	Total	TP	FP	TN	FN	Sensitivity	Speficity	Recall	Precision	Error rate	AUC
PLA000	52	18	34	15	8	0.692307692307692	0.306122448979592	0.693877551020408	0.346153846153846	0.56	0.42156862745098
PLA001	31	22	9	36	5	0.814814814814815	0.8	0.2	0.709677419354839	0.194444444444444	0.763480392156863
PLA002	31	22	9	36	5	0.814814814814815	0.8	0.2	0.709677419354839	0.194444444444444	0.763480392156863
PLA003	31	22	9	36	5	0.814814814814815	0.8	0.2	0.709677419354839	0.194444444444444	0.763480392156863
PLA004	31	22	9	36	5	0.814814814814815	0.8	0.2	0.709677419354839	0.194444444444444	0.763480392156863
PLA005	31	22	9	36	5	0.814814814814815	0.8	0.2	0.709677419354839	0.194444444444444	0.763480392156863
PLA006	31	22	9	36	5	0.814814814814815	0.8	0.2	0.709677419354839	0.194444444444444	0.763480392156863
PLA007	31	22	9	36	5	0.814814814814815	0.8	0.2	0.709677419354839	0.194444444444444	0.763480392156863
PLA008	31	22	9	36	5	0.814814814814815	0.8	0.2	0.709677419354839	0.194444444444444	0.763480392156863
PLA009	35	23	12	32	4	0.851851851851852	0.727272727272727	0.272727272727273	0.657142857142857	0.225352112676056	0.754901960784314
PLA010	35	23	12	32	4	0.851851851851852	0.727272727272727	0.272727272727273	0.657142857142857	0.225352112676056	0.754901960784314
PLA011	42	24	18	25	3	0.888888888888889	0.581395348837209	0.418604651162791	0.571428571428571	0.3	0.702205882352941
PLA012	42	24	18	25	3	0.888888888888889	0.581395348837209	0.418604651162791	0.571428571428571	0.3	0.702205882352941
PLA013	48	23	25	19	4	0.851851851851852	0.431818181818182	0.568181818181818	0.479166666666667	0.408450704225352	0.607843137254902
PLA014	49	22	27	18	5	0.814814814814815	0.4	0.6	0.448979591836735	0.444444444444444	0.572303921568627
PLA015	49	22	27	18	5	0.814814814814815	0.4	0.6	0.448979591836735	0.444444444444444	0.572303921568627
PLA016	48	22	26	19	5	0.814814814814815	0.422222222222222	0.577777777777778	0.458333333333333	0.430555555555556	0.587009803921569
PLA017	30	20	10	37	7	0.740740740740741	0.787234042553192	0.212765957446809	0.666666666666667	0.22972972972973	0.707107843137255
PLA018	34	21	13	33	6	0.777777777777778	0.717391304347826	0.282608695652174	0.617647058823529	0.26027397260274	0.698529411764706
PLA019	38	22	16	29	5	0.814814814814815	0.644444444444444	0.355555555555556	0.578947368421053	0.291666666666667	0.675245098039216
PLA020	38	22	16	29	5	0.814814814814815	0.644444444444444	0.355555555555556	0.578947368421053	0.291666666666667	0.675245098039216
PLA021	41	22	19	26	5	0.814814814814815	0.577777777777778	0.422222222222222	0.536585365853659	0.333333333333333	0.645833333333333
PLA022	41	22	19	26	5	0.814814814814815	0.577777777777778	0.422222222222222	0.536585365853659	0.333333333333333	0.645833333333333
PLA023	45	22	23	22	5	0.814814814814815	0.488888888888889	0.511111111111111	0.488888888888889	0.388888888888889	0.631127450980392
PLA024	42	22	20	25	5	0.814814814814815	0.555555555555556	0.444444444444444	0.523809523809524	0.347222222222222	0.660539215686274
PLA025	43	23	20	24	4	0.851851851851852	0.545454545454545	0.454545454545455	0.534883720930233	0.338028169014085	0.681372549019608
PLA026	42	23	19	25	4	0.851851851851852	0.568181818181818	0.431818181818182	0.547619047619048	0.323943661971831	0.681372549019608
PLA027	36	22	14	31	5	0.814814814814815	0.688888888888889	0.311111111111111	0.611111111111111	0.263888888888889	0.704656862745098
PLA028	33	22	11	34	5	0.814814814814815	0.755555555555556	0.244444444444444	0.666666666666667	0.222222222222222	0.748774509803921
PLA029	31	21	10	36	6	0.777777777777778	0.782608695652174	0.217391304347826	0.67741935483871	0.219178082191781	0.727941176470588
PLA030	31	22	9	36	5	0.814814814814815	0.8	0.2	0.709677419354839	0.194444444444444	0.763480392156863

Plasmodium falciparum CD01

	Total	TP	FP	TN	FN	Sensitivity	Speficity	Recall	Precision	Error rate	AUC
PLA000	47	18	29	15	8	0.692307692307692	0.340909090909091	0.659090909090909	0.382978723404255	0.528571428571429	0.436274509803922
PLA001	33	22	11	29	5	0.814814814814815	0.725	0.275	0.666666666666667	0.238805970149254	0.73406862745098
PLA002	33	22	11	29	5	0.814814814814815	0.725	0.275	0.666666666666667	0.238805970149254	0.73406862745098
PLA003	31	22	9	31	5	0.814814814814815	0.775	0.225	0.709677419354839	0.208955223880597	0.763480392156863
PLA004	32	22	10	30	5	0.814814814814815	0.75	0.25	0.6875	0.223880597014925	0.748774509803921
PLA005	31	22	9	31	5	0.814814814814815	0.775	0.225	0.709677419354839	0.208955223880597	0.763480392156863
PLA006	32	22	10	30	5	0.814814814814815	0.75	0.25	0.6875	0.223880597014925	0.748774509803921
PLA007	33	22	11	29	5	0.814814814814815	0.725	0.275	0.666666666666667	0.238805970149254	0.73406862745098
PLA008	33	22	11	29	5	0.814814814814815	0.725	0.275	0.666666666666667	0.238805970149254	0.73406862745098
PLA009	38	23	15	24	4	0.851851851851852	0.615384615384615	0.384615384615385	0.605263157894737	0.287878787878788	0.71078431372549
PLA010	38	23	15	24	4	0.851851851851852	0.615384615384615	0.384615384615385	0.605263157894737	0.287878787878788	0.71078431372549
PLA011	44	24	20	18	3	0.888888888888889	0.473684210526316	0.526315789473684	0.545454545454545	0.353846153846154	0.658088235294118
PLA012	44	24	20	18	3	0.888888888888889	0.473684210526316	0.526315789473684	0.545454545454545	0.353846153846154	0.658088235294118
PLA013	49	23	26	13	4	0.851851851851852	0.333333333333333	0.666666666666667	0.469387755102041	0.454545454545455	0.57843137254902
PLA014	49	22	27	13	5	0.814814814814815	0.325	0.675	0.448979591836735	0.477611940298507	0.542892156862745
PLA015	48	22	26	14	5	0.814814814814815	0.35	0.65	0.458333333333333	0.462686567164179	0.557598039215686
PLA016	47	22	25	15	5	0.814814814814815	0.375	0.625	0.468085106382979	0.447761194029851	0.572303921568627
PLA017	31	20	11	31	7	0.740740740740741	0.738095238095238	0.261904761904762	0.645161290322581	0.260869565217391	0.692401960784314
PLA018	35	21	14	27	6	0.777777777777778	0.658536585365854	0.341463414634146	0.6	0.294117647058824	0.683823529411765
PLA019	39	22	17	23	5	0.814814814814815	0.575	0.425	0.564102564102564	0.328358208955224	0.660539215686274
PLA020	39	22	17	23	5	0.814814814814815	0.575	0.425	0.564102564102564	0.328358208955224	0.660539215686274
PLA021	41	22	19	21	5	0.814814814814815	0.525	0.475	0.536585365853659	0.358208955223881	0.631127450980392
PLA022	41	22	19	21	5	0.814814814814815	0.525	0.475	0.536585365853659	0.358208955223881	0.631127450980392
PLA023	45	22	23	17	5	0.814814814814815	0.425	0.575	0.488888888888889	0.417910447761194	0.60171568627451
PLA024	42	22	20	20	5	0.814814814814815	0.5	0.5	0.523809523809524	0.373134328358209	0.645833333333333
PLA025	43	23	20	19	4	0.851851851851852	0.487179487179487	0.512820512820513	0.534883720930233	0.363636363636364	0.666666666666667
PLA026	41	23	18	21	4	0.851851851851852	0.538461538461538	0.461538461538462	0.560975609756098	0.333333333333333	0.681372549019608
PLA027	35	22	13	27	5	0.814814814814815	0.675	0.325	0.628571428571429	0.26865671641791	0.719362745098039
PLA028	32	22	10	30	5	0.814814814814815	0.75	0.25	0.6875	0.223880597014925	0.763480392156863
PLA029	30	21	9	32	6	0.777777777777778	0.780487804878049	0.219512195121951	0.7	0.220588235294118	0.742647058823529
PLA030	31	22	9	31	5	0.814814814814815	0.775	0.225	0.709677419354839	0.208955223880597	0.763480392156863

Plasmodium falciparum Dd2

	Total	TP	FP	TN	FN	Sensitivity	Speficity	Recall	Precision	Error rate	AUC
PLA000	48	19	29	14	7	0.730769230769231	0.325581395348837	0.674418604651163	0.395833333333333	0.521739130434783	0.445075757575758
PLA001	31	22	9	31	5	0.814814814814815	0.775	0.225	0.709677419354839	0.208955223880597	0.759469696969697
PLA002	32	22	10	30	5	0.814814814814815	0.75	0.25	0.6875	0.223880597014925	0.759469696969697
PLA003	30	22	8	32	5	0.814814814814815	0.8	0.2	0.733333333333333	0.194029850746269	0.774621212121212
PLA004	31	22	9	31	5	0.814814814814815	0.775	0.225	0.709677419354839	0.208955223880597	0.774621212121212
PLA005	30	22	8	32	5	0.814814814814815	0.8	0.2	0.733333333333333	0.194029850746269	0.774621212121212
PLA006	31	22	9	31	5	0.814814814814815	0.775	0.225	0.709677419354839	0.208955223880597	0.774621212121212
PLA007	31	22	9	31	5	0.814814814814815	0.775	0.225	0.709677419354839	0.208955223880597	0.759469696969697
PLA008	32	22	10	30	5	0.814814814814815	0.75	0.25	0.6875	0.223880597014925	0.759469696969697
PLA009	36	23	13	26	4	0.851851851851852	0.666666666666667	0.333333333333333	0.638888888888889	0.257575757575758	0.734848484848485
PLA010	36	23	13	26	4	0.851851851851852	0.666666666666667	0.333333333333333	0.638888888888889	0.257575757575758	0.734848484848485
PLA011	41	24	17	21	3	0.888888888888889	0.552631578947368	0.447368421052632	0.585365853658537	0.307692307692308	0.695075757575758
PLA012	41	24	17	21	3	0.888888888888889	0.552631578947368	0.447368421052632	0.585365853658537	0.307692307692308	0.695075757575758
PLA013	46	23	23	16	4	0.851851851851852	0.41025641025641	0.58974358974359	0.5	0.409090909090909	0.613636363636364
PLA014	46	22	24	16	5	0.814814814814815	0.4	0.6	0.478260869565217	0.432835820895522	0.577651515151515
PLA015	45	22	23	17	5	0.814814814814815	0.425	0.575	0.488888888888889	0.417910447761194	0.59280303030303
PLA016	44	22	22	18	5	0.814814814814815	0.45	0.55	0.5	0.402985074626866	0.607954545454545
PLA017	30	20	10	32	7	0.740740740740741	0.761904761904762	0.238095238095238	0.666666666666667	0.246376811594203	0.71780303030303
PLA018	33	21	12	29	6	0.777777777777778	0.707317073170732	0.292682926829268	0.636363636363636	0.264705882352941	0.708333333333333
PLA019	38	22	16	24	5	0.814814814814815	0.6	0.4	0.578947368421053	0.313432835820896	0.668560606060606
PLA020	38	22	16	24	5	0.814814814814815	0.6	0.4	0.578947368421053	0.313432835820896	0.668560606060606
PLA021	38	22	16	24	5	0.814814814814815	0.6	0.4	0.578947368421053	0.313432835820896	0.668560606060606
PLA022	38	22	16	24	5	0.814814814814815	0.6	0.4	0.578947368421053	0.313432835820896	0.668560606060606
PLA023	43	22	21	19	5	0.814814814814815	0.475	0.525	0.511627906976744	0.388059701492537	0.623106060606061
PLA024	40	21	19	22	6	0.777777777777778	0.536585365853659	0.463414634146341	0.525	0.367647058823529	0.632575757575758
PLA025	41	22	19	21	5	0.814814814814815	0.525	0.475	0.536585365853659	0.358208955223881	0.653409090909091
PLA026	40	22	18	22	5	0.814814814814815	0.55	0.45	0.55	0.343283582089552	0.653409090909091
PLA027	34	21	13	28	6	0.777777777777778	0.682926829268293	0.317073170731707	0.617647058823529	0.279411764705882	0.693181818181818
PLA028	31	21	10	31	6	0.777777777777778	0.75609756097561	0.24390243902439	0.67741935483871	0.235294117647059	0.738636363636364
PLA029	30	20	10	32	7	0.740740740740741	0.761904761904762	0.238095238095238	0.666666666666667	0.246376811594203	0.71780303030303
PLA030	31	22	9	31	5	0.814814814814815	0.775	0.225	0.709677419354839	0.208955223880597	0.774621212121212

Plasmodium falciparum GA01

	Total	TP	FP	TN	FN	Sensitivity	Speficity	Recall	Precision	Error rate	AUC
PLA000	46	19	27	18	7	0.730769230769231	0.4	0.6	0.41304347826087	0.47887323943662	0.486519607843137
PLA001	32	21	11	32	6	0.777777777777778	0.744186046511628	0.255813953488372	0.65625	0.242857142857143	0.713235294117647
PLA002	33	21	12	31	6	0.777777777777778	0.720930232558139	0.27906976744186	0.636363636363636	0.257142857142857	0.698529411764706
PLA003	31	21	10	33	6	0.777777777777778	0.767441860465116	0.232558139534884	0.67741935483871	0.228571428571429	0.727941176470588
PLA004	32	21	11	32	6	0.777777777777778	0.744186046511628	0.255813953488372	0.65625	0.242857142857143	0.713235294117647
PLA005	31	21	10	33	6	0.777777777777778	0.767441860465116	0.232558139534884	0.67741935483871	0.228571428571429	0.727941176470588
PLA006	32	21	11	32	6	0.777777777777778	0.744186046511628	0.255813953488372	0.65625	0.242857142857143	0.713235294117647
PLA007	32	21	11	32	6	0.777777777777778	0.744186046511628	0.255813953488372	0.65625	0.242857142857143	0.713235294117647
PLA008	33	21	12	31	6	0.777777777777778	0.720930232558139	0.27906976744186	0.636363636363636	0.257142857142857	0.698529411764706
PLA009	38	22	16	26	5	0.814814814814815	0.619047619047619	0.380952380952381	0.578947368421053	0.304347826086957	0.675245098039216
PLA010	38	22	16	26	5	0.814814814814815	0.619047619047619	0.380952380952381	0.578947368421053	0.304347826086957	0.675245098039216
PLA011	43	23	20	21	4	0.851851851851852	0.51219512195122	0.48780487804878	0.534883720930233	0.352941176470588	0.637254901960784
PLA012	43	23	20	21	4	0.851851851851852	0.51219512195122	0.48780487804878	0.534883720930233	0.352941176470588	0.637254901960784
PLA013	49	22	27	15	5	0.814814814814815	0.357142857142857	0.642857142857143	0.448979591836735	0.463768115942029	0.557598039215686
PLA014	49	21	28	15	6	0.777777777777778	0.348837209302326	0.651162790697674	0.428571428571429	0.485714285714286	0.536764705882353
PLA015	47	21	26	17	6	0.777777777777778	0.395348837209302	0.604651162790698	0.446808510638298	0.457142857142857	0.551470588235294
PLA016	45	20	25	19	7	0.740740740740741	0.431818181818182	0.568181818181818	0.444444444444444	0.450704225352113	0.545343137254902
PLA017	32	19	13	32	8	0.703703703703704	0.711111111111111	0.288888888888889	0.59375	0.291666666666667	0.642156862745098
PLA018	35	19	16	29	8	0.703703703703704	0.644444444444444	0.355555555555556	0.542857142857143	0.333333333333333	0.612745098039216
PLA019	39	20	19	25	7	0.740740740740741	0.568181818181818	0.431818181818182	0.512820512820513	0.366197183098592	0.589460784313726
PLA020	39	20	19	25	7	0.740740740740741	0.568181818181818	0.431818181818182	0.512820512820513	0.366197183098592	0.589460784313726
PLA021	39	20	19	25	7	0.740740740740741	0.568181818181818	0.431818181818182	0.512820512820513	0.366197183098592	0.589460784313726
PLA022	39	20	19	25	7	0.740740740740741	0.568181818181818	0.431818181818182	0.512820512820513	0.366197183098592	0.589460784313726
PLA023	45	20	25	19	7	0.740740740740741	0.431818181818182	0.568181818181818	0.444444444444444	0.450704225352113	0.560049019607843
PLA024	44	20	24	20	7	0.740740740740741	0.454545454545455	0.545454545454545	0.454545454545455	0.436619718309859	0.574754901960784
PLA025	45	21	24	19	6	0.777777777777778	0.441860465116279	0.558139534883721	0.466666666666667	0.428571428571429	0.595588235294118
PLA026	43	21	22	21	6	0.777777777777778	0.488372093023256	0.511627906976744	0.488372093023256	0.4	0.610294117647059
PLA027	36	20	16	28	7	0.740740740740741	0.636363636363636	0.363636363636364	0.555555555555556	0.323943661971831	0.633578431372549
PLA028	33	20	13	31	7	0.740740740740741	0.704545454545455	0.295454545454545	0.606060606060606	0.28169014084507	0.677696078431373
PLA029	32	20	12	32	7	0.740740740740741	0.727272727272727	0.272727272727273	0.625	0.267605633802817	0.677696078431373
PLA030	31	21	10	33	6	0.777777777777778	0.767441860465116	0.232558139534884	0.67741935483871	0.228571428571429	0.727941176470588

Plasmodium falciparum GB4

	Total	TP	FP	TN	FN	Sensitivity	Speficity	Recall	Precision	Error rate	AUC
PLA000	51	19	32	15	7	0.730769230769231	0.319148936170213	0.680851063829787	0.372549019607843	0.534246575342466	0.454166666666667
PLA001	33	22	11	33	5	0.814814814814815	0.75	0.25	0.666666666666667	0.225352112676056	0.738690476190476
PLA002	34	22	12	32	5	0.814814814814815	0.727272727272727	0.272727272727273	0.647058823529412	0.23943661971831	0.738690476190476
PLA003	33	22	11	33	5	0.814814814814815	0.75	0.25	0.666666666666667	0.225352112676056	0.738690476190476
PLA004	34	22	12	32	5	0.814814814814815	0.727272727272727	0.272727272727273	0.647058823529412	0.23943661971831	0.738690476190476
PLA005	33	22	11	33	5	0.814814814814815	0.75	0.25	0.666666666666667	0.225352112676056	0.738690476190476
PLA006	34	22	12	32	5	0.814814814814815	0.727272727272727	0.272727272727273	0.647058823529412	0.23943661971831	0.738690476190476
PLA007	33	22	11	33	5	0.814814814814815	0.75	0.25	0.666666666666667	0.225352112676056	0.738690476190476
PLA008	34	22	12	32	5	0.814814814814815	0.727272727272727	0.272727272727273	0.647058823529412	0.23943661971831	0.738690476190476
PLA009	39	23	16	27	4	0.851851851851852	0.627906976744186	0.372093023255814	0.58974358974359	0.285714285714286	0.716666666666667
PLA010	39	23	16	27	4	0.851851851851852	0.627906976744186	0.372093023255814	0.58974358974359	0.285714285714286	0.716666666666667
PLA011	44	24	20	22	3	0.888888888888889	0.523809523809524	0.476190476190476	0.545454545454545	0.333333333333333	0.680357142857143
PLA012	44	24	20	22	3	0.888888888888889	0.523809523809524	0.476190476190476	0.545454545454545	0.333333333333333	0.680357142857143
PLA013	49	23	26	17	4	0.851851851851852	0.395348837209302	0.604651162790698	0.469387755102041	0.428571428571429	0.602380952380952
PLA014	49	22	27	17	5	0.814814814814815	0.386363636363636	0.613636363636364	0.448979591836735	0.450704225352113	0.567261904761905
PLA015	48	22	26	18	5	0.814814814814815	0.409090909090909	0.590909090909091	0.458333333333333	0.436619718309859	0.581547619047619
PLA016	47	22	25	19	5	0.814814814814815	0.431818181818182	0.568181818181818	0.468085106382979	0.422535211267606	0.595833333333333
PLA017	32	20	12	34	7	0.740740740740741	0.739130434782609	0.260869565217391	0.625	0.26027397260274	0.69702380952381
PLA018	36	21	15	30	6	0.777777777777778	0.666666666666667	0.333333333333333	0.583333333333333	0.291666666666667	0.689285714285714
PLA019	41	22	19	25	5	0.814814814814815	0.568181818181818	0.431818181818182	0.536585365853659	0.338028169014085	0.65297619047619
PLA020	41	22	19	25	5	0.814814814814815	0.568181818181818	0.431818181818182	0.536585365853659	0.338028169014085	0.65297619047619
PLA021	41	22	19	25	5	0.814814814814815	0.568181818181818	0.431818181818182	0.536585365853659	0.338028169014085	0.65297619047619
PLA022	41	22	19	25	5	0.814814814814815	0.568181818181818	0.431818181818182	0.536585365853659	0.338028169014085	0.65297619047619
PLA023	45	22	23	21	5	0.814814814814815	0.477272727272727	0.522727272727273	0.488888888888889	0.394366197183099	0.624404761904762
PLA024	43	22	21	23	5	0.814814814814815	0.522727272727273	0.477272727272727	0.511627906976744	0.366197183098592	0.65297619047619
PLA025	44	23	21	22	4	0.851851851851852	0.511627906976744	0.488372093023256	0.522727272727273	0.357142857142857	0.673809523809524
PLA026	42	23	19	24	4	0.851851851851852	0.558139534883721	0.441860465116279	0.547619047619048	0.328571428571429	0.688095238095238
PLA027	37	22	15	29	5	0.814814814814815	0.659090909090909	0.340909090909091	0.594594594594595	0.28169014084507	0.710119047619048
PLA028	34	22	12	32	5	0.814814814814815	0.727272727272727	0.272727272727273	0.647058823529412	0.23943661971831	0.75297619047619
PLA029	32	21	11	34	6	0.777777777777778	0.755555555555556	0.244444444444444	0.65625	0.236111111111111	0.732142857142857
PLA030	33	22	11	33	5	0.814814814814815	0.75	0.25	0.666666666666667	0.225352112676056	0.75297619047619

Plasmodium falciparum GN01

	Total	TP	FP	TN	FN	Sensitivity	Speficity	Recall	Precision	Error rate	AUC
PLA000	55	19	36	14	7	0.730769230769231	0.28	0.72	0.345454545454545	0.565789473684211	0.429924242424242
PLA001	33	22	11	36	5	0.814814814814815	0.765957446808511	0.234042553191489	0.666666666666667	0.216216216216216	0.729166666666667
PLA002	33	22	11	36	5	0.814814814814815	0.765957446808511	0.234042553191489	0.666666666666667	0.216216216216216	0.729166666666667
PLA003	32	22	10	37	5	0.814814814814815	0.787234042553192	0.212765957446809	0.6875	0.202702702702703	0.744318181818182
PLA004	32	22	10	37	5	0.814814814814815	0.787234042553192	0.212765957446809	0.6875	0.202702702702703	0.744318181818182
PLA005	32	22	10	37	5	0.814814814814815	0.787234042553192	0.212765957446809	0.6875	0.202702702702703	0.744318181818182
PLA006	32	22	10	37	5	0.814814814814815	0.787234042553192	0.212765957446809	0.6875	0.202702702702703	0.744318181818182
PLA007	33	22	11	36	5	0.814814814814815	0.765957446808511	0.234042553191489	0.666666666666667	0.216216216216216	0.729166666666667
PLA008	33	22	11	36	5	0.814814814814815	0.765957446808511	0.234042553191489	0.666666666666667	0.216216216216216	0.729166666666667
PLA009	38	23	15	31	4	0.851851851851852	0.673913043478261	0.326086956521739	0.605263157894737	0.26027397260274	0.704545454545455
PLA010	38	23	15	31	4	0.851851851851852	0.673913043478261	0.326086956521739	0.605263157894737	0.26027397260274	0.704545454545455
PLA011	44	24	20	25	3	0.888888888888889	0.555555555555556	0.444444444444444	0.545454545454545	0.319444444444444	0.649621212121212
PLA012	44	24	20	25	3	0.888888888888889	0.555555555555556	0.444444444444444	0.545454545454545	0.319444444444444	0.649621212121212
PLA013	50	23	27	19	4	0.851851851851852	0.41304347826087	0.58695652173913	0.46	0.424657534246575	0.568181818181818
PLA014	49	22	27	20	5	0.814814814814815	0.425531914893617	0.574468085106383	0.448979591836735	0.432432432432432	0.547348484848485
PLA015	47	22	25	22	5	0.814814814814815	0.468085106382979	0.531914893617021	0.468085106382979	0.405405405405405	0.5625
PLA016	46	22	24	23	5	0.814814814814815	0.48936170212766	0.51063829787234	0.478260869565217	0.391891891891892	0.577651515151515
PLA017	31	20	11	38	7	0.740740740740741	0.775510204081633	0.224489795918367	0.645161290322581	0.236842105263158	0.6875
PLA018	35	21	14	34	6	0.777777777777778	0.708333333333333	0.291666666666667	0.6	0.266666666666667	0.678030303030303
PLA019	38	22	16	31	5	0.814814814814815	0.659574468085106	0.340425531914894	0.578947368421053	0.283783783783784	0.668560606060606
PLA020	38	22	16	31	5	0.814814814814815	0.659574468085106	0.340425531914894	0.578947368421053	0.283783783783784	0.668560606060606
PLA021	40	22	18	29	5	0.814814814814815	0.617021276595745	0.382978723404255	0.55	0.310810810810811	0.638257575757576
PLA022	40	22	18	29	5	0.814814814814815	0.617021276595745	0.382978723404255	0.55	0.310810810810811	0.638257575757576
PLA023	45	22	23	24	5	0.814814814814815	0.51063829787234	0.48936170212766	0.488888888888889	0.378378378378378	0.607954545454545
PLA024	43	22	21	26	5	0.814814814814815	0.553191489361702	0.446808510638298	0.511627906976744	0.351351351351351	0.638257575757576
PLA025	44	23	21	25	4	0.851851851851852	0.543478260869565	0.456521739130435	0.522727272727273	0.342465753424658	0.659090909090909
PLA026	42	23	19	27	4	0.851851851851852	0.58695652173913	0.41304347826087	0.547619047619048	0.315068493150685	0.674242424242424
PLA027	35	22	13	34	5	0.814814814814815	0.723404255319149	0.276595744680851	0.628571428571429	0.243243243243243	0.714015151515151
PLA028	32	22	10	37	5	0.814814814814815	0.787234042553192	0.212765957446809	0.6875	0.202702702702703	0.759469696969697
PLA029	30	21	9	39	6	0.777777777777778	0.8125	0.1875	0.7	0.2	0.738636363636364
PLA030	31	22	9	38	5	0.814814814814815	0.808510638297872	0.191489361702128	0.709677419354839	0.189189189189189	0.759469696969697

Plasmodium falciparum HB3

	Total	TP	FP	TN	FN	Sensitivity	Speficity	Recall	Precision	Error rate	AUC
PLA000	46	19	27	15	7	0.730769230769231	0.357142857142857	0.642857142857143	0.41304347826087	0.5	0.450940860215054
PLA001	29	21	8	32	6	0.777777777777778	0.8	0.2	0.724137931034483	0.208955223880597	0.745967741935484
PLA002	30	21	9	31	6	0.777777777777778	0.775	0.225	0.7	0.223880597014925	0.745967741935484
PLA003	29	21	8	32	6	0.777777777777778	0.8	0.2	0.724137931034483	0.208955223880597	0.745967741935484
PLA004	30	21	9	31	6	0.777777777777778	0.775	0.225	0.7	0.223880597014925	0.745967741935484
PLA005	29	21	8	32	6	0.777777777777778	0.8	0.2	0.724137931034483	0.208955223880597	0.745967741935484
PLA006	30	21	9	31	6	0.777777777777778	0.775	0.225	0.7	0.223880597014925	0.745967741935484
PLA007	29	21	8	32	6	0.777777777777778	0.8	0.2	0.724137931034483	0.208955223880597	0.745967741935484
PLA008	30	21	9	31	6	0.777777777777778	0.775	0.225	0.7	0.223880597014925	0.745967741935484
PLA009	36	22	14	25	5	0.814814814814815	0.641025641025641	0.358974358974359	0.611111111111111	0.287878787878788	0.702284946236559
PLA010	36	22	14	25	5	0.814814814814815	0.641025641025641	0.358974358974359	0.611111111111111	0.287878787878788	0.702284946236559
PLA011	41	23	18	20	4	0.851851851851852	0.526315789473684	0.473684210526316	0.560975609756098	0.338461538461538	0.658602150537634
PLA012	41	23	18	20	4	0.851851851851852	0.526315789473684	0.473684210526316	0.560975609756098	0.338461538461538	0.658602150537634
PLA013	48	22	26	13	5	0.814814814814815	0.333333333333333	0.666666666666667	0.458333333333333	0.46969696969697	0.557123655913978
PLA014	47	21	26	14	6	0.777777777777778	0.35	0.65	0.446808510638298	0.477611940298507	0.536290322580645
PLA015	46	21	25	15	6	0.777777777777778	0.375	0.625	0.456521739130435	0.462686567164179	0.55241935483871
PLA016	45	21	24	16	6	0.777777777777778	0.4	0.6	0.466666666666667	0.447761194029851	0.568548387096774
PLA017	28	19	9	33	8	0.703703703703704	0.785714285714286	0.214285714285714	0.678571428571429	0.246376811594203	0.704301075268817
PLA018	34	20	14	27	7	0.740740740740741	0.658536585365854	0.341463414634146	0.588235294117647	0.308823529411765	0.660618279569892
PLA019	38	21	17	23	6	0.777777777777778	0.575	0.425	0.552631578947368	0.343283582089552	0.633064516129032
PLA020	38	21	17	23	6	0.777777777777778	0.575	0.425	0.552631578947368	0.343283582089552	0.633064516129032
PLA021	38	21	17	23	6	0.777777777777778	0.575	0.425	0.552631578947368	0.343283582089552	0.633064516129032
PLA022	38	21	17	23	6	0.777777777777778	0.575	0.425	0.552631578947368	0.343283582089552	0.633064516129032
PLA023	43	21	22	18	6	0.777777777777778	0.45	0.55	0.488372093023256	0.417910447761194	0.600806451612903
PLA024	41	20	21	20	7	0.740740740740741	0.48780487804878	0.51219512195122	0.48780487804878	0.411764705882353	0.596102150537634
PLA025	42	21	21	19	6	0.777777777777778	0.475	0.525	0.5	0.402985074626866	0.616935483870968
PLA026	41	21	20	20	6	0.777777777777778	0.5	0.5	0.51219512195122	0.388059701492537	0.616935483870968
PLA027	35	20	15	26	7	0.740740740740741	0.634146341463415	0.365853658536585	0.571428571428571	0.323529411764706	0.644489247311828
PLA028	32	20	12	29	7	0.740740740740741	0.707317073170732	0.292682926829268	0.625	0.279411764705882	0.692876344086022
PLA029	28	19	9	33	8	0.703703703703704	0.785714285714286	0.214285714285714	0.678571428571429	0.246376811594203	0.704301075268817
PLA030	30	21	9	31	6	0.777777777777778	0.775	0.225	0.7	0.223880597014925	0.745967741935484

Plasmodium falciparum IT

	Total	TP	FP	TN	FN	Sensitivity	Speficity	Recall	Precision	Error rate	AUC
PLA000	47	19	28	18	7	0.730769230769231	0.391304347826087	0.608695652173913	0.404255319148936	0.486111111111111	0.460227272727273
PLA001	31	22	9	34	5	0.814814814814815	0.790697674418605	0.209302325581395	0.709677419354839	0.2	0.759469696969697
PLA002	31	22	9	34	5	0.814814814814815	0.790697674418605	0.209302325581395	0.709677419354839	0.2	0.759469696969697
PLA003	30	22	8	35	5	0.814814814814815	0.813953488372093	0.186046511627907	0.733333333333333	0.185714285714286	0.774621212121212
PLA004	30	22	8	35	5	0.814814814814815	0.813953488372093	0.186046511627907	0.733333333333333	0.185714285714286	0.774621212121212
PLA005	30	22	8	35	5	0.814814814814815	0.813953488372093	0.186046511627907	0.733333333333333	0.185714285714286	0.774621212121212
PLA006	30	22	8	35	5	0.814814814814815	0.813953488372093	0.186046511627907	0.733333333333333	0.185714285714286	0.774621212121212
PLA007	31	22	9	34	5	0.814814814814815	0.790697674418605	0.209302325581395	0.709677419354839	0.2	0.759469696969697
PLA008	31	22	9	34	5	0.814814814814815	0.790697674418605	0.209302325581395	0.709677419354839	0.2	0.759469696969697
PLA009	37	23	14	28	4	0.851851851851852	0.666666666666667	0.333333333333333	0.621621621621622	0.260869565217391	0.734848484848485
PLA010	37	23	14	28	4	0.851851851851852	0.666666666666667	0.333333333333333	0.621621621621622	0.260869565217391	0.734848484848485
PLA011	43	24	19	22	3	0.888888888888889	0.536585365853659	0.463414634146341	0.558139534883721	0.323529411764706	0.679924242424242
PLA012	43	24	19	22	3	0.888888888888889	0.536585365853659	0.463414634146341	0.558139534883721	0.323529411764706	0.679924242424242
PLA013	50	23	27	15	4	0.851851851851852	0.357142857142857	0.642857142857143	0.46	0.449275362318841	0.598484848484849
PLA014	50	22	28	15	5	0.814814814814815	0.348837209302326	0.651162790697674	0.44	0.471428571428571	0.5625
PLA015	48	22	26	17	5	0.814814814814815	0.395348837209302	0.604651162790698	0.458333333333333	0.442857142857143	0.577651515151515
PLA016	47	22	25	18	5	0.814814814814815	0.418604651162791	0.581395348837209	0.468085106382979	0.428571428571429	0.59280303030303
PLA017	29	20	9	36	7	0.740740740740741	0.8	0.2	0.689655172413793	0.222222222222222	0.71780303030303
PLA018	35	21	14	30	6	0.777777777777778	0.681818181818182	0.318181818181818	0.6	0.28169014084507	0.693181818181818
PLA019	40	22	18	25	5	0.814814814814815	0.581395348837209	0.418604651162791	0.55	0.328571428571429	0.653409090909091
PLA020	40	22	18	25	5	0.814814814814815	0.581395348837209	0.418604651162791	0.55	0.328571428571429	0.653409090909091
PLA021	41	22	19	24	5	0.814814814814815	0.558139534883721	0.441860465116279	0.536585365853659	0.342857142857143	0.638257575757576
PLA022	41	22	19	24	5	0.814814814814815	0.558139534883721	0.441860465116279	0.536585365853659	0.342857142857143	0.638257575757576
PLA023	46	21	25	19	6	0.777777777777778	0.431818181818182	0.568181818181818	0.456521739130435	0.436619718309859	0.587121212121212
PLA024	43	21	22	22	6	0.777777777777778	0.5	0.5	0.488372093023256	0.394366197183099	0.632575757575758
PLA025	44	22	22	21	5	0.814814814814815	0.488372093023256	0.511627906976744	0.5	0.385714285714286	0.653409090909091
PLA026	43	22	21	22	5	0.814814814814815	0.511627906976744	0.488372093023256	0.511627906976744	0.371428571428571	0.653409090909091
PLA027	36	21	15	29	6	0.777777777777778	0.659090909090909	0.340909090909091	0.583333333333333	0.295774647887324	0.678030303030303
PLA028	33	21	12	32	6	0.777777777777778	0.727272727272727	0.272727272727273	0.636363636363636	0.253521126760563	0.723484848484849
PLA029	29	20	9	36	7	0.740740740740741	0.8	0.2	0.689655172413793	0.222222222222222	0.71780303030303
PLA030	30	22	8	35	5	0.814814814814815	0.813953488372093	0.186046511627907	0.733333333333333	0.185714285714286	0.774621212121212

Plasmodium falciparum KE01

	Total	TP	FP	TN	FN	Sensitivity	Speficity	Recall	Precision	Error rate	AUC
PLA000	49	19	30	13	7	0.730769230769231	0.302325581395349	0.697674418604651	0.387755102040816	0.536231884057971	0.442401960784314
PLA001	31	22	9	31	5	0.814814814814815	0.775	0.225	0.709677419354839	0.208955223880597	0.763480392156863
PLA002	31	22	9	31	5	0.814814814814815	0.775	0.225	0.709677419354839	0.208955223880597	0.763480392156863
PLA003	31	22	9	31	5	0.814814814814815	0.775	0.225	0.709677419354839	0.208955223880597	0.763480392156863
PLA004	31	22	9	31	5	0.814814814814815	0.775	0.225	0.709677419354839	0.208955223880597	0.763480392156863
PLA005	31	22	9	31	5	0.814814814814815	0.775	0.225	0.709677419354839	0.208955223880597	0.763480392156863
PLA006	31	22	9	31	5	0.814814814814815	0.775	0.225	0.709677419354839	0.208955223880597	0.763480392156863
PLA007	31	22	9	31	5	0.814814814814815	0.775	0.225	0.709677419354839	0.208955223880597	0.763480392156863
PLA008	31	22	9	31	5	0.814814814814815	0.775	0.225	0.709677419354839	0.208955223880597	0.763480392156863
PLA009	36	23	13	26	4	0.851851851851852	0.666666666666667	0.333333333333333	0.638888888888889	0.257575757575758	0.725490196078431
PLA010	36	23	13	26	4	0.851851851851852	0.666666666666667	0.333333333333333	0.638888888888889	0.257575757575758	0.725490196078431
PLA011	42	24	18	20	3	0.888888888888889	0.526315789473684	0.473684210526316	0.571428571428571	0.323076923076923	0.6875
PLA012	42	24	18	20	3	0.888888888888889	0.526315789473684	0.473684210526316	0.571428571428571	0.323076923076923	0.6875
PLA013	48	23	25	14	4	0.851851851851852	0.358974358974359	0.641025641025641	0.479166666666667	0.439393939393939	0.593137254901961
PLA014	48	22	26	14	5	0.814814814814815	0.35	0.65	0.458333333333333	0.462686567164179	0.557598039215686
PLA015	47	22	25	15	5	0.814814814814815	0.375	0.625	0.468085106382979	0.447761194029851	0.572303921568627
PLA016	46	22	24	16	5	0.814814814814815	0.4	0.6	0.478260869565217	0.432835820895522	0.587009803921569
PLA017	29	20	9	33	7	0.740740740740741	0.785714285714286	0.214285714285714	0.689655172413793	0.231884057971014	0.721813725490196
PLA018	33	21	12	29	6	0.777777777777778	0.707317073170732	0.292682926829268	0.636363636363636	0.264705882352941	0.698529411764706
PLA019	40	22	18	22	5	0.814814814814815	0.55	0.45	0.55	0.343283582089552	0.645833333333333
PLA020	40	22	18	22	5	0.814814814814815	0.55	0.45	0.55	0.343283582089552	0.645833333333333
PLA021	39	22	17	23	5	0.814814814814815	0.575	0.425	0.564102564102564	0.328358208955224	0.660539215686274
PLA022	39	22	17	23	5	0.814814814814815	0.575	0.425	0.564102564102564	0.328358208955224	0.660539215686274
PLA023	45	22	23	17	5	0.814814814814815	0.425	0.575	0.488888888888889	0.417910447761194	0.60171568627451
PLA024	43	22	21	19	5	0.814814814814815	0.475	0.525	0.511627906976744	0.388059701492537	0.631127450980392
PLA025	44	23	21	18	4	0.851851851851852	0.461538461538462	0.538461538461538	0.522727272727273	0.378787878787879	0.651960784313726
PLA026	41	23	18	21	4	0.851851851851852	0.538461538461538	0.461538461538462	0.560975609756098	0.333333333333333	0.666666666666667
PLA027	35	22	13	27	5	0.814814814814815	0.675	0.325	0.628571428571429	0.26865671641791	0.704656862745098
PLA028	32	22	10	30	5	0.814814814814815	0.75	0.25	0.6875	0.223880597014925	0.748774509803921
PLA029	30	21	9	32	6	0.777777777777778	0.780487804878049	0.219512195121951	0.7	0.220588235294118	0.742647058823529
PLA030	30	22	8	32	5	0.814814814814815	0.8	0.2	0.733333333333333	0.194029850746269	0.778186274509804

Plasmodium falciparum KH01

	Total	TP	FP	TN	FN	Sensitivity	Speficity	Recall	Precision	Error rate	AUC
PLA000	50	19	31	17	7	0.730769230769231	0.354166666666667	0.645833333333333	0.38	0.513513513513513	0.479166666666667
PLA001	32	21	11	35	6	0.777777777777778	0.760869565217391	0.239130434782609	0.65625	0.232876712328767	0.722222222222222
PLA002	32	21	11	35	6	0.777777777777778	0.760869565217391	0.239130434782609	0.65625	0.232876712328767	0.722222222222222
PLA003	31	21	10	36	6	0.777777777777778	0.782608695652174	0.217391304347826	0.67741935483871	0.219178082191781	0.736111111111111
PLA004	31	21	10	36	6	0.777777777777778	0.782608695652174	0.217391304347826	0.67741935483871	0.219178082191781	0.736111111111111
PLA005	31	21	10	36	6	0.777777777777778	0.782608695652174	0.217391304347826	0.67741935483871	0.219178082191781	0.736111111111111
PLA006	31	21	10	36	6	0.777777777777778	0.782608695652174	0.217391304347826	0.67741935483871	0.219178082191781	0.736111111111111
PLA007	32	21	11	35	6	0.777777777777778	0.760869565217391	0.239130434782609	0.65625	0.232876712328767	0.722222222222222
PLA008	32	21	11	35	6	0.777777777777778	0.760869565217391	0.239130434782609	0.65625	0.232876712328767	0.722222222222222
PLA009	38	22	16	29	5	0.814814814814815	0.644444444444444	0.355555555555556	0.578947368421053	0.291666666666667	0.6875
PLA010	38	22	16	29	5	0.814814814814815	0.644444444444444	0.355555555555556	0.578947368421053	0.291666666666667	0.6875
PLA011	45	23	22	22	4	0.851851851851852	0.5	0.5	0.511111111111111	0.366197183098592	0.638888888888889
PLA012	45	23	22	22	4	0.851851851851852	0.5	0.5	0.511111111111111	0.366197183098592	0.638888888888889
PLA013	50	21	29	17	6	0.777777777777778	0.369565217391304	0.630434782608696	0.42	0.479452054794521	0.541666666666667
PLA014	49	20	29	18	7	0.740740740740741	0.382978723404255	0.617021276595745	0.408163265306122	0.486486486486487	0.520833333333333
PLA015	46	20	26	21	7	0.740740740740741	0.446808510638298	0.553191489361702	0.434782608695652	0.445945945945946	0.548611111111111
PLA016	45	20	25	22	7	0.740740740740741	0.468085106382979	0.531914893617021	0.444444444444444	0.432432432432432	0.5625
PLA017	29	18	11	38	9	0.666666666666667	0.775510204081633	0.224489795918367	0.620689655172414	0.263157894736842	0.659722222222222
PLA018	33	19	14	34	8	0.703703703703704	0.708333333333333	0.291666666666667	0.575757575757576	0.293333333333333	0.652777777777778
PLA019	38	20	18	29	7	0.740740740740741	0.617021276595745	0.382978723404255	0.526315789473684	0.337837837837838	0.618055555555556
PLA020	38	20	18	29	7	0.740740740740741	0.617021276595745	0.382978723404255	0.526315789473684	0.337837837837838	0.618055555555556
PLA021	39	20	19	28	7	0.740740740740741	0.595744680851064	0.404255319148936	0.512820512820513	0.351351351351351	0.618055555555556
PLA022	39	20	19	28	7	0.740740740740741	0.595744680851064	0.404255319148936	0.512820512820513	0.351351351351351	0.618055555555556
PLA023	46	20	26	21	7	0.740740740740741	0.446808510638298	0.553191489361702	0.434782608695652	0.445945945945946	0.5625
PLA024	43	20	23	24	7	0.740740740740741	0.51063829787234	0.48936170212766	0.465116279069767	0.405405405405405	0.590277777777778
PLA025	44	21	23	23	6	0.777777777777778	0.5	0.5	0.477272727272727	0.397260273972603	0.611111111111111
PLA026	43	21	22	24	6	0.777777777777778	0.521739130434783	0.478260869565217	0.488372093023256	0.383561643835616	0.611111111111111
PLA027	36	20	16	31	7	0.740740740740741	0.659574468085106	0.340425531914894	0.555555555555556	0.310810810810811	0.645833333333333
PLA028	33	20	13	34	7	0.740740740740741	0.723404255319149	0.276595744680851	0.606060606060606	0.27027027027027	0.6875
PLA029	30	19	11	37	8	0.703703703703704	0.770833333333333	0.229166666666667	0.633333333333333	0.253333333333333	0.680555555555555
PLA030	31	21	10	36	6	0.777777777777778	0.782608695652174	0.217391304347826	0.67741935483871	0.219178082191781	0.736111111111111

Plasmodium falciparum KH02

	Total	TP	FP	TN	FN	Sensitivity	Speficity	Recall	Precision	Error rate	AUC
PLA000	51	19	32	16	7	0.730769230769231	0.333333333333333	0.666666666666667	0.372549019607843	0.527027027027027	0.462274774774775
PLA001	32	21	11	35	6	0.777777777777778	0.760869565217391	0.239130434782609	0.65625	0.232876712328767	0.726351351351351
PLA002	33	21	12	34	6	0.777777777777778	0.739130434782609	0.260869565217391	0.636363636363636	0.246575342465753	0.726351351351351
PLA003	32	21	11	35	6	0.777777777777778	0.760869565217391	0.239130434782609	0.65625	0.232876712328767	0.726351351351351
PLA004	33	21	12	34	6	0.777777777777778	0.739130434782609	0.260869565217391	0.636363636363636	0.246575342465753	0.726351351351351
PLA005	32	21	11	35	6	0.777777777777778	0.760869565217391	0.239130434782609	0.65625	0.232876712328767	0.726351351351351
PLA006	33	21	12	34	6	0.777777777777778	0.739130434782609	0.260869565217391	0.636363636363636	0.246575342465753	0.726351351351351
PLA007	32	21	11	35	6	0.777777777777778	0.760869565217391	0.239130434782609	0.65625	0.232876712328767	0.726351351351351
PLA008	33	21	12	34	6	0.777777777777778	0.739130434782609	0.260869565217391	0.636363636363636	0.246575342465753	0.726351351351351
PLA009	38	22	16	29	5	0.814814814814815	0.644444444444444	0.355555555555556	0.578947368421053	0.291666666666667	0.706644144144144
PLA010	38	22	16	29	5	0.814814814814815	0.644444444444444	0.355555555555556	0.578947368421053	0.291666666666667	0.706644144144144
PLA011	43	23	20	24	4	0.851851851851852	0.545454545454545	0.454545454545455	0.534883720930233	0.338028169014085	0.673423423423423
PLA012	43	23	20	24	4	0.851851851851852	0.545454545454545	0.454545454545455	0.534883720930233	0.338028169014085	0.673423423423423
PLA013	49	21	28	18	6	0.777777777777778	0.391304347826087	0.608695652173913	0.428571428571429	0.465753424657534	0.564189189189189
PLA014	49	20	29	18	7	0.740740740740741	0.382978723404255	0.617021276595745	0.408163265306122	0.486486486486487	0.529842342342342
PLA015	47	20	27	20	7	0.740740740740741	0.425531914893617	0.574468085106383	0.425531914893617	0.459459459459459	0.543355855855856
PLA016	46	20	26	21	7	0.740740740740741	0.446808510638298	0.553191489361702	0.434782608695652	0.445945945945946	0.556869369369369
PLA017	30	18	12	37	9	0.666666666666667	0.755102040816326	0.244897959183673	0.6	0.276315789473684	0.663851351351351
PLA018	34	19	15	33	8	0.703703703703704	0.6875	0.3125	0.558823529411765	0.306666666666667	0.657657657657658
PLA019	39	20	19	28	7	0.740740740740741	0.595744680851064	0.404255319148936	0.512820512820513	0.351351351351351	0.624436936936937
PLA020	39	20	19	28	7	0.740740740740741	0.595744680851064	0.404255319148936	0.512820512820513	0.351351351351351	0.624436936936937
PLA021	39	20	19	28	7	0.740740740740741	0.595744680851064	0.404255319148936	0.512820512820513	0.351351351351351	0.624436936936937
PLA022	39	20	19	28	7	0.740740740740741	0.595744680851064	0.404255319148936	0.512820512820513	0.351351351351351	0.624436936936937
PLA023	45	20	25	22	7	0.740740740740741	0.468085106382979	0.531914893617021	0.444444444444444	0.432432432432432	0.583896396396396
PLA024	43	20	23	24	7	0.740740740740741	0.51063829787234	0.48936170212766	0.465116279069767	0.405405405405405	0.610923423423423
PLA025	44	21	23	23	6	0.777777777777778	0.5	0.5	0.477272727272727	0.397260273972603	0.631756756756757
PLA026	43	21	22	24	6	0.777777777777778	0.521739130434783	0.478260869565217	0.488372093023256	0.383561643835616	0.631756756756757
PLA027	36	20	16	31	7	0.740740740740741	0.659574468085106	0.340425531914894	0.555555555555556	0.310810810810811	0.664977477477477
PLA028	33	20	13	34	7	0.740740740740741	0.723404255319149	0.276595744680851	0.606060606060606	0.27027027027027	0.705518018018018
PLA029	31	19	12	36	8	0.703703703703704	0.75	0.25	0.612903225806452	0.266666666666667	0.684684684684685
PLA030	33	21	12	34	6	0.777777777777778	0.739130434782609	0.260869565217391	0.636363636363636	0.246575342465753	0.726351351351351

Plasmodium falciparum ML01

	Total	TP	FP	TN	FN	Sensitivity	Speficity	Recall	Precision	Error rate	AUC
PLA000	54	19	35	16	7	0.730769230769231	0.313725490196078	0.686274509803922	0.351851851851852	0.545454545454545	0.429924242424242
PLA001	31	22	9	39	5	0.814814814814815	0.8125	0.1875	0.709677419354839	0.186666666666667	0.759469696969697
PLA002	32	22	10	38	5	0.814814814814815	0.791666666666667	0.208333333333333	0.6875	0.2	0.759469696969697
PLA003	31	22	9	39	5	0.814814814814815	0.8125	0.1875	0.709677419354839	0.186666666666667	0.759469696969697
PLA004	32	22	10	38	5	0.814814814814815	0.791666666666667	0.208333333333333	0.6875	0.2	0.759469696969697
PLA005	31	22	9	39	5	0.814814814814815	0.8125	0.1875	0.709677419354839	0.186666666666667	0.759469696969697
PLA006	32	22	10	38	5	0.814814814814815	0.791666666666667	0.208333333333333	0.6875	0.2	0.759469696969697
PLA007	31	22	9	39	5	0.814814814814815	0.8125	0.1875	0.709677419354839	0.186666666666667	0.759469696969697
PLA008	32	22	10	38	5	0.814814814814815	0.791666666666667	0.208333333333333	0.6875	0.2	0.759469696969697
PLA009	39	23	16	31	4	0.851851851851852	0.659574468085106	0.340425531914894	0.58974358974359	0.27027027027027	0.71969696969697
PLA010	39	23	16	31	4	0.851851851851852	0.659574468085106	0.340425531914894	0.58974358974359	0.27027027027027	0.71969696969697
PLA011	46	24	22	24	3	0.888888888888889	0.521739130434783	0.478260869565217	0.521739130434783	0.342465753424658	0.664772727272727
PLA012	46	24	22	24	3	0.888888888888889	0.521739130434783	0.478260869565217	0.521739130434783	0.342465753424658	0.664772727272727
PLA013	51	23	28	19	4	0.851851851851852	0.404255319148936	0.595744680851064	0.450980392156863	0.432432432432432	0.583333333333333
PLA014	50	22	28	20	5	0.814814814814815	0.416666666666667	0.583333333333333	0.44	0.44	0.5625
PLA015	49	22	27	21	5	0.814814814814815	0.4375	0.5625	0.448979591836735	0.426666666666667	0.577651515151515
PLA016	47	22	25	23	5	0.814814814814815	0.479166666666667	0.520833333333333	0.468085106382979	0.4	0.607954545454545
PLA017	30	20	10	40	7	0.740740740740741	0.8	0.2	0.666666666666667	0.220779220779221	0.71780303030303
PLA018	36	21	15	34	6	0.777777777777778	0.693877551020408	0.306122448979592	0.583333333333333	0.276315789473684	0.693181818181818
PLA019	42	22	20	28	5	0.814814814814815	0.583333333333333	0.416666666666667	0.523809523809524	0.333333333333333	0.653409090909091
PLA020	42	22	20	28	5	0.814814814814815	0.583333333333333	0.416666666666667	0.523809523809524	0.333333333333333	0.653409090909091
PLA021	42	22	20	28	5	0.814814814814815	0.583333333333333	0.416666666666667	0.523809523809524	0.333333333333333	0.653409090909091
PLA022	42	22	20	28	5	0.814814814814815	0.583333333333333	0.416666666666667	0.523809523809524	0.333333333333333	0.653409090909091
PLA023	47	22	25	23	5	0.814814814814815	0.479166666666667	0.520833333333333	0.468085106382979	0.4	0.607954545454545
PLA024	45	22	23	25	5	0.814814814814815	0.520833333333333	0.479166666666667	0.488888888888889	0.373333333333333	0.623106060606061
PLA025	46	23	23	24	4	0.851851851851852	0.51063829787234	0.48936170212766	0.5	0.364864864864865	0.643939393939394
PLA026	45	23	22	25	4	0.851851851851852	0.531914893617021	0.468085106382979	0.511111111111111	0.351351351351351	0.659090909090909
PLA027	40	22	18	30	5	0.814814814814815	0.625	0.375	0.55	0.306666666666667	0.683712121212121
PLA028	36	22	14	34	5	0.814814814814815	0.708333333333333	0.291666666666667	0.611111111111111	0.253333333333333	0.729166666666667
PLA029	33	21	12	37	6	0.777777777777778	0.755102040816326	0.244897959183673	0.636363636363636	0.236842105263158	0.708333333333333
PLA030	32	22	10	38	5	0.814814814814815	0.791666666666667	0.208333333333333	0.6875	0.2	0.759469696969697

Plasmodium falciparum SD01

	Total	TP	FP	TN	FN	Sensitivity	Speficity	Recall	Precision	Error rate	AUC
PLA000	47	19	28	15	7	0.730769230769231	0.348837209302326	0.651162790697674	0.404255319148936	0.507246376811594	0.463541666666667
PLA001	29	22	7	33	5	0.814814814814815	0.825	0.175	0.758620689655172	0.17910447761194	0.786458333333333
PLA002	29	22	7	33	5	0.814814814814815	0.825	0.175	0.758620689655172	0.17910447761194	0.786458333333333
PLA003	29	22	7	33	5	0.814814814814815	0.825	0.175	0.758620689655172	0.17910447761194	0.786458333333333
PLA004	29	22	7	33	5	0.814814814814815	0.825	0.175	0.758620689655172	0.17910447761194	0.786458333333333
PLA005	29	22	7	33	5	0.814814814814815	0.825	0.175	0.758620689655172	0.17910447761194	0.786458333333333
PLA006	29	22	7	33	5	0.814814814814815	0.825	0.175	0.758620689655172	0.17910447761194	0.786458333333333
PLA007	29	22	7	33	5	0.814814814814815	0.825	0.175	0.758620689655172	0.17910447761194	0.786458333333333
PLA008	29	22	7	33	5	0.814814814814815	0.825	0.175	0.758620689655172	0.17910447761194	0.786458333333333
PLA009	34	23	11	28	4	0.851851851851852	0.717948717948718	0.282051282051282	0.676470588235294	0.227272727272727	0.760416666666667
PLA010	34	23	11	28	4	0.851851851851852	0.717948717948718	0.282051282051282	0.676470588235294	0.227272727272727	0.760416666666667
PLA011	40	24	16	22	3	0.888888888888889	0.578947368421053	0.421052631578947	0.6	0.292307692307692	0.703125
PLA012	40	24	16	22	3	0.888888888888889	0.578947368421053	0.421052631578947	0.6	0.292307692307692	0.703125
PLA013	45	23	22	17	4	0.851851851851852	0.435897435897436	0.564102564102564	0.511111111111111	0.393939393939394	0.619791666666667
PLA014	44	22	22	18	5	0.814814814814815	0.45	0.55	0.5	0.402985074626866	0.598958333333333
PLA015	43	22	21	19	5	0.814814814814815	0.475	0.525	0.511627906976744	0.388059701492537	0.614583333333333
PLA016	42	22	20	20	5	0.814814814814815	0.5	0.5	0.523809523809524	0.373134328358209	0.630208333333333
PLA017	27	20	7	35	7	0.740740740740741	0.833333333333333	0.166666666666667	0.740740740740741	0.202898550724638	0.744791666666667
PLA018	31	21	10	31	6	0.777777777777778	0.75609756097561	0.24390243902439	0.67741935483871	0.235294117647059	0.734375
PLA019	36	22	14	26	5	0.814814814814815	0.65	0.35	0.611111111111111	0.283582089552239	0.692708333333333
PLA020	36	22	14	26	5	0.814814814814815	0.65	0.35	0.611111111111111	0.283582089552239	0.692708333333333
PLA021	36	22	14	26	5	0.814814814814815	0.65	0.35	0.611111111111111	0.283582089552239	0.692708333333333
PLA022	36	22	14	26	5	0.814814814814815	0.65	0.35	0.611111111111111	0.283582089552239	0.692708333333333
PLA023	41	22	19	21	5	0.814814814814815	0.525	0.475	0.536585365853659	0.358208955223881	0.645833333333333
PLA024	40	22	18	22	5	0.814814814814815	0.55	0.45	0.55	0.343283582089552	0.661458333333333
PLA025	41	23	18	21	4	0.851851851851852	0.538461538461538	0.461538461538462	0.560975609756098	0.333333333333333	0.682291666666667
PLA026	39	23	16	23	4	0.851851851851852	0.58974358974359	0.41025641025641	0.58974358974359	0.303030303030303	0.697916666666667
PLA027	34	22	12	28	5	0.814814814814815	0.7	0.3	0.647058823529412	0.253731343283582	0.723958333333333
PLA028	31	22	9	31	5	0.814814814814815	0.775	0.225	0.709677419354839	0.208955223880597	0.770833333333333
PLA029	29	21	8	33	6	0.777777777777778	0.804878048780488	0.195121951219512	0.724137931034483	0.205882352941176	0.75
PLA030	29	22	7	33	5	0.814814814814815	0.825	0.175	0.758620689655172	0.17910447761194	0.786458333333333

Plasmodium falciparum SN01

	Total	TP	FP	TN	FN	Sensitivity	Speficity	Recall	Precision	Error rate	AUC
PLA000	53	19	34	16	7	0.730769230769231	0.32	0.68	0.358490566037736	0.539473684210526	0.425595238095238
PLA001	32	22	10	37	5	0.814814814814815	0.787234042553192	0.212765957446809	0.6875	0.202702702702703	0.75297619047619
PLA002	33	22	11	36	5	0.814814814814815	0.765957446808511	0.234042553191489	0.666666666666667	0.216216216216216	0.75297619047619
PLA003	31	22	9	38	5	0.814814814814815	0.808510638297872	0.191489361702128	0.709677419354839	0.189189189189189	0.767261904761905
PLA004	32	22	10	37	5	0.814814814814815	0.787234042553192	0.212765957446809	0.6875	0.202702702702703	0.767261904761905
PLA005	31	22	9	38	5	0.814814814814815	0.808510638297872	0.191489361702128	0.709677419354839	0.189189189189189	0.767261904761905
PLA006	32	22	10	37	5	0.814814814814815	0.787234042553192	0.212765957446809	0.6875	0.202702702702703	0.767261904761905
PLA007	32	22	10	37	5	0.814814814814815	0.787234042553192	0.212765957446809	0.6875	0.202702702702703	0.75297619047619
PLA008	33	22	11	36	5	0.814814814814815	0.765957446808511	0.234042553191489	0.666666666666667	0.216216216216216	0.75297619047619
PLA009	39	23	16	30	4	0.851851851851852	0.652173913043478	0.347826086956522	0.58974358974359	0.273972602739726	0.716666666666667
PLA010	39	23	16	30	4	0.851851851851852	0.652173913043478	0.347826086956522	0.58974358974359	0.273972602739726	0.716666666666667
PLA011	45	24	21	24	3	0.888888888888889	0.533333333333333	0.466666666666667	0.533333333333333	0.333333333333333	0.666071428571429
PLA012	45	24	21	24	3	0.888888888888889	0.533333333333333	0.466666666666667	0.533333333333333	0.333333333333333	0.666071428571429
PLA013	51	23	28	18	4	0.851851851851852	0.391304347826087	0.608695652173913	0.450980392156863	0.438356164383562	0.588095238095238
PLA014	51	22	29	18	5	0.814814814814815	0.382978723404255	0.617021276595745	0.431372549019608	0.459459459459459	0.567261904761905
PLA015	49	22	27	20	5	0.814814814814815	0.425531914893617	0.574468085106383	0.448979591836735	0.432432432432432	0.581547619047619
PLA016	48	22	26	21	5	0.814814814814815	0.446808510638298	0.553191489361702	0.458333333333333	0.418918918918919	0.595833333333333
PLA017	32	20	12	37	7	0.740740740740741	0.755102040816326	0.244897959183673	0.625	0.25	0.69702380952381
PLA018	37	21	16	32	6	0.777777777777778	0.666666666666667	0.333333333333333	0.567567567567568	0.293333333333333	0.675
PLA019	40	22	18	29	5	0.814814814814815	0.617021276595745	0.382978723404255	0.55	0.310810810810811	0.667261904761905
PLA020	40	22	18	29	5	0.814814814814815	0.617021276595745	0.382978723404255	0.55	0.310810810810811	0.667261904761905
PLA021	42	22	20	27	5	0.814814814814815	0.574468085106383	0.425531914893617	0.523809523809524	0.337837837837838	0.638690476190476
PLA022	42	22	20	27	5	0.814814814814815	0.574468085106383	0.425531914893617	0.523809523809524	0.337837837837838	0.638690476190476
PLA023	47	22	25	22	5	0.814814814814815	0.468085106382979	0.531914893617021	0.468085106382979	0.405405405405405	0.624404761904762
PLA024	45	22	23	24	5	0.814814814814815	0.51063829787234	0.48936170212766	0.488888888888889	0.378378378378378	0.65297619047619
PLA025	46	23	23	23	4	0.851851851851852	0.5	0.5	0.5	0.36986301369863	0.673809523809524
PLA026	45	23	22	24	4	0.851851851851852	0.521739130434783	0.478260869565217	0.511111111111111	0.356164383561644	0.673809523809524
PLA027	38	22	16	31	5	0.814814814814815	0.659574468085106	0.340425531914894	0.578947368421053	0.283783783783784	0.695833333333333
PLA028	35	22	13	34	5	0.814814814814815	0.723404255319149	0.276595744680851	0.628571428571429	0.243243243243243	0.738690476190476
PLA029	32	21	11	37	6	0.777777777777778	0.770833333333333	0.229166666666667	0.65625	0.226666666666667	0.732142857142857
PLA030	32	22	10	37	5	0.814814814814815	0.787234042553192	0.212765957446809	0.6875	0.202702702702703	0.767261904761905

Plasmodium falciparum TG01

	Total	TP	FP	TN	FN	Sensitivity	Speficity	Recall	Precision	Error rate	AUC
PLA000	51	19	32	16	7	0.730769230769231	0.333333333333333	0.666666666666667	0.372549019607843	0.527027027027027	0.454166666666667
PLA001	30	21	9	37	6	0.777777777777778	0.804347826086957	0.195652173913043	0.7	0.205479452054795	0.741666666666667
PLA002	32	21	11	35	6	0.777777777777778	0.760869565217391	0.239130434782609	0.65625	0.232876712328767	0.741666666666667
PLA003	30	21	9	37	6	0.777777777777778	0.804347826086957	0.195652173913043	0.7	0.205479452054795	0.741666666666667
PLA004	32	21	11	35	6	0.777777777777778	0.760869565217391	0.239130434782609	0.65625	0.232876712328767	0.741666666666667
PLA005	30	21	9	37	6	0.777777777777778	0.804347826086957	0.195652173913043	0.7	0.205479452054795	0.741666666666667
PLA006	32	21	11	35	6	0.777777777777778	0.760869565217391	0.239130434782609	0.65625	0.232876712328767	0.741666666666667
PLA007	30	21	9	37	6	0.777777777777778	0.804347826086957	0.195652173913043	0.7	0.205479452054795	0.741666666666667
PLA008	32	21	11	35	6	0.777777777777778	0.760869565217391	0.239130434782609	0.65625	0.232876712328767	0.741666666666667
PLA009	37	22	15	30	5	0.814814814814815	0.666666666666667	0.333333333333333	0.594594594594595	0.277777777777778	0.7125
PLA010	37	22	15	30	5	0.814814814814815	0.666666666666667	0.333333333333333	0.594594594594595	0.277777777777778	0.7125
PLA011	44	23	21	23	4	0.851851851851852	0.522727272727273	0.477272727272727	0.522727272727273	0.352112676056338	0.666666666666667
PLA012	44	23	21	23	4	0.851851851851852	0.522727272727273	0.477272727272727	0.522727272727273	0.352112676056338	0.666666666666667
PLA013	49	22	27	18	5	0.814814814814815	0.4	0.6	0.448979591836735	0.444444444444444	0.579166666666667
PLA014	50	22	28	17	5	0.814814814814815	0.377777777777778	0.622222222222222	0.44	0.458333333333333	0.5625
PLA015	48	22	26	19	5	0.814814814814815	0.422222222222222	0.577777777777778	0.458333333333333	0.430555555555556	0.579166666666667
PLA016	47	22	25	20	5	0.814814814814815	0.444444444444444	0.555555555555556	0.468085106382979	0.416666666666667	0.595833333333333
PLA017	31	20	11	36	7	0.740740740740741	0.765957446808511	0.234042553191489	0.645161290322581	0.243243243243243	0.720833333333333
PLA018	35	21	14	32	6	0.777777777777778	0.695652173913043	0.304347826086957	0.6	0.273972602739726	0.708333333333333
PLA019	40	22	18	27	5	0.814814814814815	0.6	0.4	0.55	0.319444444444444	0.6625
PLA020	40	22	18	27	5	0.814814814814815	0.6	0.4	0.55	0.319444444444444	0.6625
PLA021	41	22	19	26	5	0.814814814814815	0.577777777777778	0.422222222222222	0.536585365853659	0.333333333333333	0.6625
PLA022	41	22	19	26	5	0.814814814814815	0.577777777777778	0.422222222222222	0.536585365853659	0.333333333333333	0.6625
PLA023	47	22	25	20	5	0.814814814814815	0.444444444444444	0.555555555555556	0.468085106382979	0.416666666666667	0.6125
PLA024	43	22	21	24	5	0.814814814814815	0.533333333333333	0.466666666666667	0.511627906976744	0.361111111111111	0.6625
PLA025	45	23	22	22	4	0.851851851851852	0.5	0.5	0.511111111111111	0.366197183098592	0.666666666666667
PLA026	44	23	21	23	4	0.851851851851852	0.522727272727273	0.477272727272727	0.522727272727273	0.352112676056338	0.666666666666667
PLA027	38	22	16	29	5	0.814814814814815	0.644444444444444	0.355555555555556	0.578947368421053	0.291666666666667	0.695833333333333
PLA028	35	22	13	32	5	0.814814814814815	0.711111111111111	0.288888888888889	0.628571428571429	0.25	0.745833333333333
PLA029	33	21	12	34	6	0.777777777777778	0.739130434782609	0.260869565217391	0.636363636363636	0.246575342465753	0.725
PLA030	32	21	11	35	6	0.777777777777778	0.760869565217391	0.239130434782609	0.65625	0.232876712328767	0.741666666666667

Plasmodium gaboni strain G01

	Total	TP	FP	TN	FN	Sensitivity	Speficity	Recall	Precision	Error rate	AUC
PLA000	46	19	27	15	6	0.76	0.357142857142857	0.642857142857143	0.41304347826087	0.492537313432836	0.504032258064516
PLA001	33	21	12	28	6	0.777777777777778	0.7	0.3	0.636363636363636	0.26865671641791	0.681451612903226
PLA002	34	21	13	27	6	0.777777777777778	0.675	0.325	0.617647058823529	0.283582089552239	0.665322580645161
PLA003	31	21	10	30	5	0.807692307692308	0.75	0.25	0.67741935483871	0.227272727272727	0.734543010752688
PLA004	35	22	13	26	5	0.814814814814815	0.666666666666667	0.333333333333333	0.628571428571429	0.272727272727273	0.686155913978495
PLA005	31	21	10	30	5	0.807692307692308	0.75	0.25	0.67741935483871	0.227272727272727	0.734543010752688
PLA006	35	22	13	26	5	0.814814814814815	0.666666666666667	0.333333333333333	0.628571428571429	0.272727272727273	0.686155913978495
PLA007	33	21	12	28	6	0.777777777777778	0.7	0.3	0.636363636363636	0.26865671641791	0.681451612903226
PLA008	34	21	13	27	6	0.777777777777778	0.675	0.325	0.617647058823529	0.283582089552239	0.665322580645161
PLA009	38	22	16	23	5	0.814814814814815	0.58974358974359	0.41025641025641	0.578947368421053	0.318181818181818	0.637768817204301
PLA010	38	22	16	23	5	0.814814814814815	0.58974358974359	0.41025641025641	0.578947368421053	0.318181818181818	0.637768817204301
PLA011	45	23	22	16	4	0.851851851851852	0.421052631578947	0.578947368421053	0.511111111111111	0.4	0.594086021505376
PLA012	45	23	22	16	4	0.851851851851852	0.421052631578947	0.578947368421053	0.511111111111111	0.4	0.594086021505376
PLA013	50	23	27	11	4	0.851851851851852	0.289473684210526	0.710526315789474	0.46	0.476923076923077	0.561827956989247
PLA014	51	22	29	10	5	0.814814814814815	0.256410256410256	0.743589743589744	0.431372549019608	0.515151515151515	0.508736559139785
PLA015	51	22	29	10	5	0.814814814814815	0.256410256410256	0.743589743589744	0.431372549019608	0.515151515151515	0.508736559139785
PLA016	50	22	28	11	5	0.814814814814815	0.282051282051282	0.717948717948718	0.44	0.5	0.524865591397849
PLA017	32	20	12	29	7	0.740740740740741	0.707317073170732	0.292682926829268	0.625	0.279411764705882	0.660618279569892
PLA018	37	21	16	24	6	0.777777777777778	0.6	0.4	0.567567567567568	0.328358208955224	0.616935483870968
PLA019	44	22	22	17	5	0.814814814814815	0.435897435897436	0.564102564102564	0.5	0.409090909090909	0.573252688172043
PLA020	44	22	22	17	5	0.814814814814815	0.435897435897436	0.564102564102564	0.5	0.409090909090909	0.573252688172043
PLA021	44	22	22	17	5	0.814814814814815	0.435897435897436	0.564102564102564	0.5	0.409090909090909	0.573252688172043
PLA022	44	22	22	17	5	0.814814814814815	0.435897435897436	0.564102564102564	0.5	0.409090909090909	0.573252688172043
PLA023	49	22	27	12	5	0.814814814814815	0.307692307692308	0.692307692307692	0.448979591836735	0.484848484848485	0.540994623655914
PLA024	47	22	25	14	5	0.814814814814815	0.358974358974359	0.641025641025641	0.468085106382979	0.454545454545455	0.573252688172043
PLA025	48	22	26	13	5	0.814814814814815	0.333333333333333	0.666666666666667	0.458333333333333	0.46969696969697	0.557123655913978
PLA026	45	23	22	16	4	0.851851851851852	0.421052631578947	0.578947368421053	0.511111111111111	0.4	0.626344086021505
PLA027	40	22	18	21	5	0.814814814814815	0.538461538461538	0.461538461538462	0.55	0.348484848484849	0.637768817204301
PLA028	33	22	11	28	5	0.814814814814815	0.717948717948718	0.282051282051282	0.666666666666667	0.242424242424242	0.718413978494624
PLA029	32	21	11	29	6	0.777777777777778	0.725	0.275	0.65625	0.253731343283582	0.69758064516129
PLA030	36	22	14	25	5	0.814814814814815	0.641025641025641	0.358974358974359	0.611111111111111	0.287878787878788	0.67002688172043

Plasmodium gallinaceum 8A

	Total	TP	FP	TN	FN	Sensitivity	Speficity	Recall	Precision	Error rate	AUC
PLA000	49	12	37	22	13	0.48	0.372881355932203	0.627118644067797	0.244897959183673	0.595238095238095	0.382227891156463
PLA001	38	15	23	33	12	0.555555555555556	0.589285714285714	0.410714285714286	0.394736842105263	0.421686746987952	0.525510204081633
PLA002	41	16	25	30	11	0.592592592592593	0.545454545454545	0.454545454545455	0.390243902439024	0.439024390243902	0.52593537414966
PLA003	38	15	23	33	12	0.555555555555556	0.589285714285714	0.410714285714286	0.394736842105263	0.421686746987952	0.525510204081633
PLA004	41	16	25	30	11	0.592592592592593	0.545454545454545	0.454545454545455	0.390243902439024	0.439024390243902	0.52593537414966
PLA005	38	15	23	33	12	0.555555555555556	0.589285714285714	0.410714285714286	0.394736842105263	0.421686746987952	0.525510204081633
PLA006	41	16	25	30	11	0.592592592592593	0.545454545454545	0.454545454545455	0.390243902439024	0.439024390243902	0.52593537414966
PLA007	38	15	23	33	12	0.555555555555556	0.589285714285714	0.410714285714286	0.394736842105263	0.421686746987952	0.525510204081633
PLA008	41	16	25	30	11	0.592592592592593	0.545454545454545	0.454545454545455	0.390243902439024	0.439024390243902	0.52593537414966
PLA009	45	17	28	26	10	0.62962962962963	0.481481481481481	0.518518518518518	0.377777777777778	0.469135802469136	0.516156462585034
PLA010	45	17	28	26	10	0.62962962962963	0.481481481481481	0.518518518518518	0.377777777777778	0.469135802469136	0.516156462585034
PLA011	48	17	31	23	10	0.62962962962963	0.425925925925926	0.574074074074074	0.354166666666667	0.506172839506173	0.485544217687075
PLA012	48	17	31	23	10	0.62962962962963	0.425925925925926	0.574074074074074	0.354166666666667	0.506172839506173	0.485544217687075
PLA013	53	17	36	18	9	0.653846153846154	0.333333333333333	0.666666666666667	0.320754716981132	0.5625	0.455357142857143
PLA014	53	17	36	18	9	0.653846153846154	0.333333333333333	0.666666666666667	0.320754716981132	0.5625	0.455357142857143
PLA015	52	16	36	19	10	0.615384615384615	0.345454545454545	0.654545454545455	0.307692307692308	0.567901234567901	0.43452380952381
PLA016	52	16	36	19	10	0.615384615384615	0.345454545454545	0.654545454545455	0.307692307692308	0.567901234567901	0.43452380952381
PLA017	38	14	24	33	12	0.538461538461538	0.578947368421053	0.421052631578947	0.368421052631579	0.433734939759036	0.505102040816327
PLA018	42	15	27	29	11	0.576923076923077	0.517857142857143	0.482142857142857	0.357142857142857	0.463414634146341	0.495323129251701
PLA019	44	14	30	27	12	0.538461538461538	0.473684210526316	0.526315789473684	0.318181818181818	0.506024096385542	0.443877551020408
PLA020	44	14	30	27	12	0.538461538461538	0.473684210526316	0.526315789473684	0.318181818181818	0.506024096385542	0.443877551020408
PLA021	45	15	30	26	11	0.576923076923077	0.464285714285714	0.535714285714286	0.333333333333333	0.5	0.464710884353741
PLA022	45	15	30	26	11	0.576923076923077	0.464285714285714	0.535714285714286	0.333333333333333	0.5	0.464710884353741
PLA023	52	17	35	19	9	0.653846153846154	0.351851851851852	0.648148148148148	0.326923076923077	0.55	0.465561224489796
PLA024	50	17	33	21	9	0.653846153846154	0.388888888888889	0.611111111111111	0.34	0.525	0.485969387755102
PLA025	52	18	34	19	9	0.666666666666667	0.358490566037736	0.641509433962264	0.346153846153846	0.5375	0.485969387755102
PLA026	54	18	36	17	9	0.666666666666667	0.320754716981132	0.679245283018868	0.333333333333333	0.5625	0.465561224489796
PLA027	45	16	29	26	11	0.592592592592593	0.472727272727273	0.527272727272727	0.355555555555556	0.48780487804878	0.485119047619048
PLA028	42	16	26	29	11	0.592592592592593	0.527272727272727	0.472727272727273	0.380952380952381	0.451219512195122	0.515731292517007
PLA029	38	16	22	33	11	0.592592592592593	0.6	0.4	0.421052631578947	0.402439024390244	0.556547619047619
PLA030	41	16	25	30	11	0.592592592592593	0.545454545454545	0.454545454545455	0.390243902439024	0.439024390243902	0.52593537414966

Plasmodium gaboni strain SY75

	Total	TP	FP	TN	FN	Sensitivity	Speficity	Recall	Precision	Error rate	AUC
PLA000	40	18	22	18	7	0.72	0.45	0.55	0.45	0.446153846153846	0.526041666666667
PLA001	32	21	11	26	6	0.777777777777778	0.702702702702703	0.297297297297297	0.65625	0.265625	0.703125
PLA002	32	21	11	26	6	0.777777777777778	0.702702702702703	0.297297297297297	0.65625	0.265625	0.703125
PLA003	33	22	11	25	5	0.814814814814815	0.694444444444444	0.305555555555556	0.666666666666667	0.253968253968254	0.723958333333333
PLA004	33	22	11	25	5	0.814814814814815	0.694444444444444	0.305555555555556	0.666666666666667	0.253968253968254	0.723958333333333
PLA005	33	22	11	25	5	0.814814814814815	0.694444444444444	0.305555555555556	0.666666666666667	0.253968253968254	0.723958333333333
PLA006	33	22	11	25	5	0.814814814814815	0.694444444444444	0.305555555555556	0.666666666666667	0.253968253968254	0.723958333333333
PLA007	32	21	11	26	6	0.777777777777778	0.702702702702703	0.297297297297297	0.65625	0.265625	0.703125
PLA008	32	21	11	26	6	0.777777777777778	0.702702702702703	0.297297297297297	0.65625	0.265625	0.703125
PLA009	35	22	13	23	5	0.814814814814815	0.638888888888889	0.361111111111111	0.628571428571429	0.285714285714286	0.692708333333333
PLA010	35	22	13	23	5	0.814814814814815	0.638888888888889	0.361111111111111	0.628571428571429	0.285714285714286	0.692708333333333
PLA011	41	23	18	17	4	0.851851851851852	0.485714285714286	0.514285714285714	0.560975609756098	0.354838709677419	0.635416666666667
PLA012	41	23	18	17	4	0.851851851851852	0.485714285714286	0.514285714285714	0.560975609756098	0.354838709677419	0.635416666666667
PLA013	43	23	20	15	4	0.851851851851852	0.428571428571429	0.571428571428571	0.534883720930233	0.387096774193548	0.619791666666667
PLA014	42	22	20	16	5	0.814814814814815	0.444444444444444	0.555555555555556	0.523809523809524	0.396825396825397	0.598958333333333
PLA015	42	22	20	16	5	0.814814814814815	0.444444444444444	0.555555555555556	0.523809523809524	0.396825396825397	0.598958333333333
PLA016	41	22	19	17	5	0.814814814814815	0.472222222222222	0.527777777777778	0.536585365853659	0.380952380952381	0.614583333333333
PLA017	28	20	8	30	7	0.740740740740741	0.789473684210526	0.210526315789474	0.714285714285714	0.230769230769231	0.729166666666667
PLA018	32	21	11	26	6	0.777777777777778	0.702702702702703	0.297297297297297	0.65625	0.265625	0.703125
PLA019	38	22	16	20	5	0.814814814814815	0.555555555555556	0.444444444444444	0.578947368421053	0.333333333333333	0.645833333333333
PLA020	38	22	16	20	5	0.814814814814815	0.555555555555556	0.444444444444444	0.578947368421053	0.333333333333333	0.645833333333333
PLA021	38	22	16	20	5	0.814814814814815	0.555555555555556	0.444444444444444	0.578947368421053	0.333333333333333	0.645833333333333
PLA022	38	22	16	20	5	0.814814814814815	0.555555555555556	0.444444444444444	0.578947368421053	0.333333333333333	0.645833333333333
PLA023	41	22	19	17	5	0.814814814814815	0.472222222222222	0.527777777777778	0.536585365853659	0.380952380952381	0.614583333333333
PLA024	39	22	17	19	5	0.814814814814815	0.527777777777778	0.472222222222222	0.564102564102564	0.349206349206349	0.645833333333333
PLA025	40	22	18	18	5	0.814814814814815	0.5	0.5	0.55	0.365079365079365	0.630208333333333
PLA026	40	23	17	18	4	0.851851851851852	0.514285714285714	0.485714285714286	0.575	0.338709677419355	0.666666666666667
PLA027	35	22	13	23	5	0.814814814814815	0.638888888888889	0.361111111111111	0.628571428571429	0.285714285714286	0.692708333333333
PLA028	31	22	9	27	5	0.814814814814815	0.75	0.25	0.709677419354839	0.222222222222222	0.755208333333333
PLA029	29	21	8	29	6	0.777777777777778	0.783783783783784	0.216216216216216	0.724137931034483	0.21875	0.75
PLA030	32	22	10	26	5	0.814814814814815	0.722222222222222	0.277777777777778	0.6875	0.238095238095238	0.739583333333333

Plasmodium knowlesi strain H

	Total	TP	FP	TN	FN	Sensitivity	Speficity	Recall	Precision	Error rate	AUC
PLA000	44	14	30	22	13	0.518518518518518	0.423076923076923	0.576923076923077	0.318181818181818	0.544303797468354	0.399621212121212
PLA001	34	17	17	32	12	0.586206896551724	0.653061224489796	0.346938775510204	0.5	0.371794871794872	0.568181818181818
PLA002	36	17	19	30	12	0.586206896551724	0.612244897959184	0.387755102040816	0.472222222222222	0.397435897435897	0.545454545454545
PLA003	36	17	19	30	12	0.586206896551724	0.612244897959184	0.387755102040816	0.472222222222222	0.397435897435897	0.545454545454545
PLA004	39	17	22	27	12	0.586206896551724	0.551020408163265	0.448979591836735	0.435897435897436	0.435897435897436	0.522727272727273
PLA005	36	17	19	30	12	0.586206896551724	0.612244897959184	0.387755102040816	0.472222222222222	0.397435897435897	0.545454545454545
PLA006	39	17	22	27	12	0.586206896551724	0.551020408163265	0.448979591836735	0.435897435897436	0.435897435897436	0.522727272727273
PLA007	34	17	17	32	12	0.586206896551724	0.653061224489796	0.346938775510204	0.5	0.371794871794872	0.568181818181818
PLA008	36	17	19	30	12	0.586206896551724	0.612244897959184	0.387755102040816	0.472222222222222	0.397435897435897	0.545454545454545
PLA009	40	18	22	26	11	0.620689655172414	0.541666666666667	0.458333333333333	0.45	0.428571428571429	0.53219696969697
PLA010	40	18	22	26	11	0.620689655172414	0.541666666666667	0.458333333333333	0.45	0.428571428571429	0.53219696969697
PLA011	42	18	24	24	11	0.620689655172414	0.5	0.5	0.428571428571429	0.454545454545455	0.509469696969697
PLA012	41	18	23	25	11	0.620689655172414	0.520833333333333	0.479166666666667	0.439024390243902	0.441558441558442	0.520833333333333
PLA013	46	18	28	20	11	0.620689655172414	0.416666666666667	0.583333333333333	0.391304347826087	0.506493506493506	0.475378787878788
PLA014	49	18	31	17	11	0.620689655172414	0.354166666666667	0.645833333333333	0.36734693877551	0.545454545454545	0.441287878787879
PLA015	47	16	31	19	11	0.592592592592593	0.38	0.62	0.340425531914894	0.545454545454545	0.441287878787879
PLA016	47	16	31	19	11	0.592592592592593	0.38	0.62	0.340425531914894	0.545454545454545	0.441287878787879
PLA017	32	15	17	34	12	0.555555555555556	0.666666666666667	0.333333333333333	0.46875	0.371794871794872	0.568181818181818
PLA018	38	16	22	28	11	0.592592592592593	0.56	0.44	0.421052631578947	0.428571428571429	0.53219696969697
PLA019	42	18	24	24	11	0.620689655172414	0.5	0.5	0.428571428571429	0.454545454545455	0.509469696969697
PLA020	42	18	24	24	11	0.620689655172414	0.5	0.5	0.428571428571429	0.454545454545455	0.509469696969697
PLA021	40	16	24	26	11	0.592592592592593	0.52	0.48	0.4	0.454545454545455	0.509469696969697
PLA022	40	16	24	26	11	0.592592592592593	0.52	0.48	0.4	0.454545454545455	0.509469696969697
PLA023	49	18	31	17	11	0.620689655172414	0.354166666666667	0.645833333333333	0.36734693877551	0.545454545454545	0.441287878787879
PLA024	48	18	30	18	11	0.620689655172414	0.375	0.625	0.375	0.532467532467532	0.452651515151515
PLA025	49	18	31	17	11	0.620689655172414	0.354166666666667	0.645833333333333	0.36734693877551	0.545454545454545	0.441287878787879
PLA026	47	18	29	19	11	0.620689655172414	0.395833333333333	0.604166666666667	0.382978723404255	0.519480519480519	0.475378787878788
PLA027	42	18	24	24	11	0.620689655172414	0.5	0.5	0.428571428571429	0.454545454545455	0.520833333333333
PLA028	40	17	23	26	12	0.586206896551724	0.530612244897959	0.469387755102041	0.425	0.448717948717949	0.511363636363636
PLA029	35	17	18	31	12	0.586206896551724	0.63265306122449	0.36734693877551	0.485714285714286	0.384615384615385	0.568181818181818
PLA030	37	17	20	29	12	0.586206896551724	0.591836734693878	0.408163265306122	0.459459459459459	0.41025641025641	0.545454545454545

Plasmodium knowlesi strain Malayan Strain Pk1 A

	Total	TP	FP	TN	FN	Sensitivity	Speficity	Recall	Precision	Error rate	AUC
PLA000	45	14	31	21	13	0.518518518518518	0.403846153846154	0.596153846153846	0.311111111111111	0.556962025316456	0.388257575757576
PLA001	34	17	17	32	12	0.586206896551724	0.653061224489796	0.346938775510204	0.5	0.371794871794872	0.568181818181818
PLA002	37	17	20	29	12	0.586206896551724	0.591836734693878	0.408163265306122	0.459459459459459	0.41025641025641	0.534090909090909
PLA003	36	17	19	30	12	0.586206896551724	0.612244897959184	0.387755102040816	0.472222222222222	0.397435897435897	0.545454545454545
PLA004	39	17	22	27	12	0.586206896551724	0.551020408163265	0.448979591836735	0.435897435897436	0.435897435897436	0.522727272727273
PLA005	36	17	19	30	12	0.586206896551724	0.612244897959184	0.387755102040816	0.472222222222222	0.397435897435897	0.545454545454545
PLA006	39	17	22	27	12	0.586206896551724	0.551020408163265	0.448979591836735	0.435897435897436	0.435897435897436	0.522727272727273
PLA007	34	17	17	32	12	0.586206896551724	0.653061224489796	0.346938775510204	0.5	0.371794871794872	0.568181818181818
PLA008	37	17	20	29	12	0.586206896551724	0.591836734693878	0.408163265306122	0.459459459459459	0.41025641025641	0.534090909090909
PLA009	41	18	23	25	11	0.620689655172414	0.520833333333333	0.479166666666667	0.439024390243902	0.441558441558442	0.520833333333333
PLA010	41	18	23	25	11	0.620689655172414	0.520833333333333	0.479166666666667	0.439024390243902	0.441558441558442	0.520833333333333
PLA011	43	18	25	23	11	0.620689655172414	0.479166666666667	0.520833333333333	0.418604651162791	0.467532467532468	0.498106060606061
PLA012	42	18	24	24	11	0.620689655172414	0.5	0.5	0.428571428571429	0.454545454545455	0.509469696969697
PLA013	47	18	29	19	11	0.620689655172414	0.395833333333333	0.604166666666667	0.382978723404255	0.519480519480519	0.464015151515151
PLA014	50	18	32	16	11	0.620689655172414	0.333333333333333	0.666666666666667	0.36	0.558441558441558	0.429924242424242
PLA015	48	16	32	18	11	0.592592592592593	0.36	0.64	0.333333333333333	0.558441558441558	0.429924242424242
PLA016	48	16	32	18	11	0.592592592592593	0.36	0.64	0.333333333333333	0.558441558441558	0.429924242424242
PLA017	33	15	18	33	12	0.555555555555556	0.647058823529412	0.352941176470588	0.454545454545455	0.384615384615385	0.556818181818182
PLA018	39	16	23	27	11	0.592592592592593	0.54	0.46	0.41025641025641	0.441558441558442	0.520833333333333
PLA019	43	18	25	23	11	0.620689655172414	0.479166666666667	0.520833333333333	0.418604651162791	0.467532467532468	0.498106060606061
PLA020	43	18	25	23	11	0.620689655172414	0.479166666666667	0.520833333333333	0.418604651162791	0.467532467532468	0.498106060606061
PLA021	41	16	25	25	11	0.592592592592593	0.5	0.5	0.390243902439024	0.467532467532468	0.498106060606061
PLA022	41	16	25	25	11	0.592592592592593	0.5	0.5	0.390243902439024	0.467532467532468	0.498106060606061
PLA023	50	18	32	16	11	0.620689655172414	0.333333333333333	0.666666666666667	0.36	0.558441558441558	0.429924242424242
PLA024	49	18	31	17	11	0.620689655172414	0.354166666666667	0.645833333333333	0.36734693877551	0.545454545454545	0.441287878787879
PLA025	50	18	32	16	11	0.620689655172414	0.333333333333333	0.666666666666667	0.36	0.558441558441558	0.429924242424242
PLA026	47	18	29	19	11	0.620689655172414	0.395833333333333	0.604166666666667	0.382978723404255	0.519480519480519	0.475378787878788
PLA027	42	18	24	24	11	0.620689655172414	0.5	0.5	0.428571428571429	0.454545454545455	0.520833333333333
PLA028	40	17	23	26	12	0.586206896551724	0.530612244897959	0.469387755102041	0.425	0.448717948717949	0.511363636363636
PLA029	35	17	18	31	12	0.586206896551724	0.63265306122449	0.36734693877551	0.485714285714286	0.384615384615385	0.568181818181818
PLA030	37	17	20	29	12	0.586206896551724	0.591836734693878	0.408163265306122	0.459459459459459	0.41025641025641	0.545454545454545

Plasmodium malariae UG01

	Total	TP	FP	TN	FN	Sensitivity	Speficity	Recall	Precision	Error rate	AUC
PLA000	63	10	53	20	14	0.416666666666667	0.273972602739726	0.726027397260274	0.158730158730159	0.690721649484536	0.295289855072464
PLA001	31	16	15	52	10	0.615384615384615	0.776119402985075	0.223880597014925	0.516129032258065	0.268817204301075	0.639492753623188
PLA002	36	16	20	47	10	0.615384615384615	0.701492537313433	0.298507462686567	0.444444444444444	0.32258064516129	0.596014492753623
PLA003	33	16	17	50	11	0.592592592592593	0.746268656716418	0.253731343283582	0.484848484848485	0.297872340425532	0.596920289855072
PLA004	37	17	20	46	10	0.62962962962963	0.696969696969697	0.303030303030303	0.459459459459459	0.32258064516129	0.596014492753623
PLA005	33	16	17	50	11	0.592592592592593	0.746268656716418	0.253731343283582	0.484848484848485	0.297872340425532	0.596920289855072
PLA006	37	17	20	46	10	0.62962962962963	0.696969696969697	0.303030303030303	0.459459459459459	0.32258064516129	0.596014492753623
PLA007	31	16	15	52	10	0.615384615384615	0.776119402985075	0.223880597014925	0.516129032258065	0.268817204301075	0.639492753623188
PLA008	36	16	20	47	10	0.615384615384615	0.701492537313433	0.298507462686567	0.444444444444444	0.32258064516129	0.596014492753623
PLA009	41	16	25	42	10	0.615384615384615	0.626865671641791	0.373134328358209	0.390243902439024	0.376344086021505	0.552536231884058
PLA010	40	16	24	43	10	0.615384615384615	0.641791044776119	0.358208955223881	0.4	0.365591397849462	0.563405797101449
PLA011	48	17	31	35	9	0.653846153846154	0.53030303030303	0.46969696969697	0.354166666666667	0.434782608695652	0.551630434782609
PLA012	47	17	30	36	9	0.653846153846154	0.545454545454545	0.454545454545455	0.361702127659574	0.423913043478261	0.5625
PLA013	57	15	42	26	10	0.6	0.382352941176471	0.617647058823529	0.263157894736842	0.559139784946237	0.465579710144928
PLA014	55	14	41	28	11	0.56	0.405797101449275	0.594202898550725	0.254545454545455	0.553191489361702	0.455615942028985
PLA015	54	14	40	29	11	0.56	0.420289855072464	0.579710144927536	0.259259259259259	0.542553191489362	0.466485507246377
PLA016	53	14	39	30	11	0.56	0.434782608695652	0.565217391304348	0.264150943396226	0.531914893617021	0.477355072463768
PLA017	32	13	19	51	12	0.52	0.728571428571429	0.271428571428571	0.40625	0.326315789473684	0.565217391304348
PLA018	34	13	21	49	12	0.52	0.7	0.3	0.382352941176471	0.347368421052632	0.554347826086957
PLA019	46	15	31	37	10	0.6	0.544117647058823	0.455882352941176	0.326086956521739	0.440860215053763	0.541666666666667
PLA020	46	15	31	37	10	0.6	0.544117647058823	0.455882352941176	0.326086956521739	0.440860215053763	0.541666666666667
PLA021	40	14	26	43	11	0.56	0.623188405797101	0.376811594202899	0.35	0.393617021276596	0.553442028985507
PLA022	40	14	26	43	11	0.56	0.623188405797101	0.376811594202899	0.35	0.393617021276596	0.553442028985507
PLA023	54	14	40	29	11	0.56	0.420289855072464	0.579710144927536	0.259259259259259	0.542553191489362	0.466485507246377
PLA024	54	14	40	29	11	0.56	0.420289855072464	0.579710144927536	0.259259259259259	0.542553191489362	0.466485507246377
PLA025	57	16	41	26	10	0.615384615384615	0.388059701492537	0.611940298507463	0.280701754385965	0.548387096774194	0.48731884057971
PLA026	57	17	40	26	10	0.62962962962963	0.393939393939394	0.606060606060606	0.298245614035088	0.537634408602151	0.519927536231884
PLA027	47	17	30	36	10	0.62962962962963	0.545454545454545	0.454545454545455	0.361702127659574	0.43010752688172	0.552536231884058
PLA028	38	16	22	45	11	0.592592592592593	0.671641791044776	0.328358208955224	0.421052631578947	0.351063829787234	0.564311594202898
PLA029	33	16	17	50	11	0.592592592592593	0.746268656716418	0.253731343283582	0.484848484848485	0.297872340425532	0.596920289855072
PLA030	36	17	19	47	10	0.62962962962963	0.712121212121212	0.287878787878788	0.472222222222222	0.311827956989247	0.596014492753623

Plasmodium ovale curtisi GH01

	Total	TP	FP	TN	FN	Sensitivity	Speficity	Recall	Precision	Error rate	AUC
PLA000	34	12	22	24	13	0.48	0.521739130434783	0.478260869565217	0.352941176470588	0.492957746478873	0.414880952380952
PLA001	31	16	15	27	9	0.64	0.642857142857143	0.357142857142857	0.516129032258065	0.358208955223881	0.6125
PLA002	36	18	18	22	7	0.72	0.55	0.45	0.5	0.384615384615385	0.611309523809524
PLA003	32	18	14	26	9	0.666666666666667	0.65	0.35	0.5625	0.343283582089552	0.626785714285714
PLA004	35	20	15	23	7	0.740740740740741	0.605263157894737	0.394736842105263	0.571428571428571	0.338461538461538	0.654166666666667
PLA005	32	18	14	26	9	0.666666666666667	0.65	0.35	0.5625	0.343283582089552	0.626785714285714
PLA006	35	20	15	23	7	0.740740740740741	0.605263157894737	0.394736842105263	0.571428571428571	0.338461538461538	0.654166666666667
PLA007	31	16	15	27	9	0.64	0.642857142857143	0.357142857142857	0.516129032258065	0.358208955223881	0.6125
PLA008	36	18	18	22	7	0.72	0.55	0.45	0.5	0.384615384615385	0.611309523809524
PLA009	41	18	23	17	7	0.72	0.425	0.575	0.439024390243902	0.461538461538462	0.554166666666667
PLA010	41	18	23	17	7	0.72	0.425	0.575	0.439024390243902	0.461538461538462	0.554166666666667
PLA011	44	19	25	14	6	0.76	0.358974358974359	0.641025641025641	0.431818181818182	0.484375	0.546428571428571
PLA012	44	19	25	14	6	0.76	0.358974358974359	0.641025641025641	0.431818181818182	0.484375	0.546428571428571
PLA013	41	18	23	17	7	0.72	0.425	0.575	0.439024390243902	0.461538461538462	0.539880952380952
PLA014	41	18	23	17	7	0.72	0.425	0.575	0.439024390243902	0.461538461538462	0.539880952380952
PLA015	39	17	22	19	8	0.68	0.463414634146341	0.536585365853659	0.435897435897436	0.454545454545455	0.533333333333333
PLA016	39	17	22	19	8	0.68	0.463414634146341	0.536585365853659	0.435897435897436	0.454545454545455	0.533333333333333
PLA017	27	16	11	31	9	0.64	0.738095238095238	0.261904761904762	0.592592592592593	0.298507462686567	0.655357142857143
PLA018	32	16	16	26	9	0.64	0.619047619047619	0.380952380952381	0.5	0.373134328358209	0.598214285714286
PLA019	38	18	20	20	7	0.72	0.5	0.5	0.473684210526316	0.415384615384615	0.59702380952381
PLA020	38	18	20	20	7	0.72	0.5	0.5	0.473684210526316	0.415384615384615	0.59702380952381
PLA021	35	17	18	23	8	0.68	0.560975609756098	0.439024390243902	0.485714285714286	0.393939393939394	0.59047619047619
PLA022	35	17	18	23	8	0.68	0.560975609756098	0.439024390243902	0.485714285714286	0.393939393939394	0.59047619047619
PLA023	40	18	22	18	7	0.72	0.45	0.55	0.45	0.446153846153846	0.554166666666667
PLA024	39	18	21	19	7	0.72	0.475	0.525	0.461538461538462	0.430769230769231	0.568452380952381
PLA025	43	19	24	15	6	0.76	0.384615384615385	0.615384615384615	0.441860465116279	0.46875	0.560714285714286
PLA026	43	20	23	15	6	0.769230769230769	0.394736842105263	0.605263157894737	0.465116279069767	0.453125	0.575
PLA027	40	20	20	18	6	0.769230769230769	0.473684210526316	0.526315789473684	0.5	0.40625	0.617857142857143
PLA028	37	19	18	21	7	0.730769230769231	0.538461538461538	0.461538461538462	0.513513513513513	0.384615384615385	0.625595238095238
PLA029	32	19	13	26	7	0.730769230769231	0.666666666666667	0.333333333333333	0.59375	0.307692307692308	0.682738095238095
PLA030	36	20	16	22	7	0.740740740740741	0.578947368421053	0.421052631578947	0.555555555555556	0.353846153846154	0.639880952380952

Plasmodium praefalciparum strain G01

	Total	TP	FP	TN	FN	Sensitivity	Speficity	Recall	Precision	Error rate	AUC
PLA000	62	19	43	15	7	0.730769230769231	0.258620689655172	0.741379310344828	0.306451612903226	0.595238095238095	0.448761261261261
PLA001	32	22	10	45	5	0.814814814814815	0.818181818181818	0.181818181818182	0.6875	0.182926829268293	0.760698198198198
PLA002	34	23	11	43	4	0.851851851851852	0.796296296296296	0.203703703703704	0.676470588235294	0.185185185185185	0.768018018018018
PLA003	32	22	10	45	5	0.814814814814815	0.818181818181818	0.181818181818182	0.6875	0.182926829268293	0.760698198198198
PLA004	34	23	11	43	4	0.851851851851852	0.796296296296296	0.203703703703704	0.676470588235294	0.185185185185185	0.768018018018018
PLA005	32	22	10	45	5	0.814814814814815	0.818181818181818	0.181818181818182	0.6875	0.182926829268293	0.760698198198198
PLA006	34	23	11	43	4	0.851851851851852	0.796296296296296	0.203703703703704	0.676470588235294	0.185185185185185	0.768018018018018
PLA007	32	22	10	45	5	0.814814814814815	0.818181818181818	0.181818181818182	0.6875	0.182926829268293	0.760698198198198
PLA008	34	23	11	43	4	0.851851851851852	0.796296296296296	0.203703703703704	0.676470588235294	0.185185185185185	0.768018018018018
PLA009	38	24	14	39	3	0.888888888888889	0.735849056603774	0.264150943396226	0.631578947368421	0.2125	0.748310810810811
PLA010	38	24	14	39	3	0.888888888888889	0.735849056603774	0.264150943396226	0.631578947368421	0.2125	0.748310810810811
PLA011	47	25	22	30	2	0.925925925925926	0.576923076923077	0.423076923076923	0.531914893617021	0.30379746835443	0.701576576576577
PLA012	47	25	22	30	2	0.925925925925926	0.576923076923077	0.423076923076923	0.531914893617021	0.30379746835443	0.701576576576577
PLA013	53	24	29	24	3	0.888888888888889	0.452830188679245	0.547169811320755	0.452830188679245	0.4	0.613175675675676
PLA014	53	23	30	24	4	0.851851851851852	0.444444444444444	0.555555555555556	0.433962264150943	0.419753086419753	0.578828828828829
PLA015	52	23	29	25	4	0.851851851851852	0.462962962962963	0.537037037037037	0.442307692307692	0.407407407407407	0.592342342342342
PLA016	49	23	26	28	4	0.851851851851852	0.518518518518518	0.481481481481481	0.469387755102041	0.37037037037037	0.619369369369369
PLA017	31	21	10	46	6	0.777777777777778	0.821428571428571	0.178571428571429	0.67741935483871	0.192771084337349	0.739864864864865
PLA018	34	22	12	43	5	0.814814814814815	0.781818181818182	0.218181818181818	0.647058823529412	0.207317073170732	0.733671171171171
PLA019	44	23	21	33	4	0.851851851851852	0.611111111111111	0.388888888888889	0.522727272727273	0.308641975308642	0.673423423423423
PLA020	44	23	21	33	4	0.851851851851852	0.611111111111111	0.388888888888889	0.522727272727273	0.308641975308642	0.673423423423423
PLA021	43	23	20	34	4	0.851851851851852	0.62962962962963	0.37037037037037	0.534883720930233	0.296296296296296	0.686936936936937
PLA022	43	23	20	34	4	0.851851851851852	0.62962962962963	0.37037037037037	0.534883720930233	0.296296296296296	0.686936936936937
PLA023	48	23	25	29	4	0.851851851851852	0.537037037037037	0.462962962962963	0.479166666666667	0.358024691358025	0.632882882882883
PLA024	46	23	23	31	4	0.851851851851852	0.574074074074074	0.425925925925926	0.5	0.333333333333333	0.65990990990991
PLA025	47	24	23	30	3	0.888888888888889	0.566037735849057	0.433962264150943	0.51063829787234	0.325	0.680743243243243
PLA026	46	24	22	31	3	0.888888888888889	0.584905660377358	0.415094339622642	0.521739130434783	0.3125	0.680743243243243
PLA027	40	23	17	37	4	0.851851851851852	0.685185185185185	0.314814814814815	0.575	0.259259259259259	0.713963963963964
PLA028	34	23	11	43	4	0.851851851851852	0.796296296296296	0.203703703703704	0.676470588235294	0.185185185185185	0.768018018018018
PLA029	33	22	11	44	5	0.814814814814815	0.8	0.2	0.666666666666667	0.195121951219512	0.747184684684685
PLA030	34	23	11	43	4	0.851851851851852	0.796296296296296	0.203703703703704	0.676470588235294	0.185185185185185	0.768018018018018

Plasmodium reichenowi CDC

	Total	TP	FP	TN	FN	Sensitivity	Speficity	Recall	Precision	Error rate	AUC
PLA000	52	21	31	12	5	0.807692307692308	0.27906976744186	0.720930232558139	0.403846153846154	0.521739130434783	0.498774509803922
PLA001	34	22	12	30	5	0.814814814814815	0.714285714285714	0.285714285714286	0.647058823529412	0.246376811594203	0.719362745098039
PLA002	34	22	12	30	5	0.814814814814815	0.714285714285714	0.285714285714286	0.647058823529412	0.246376811594203	0.719362745098039
PLA003	33	21	12	31	6	0.777777777777778	0.720930232558139	0.27906976744186	0.636363636363636	0.257142857142857	0.698529411764706
PLA004	33	21	12	31	6	0.777777777777778	0.720930232558139	0.27906976744186	0.636363636363636	0.257142857142857	0.698529411764706
PLA005	33	21	12	31	6	0.777777777777778	0.720930232558139	0.27906976744186	0.636363636363636	0.257142857142857	0.698529411764706
PLA006	33	21	12	31	6	0.777777777777778	0.720930232558139	0.27906976744186	0.636363636363636	0.257142857142857	0.698529411764706
PLA007	34	22	12	30	5	0.814814814814815	0.714285714285714	0.285714285714286	0.647058823529412	0.246376811594203	0.719362745098039
PLA008	34	22	12	30	5	0.814814814814815	0.714285714285714	0.285714285714286	0.647058823529412	0.246376811594203	0.719362745098039
PLA009	40	23	17	24	4	0.851851851851852	0.585365853658537	0.414634146341463	0.575	0.308823529411765	0.666666666666667
PLA010	40	23	17	24	4	0.851851851851852	0.585365853658537	0.414634146341463	0.575	0.308823529411765	0.666666666666667
PLA011	44	24	20	20	3	0.888888888888889	0.5	0.5	0.545454545454545	0.343283582089552	0.643382352941176
PLA012	44	24	20	20	3	0.888888888888889	0.5	0.5	0.545454545454545	0.343283582089552	0.643382352941176
PLA013	47	23	24	17	4	0.851851851851852	0.414634146341463	0.585365853658537	0.48936170212766	0.411764705882353	0.57843137254902
PLA014	48	23	25	16	4	0.851851851851852	0.390243902439024	0.609756097560976	0.479166666666667	0.426470588235294	0.563725490196078
PLA015	47	23	24	17	4	0.851851851851852	0.414634146341463	0.585365853658537	0.48936170212766	0.411764705882353	0.57843137254902
PLA016	46	23	23	18	4	0.851851851851852	0.439024390243902	0.560975609756098	0.5	0.397058823529412	0.593137254901961
PLA017	33	21	12	31	6	0.777777777777778	0.720930232558139	0.27906976744186	0.636363636363636	0.257142857142857	0.698529411764706
PLA018	38	22	16	26	5	0.814814814814815	0.619047619047619	0.380952380952381	0.578947368421053	0.304347826086957	0.660539215686274
PLA019	39	22	17	25	5	0.814814814814815	0.595238095238095	0.404761904761905	0.564102564102564	0.318840579710145	0.645833333333333
PLA020	39	22	17	25	5	0.814814814814815	0.595238095238095	0.404761904761905	0.564102564102564	0.318840579710145	0.645833333333333
PLA021	42	23	19	22	4	0.851851851851852	0.536585365853659	0.463414634146341	0.547619047619048	0.338235294117647	0.637254901960784
PLA022	42	23	19	22	4	0.851851851851852	0.536585365853659	0.463414634146341	0.547619047619048	0.338235294117647	0.637254901960784
PLA023	44	23	21	20	4	0.851851851851852	0.48780487804878	0.51219512195122	0.522727272727273	0.367647058823529	0.622549019607843
PLA024	42	23	19	22	4	0.851851851851852	0.536585365853659	0.463414634146341	0.547619047619048	0.338235294117647	0.651960784313726
PLA025	43	24	19	21	3	0.888888888888889	0.525	0.475	0.558139534883721	0.328358208955224	0.672794117647059
PLA026	41	23	18	23	4	0.851851851851852	0.560975609756098	0.439024390243902	0.560975609756098	0.323529411764706	0.666666666666667
PLA027	37	22	15	27	5	0.814814814814815	0.642857142857143	0.357142857142857	0.594594594594595	0.289855072463768	0.675245098039216
PLA028	33	22	11	31	5	0.814814814814815	0.738095238095238	0.261904761904762	0.666666666666667	0.231884057971014	0.73406862745098
PLA029	32	21	11	32	6	0.777777777777778	0.744186046511628	0.255813953488372	0.65625	0.242857142857143	0.713235294117647
PLA030	33	21	12	31	6	0.777777777777778	0.720930232558139	0.27906976744186	0.636363636363636	0.257142857142857	0.698529411764706

Plasmodium relictum SGS1−like

	Total	TP	FP	TN	FN	Sensitivity	Speficity	Recall	Precision	Error rate	AUC
PLA000	47	15	32	22	11	0.576923076923077	0.407407407407407	0.592592592592593	0.319148936170213	0.5375	0.444746376811594
PLA001	36	18	18	33	10	0.642857142857143	0.647058823529412	0.352941176470588	0.5	0.354430379746835	0.596014492753623
PLA002	37	18	19	32	10	0.642857142857143	0.627450980392157	0.372549019607843	0.486486486486487	0.367088607594937	0.585144927536232
PLA003	37	18	19	32	10	0.642857142857143	0.627450980392157	0.372549019607843	0.486486486486487	0.367088607594937	0.585144927536232
PLA004	38	18	20	31	10	0.642857142857143	0.607843137254902	0.392156862745098	0.473684210526316	0.379746835443038	0.574275362318841
PLA005	37	18	19	32	10	0.642857142857143	0.627450980392157	0.372549019607843	0.486486486486487	0.367088607594937	0.585144927536232
PLA006	38	18	20	31	10	0.642857142857143	0.607843137254902	0.392156862745098	0.473684210526316	0.379746835443038	0.574275362318841
PLA007	36	18	18	33	10	0.642857142857143	0.647058823529412	0.352941176470588	0.5	0.354430379746835	0.596014492753623
PLA008	37	18	19	32	10	0.642857142857143	0.627450980392157	0.372549019607843	0.486486486486487	0.367088607594937	0.585144927536232
PLA009	41	18	23	28	10	0.642857142857143	0.549019607843137	0.450980392156863	0.439024390243902	0.417721518987342	0.541666666666667
PLA010	40	18	22	29	10	0.642857142857143	0.568627450980392	0.431372549019608	0.45	0.40506329113924	0.552536231884058
PLA011	46	20	26	23	8	0.714285714285714	0.469387755102041	0.530612244897959	0.434782608695652	0.441558441558442	0.550724637681159
PLA012	45	20	25	24	8	0.714285714285714	0.489795918367347	0.510204081632653	0.444444444444444	0.428571428571429	0.561594202898551
PLA013	54	19	35	15	8	0.703703703703704	0.3	0.7	0.351851851851852	0.558441558441558	0.463768115942029
PLA014	53	19	34	16	8	0.703703703703704	0.32	0.68	0.358490566037736	0.545454545454545	0.47463768115942
PLA015	51	18	33	18	8	0.692307692307692	0.352941176470588	0.647058823529412	0.352941176470588	0.532467532467532	0.485507246376812
PLA016	50	18	32	19	8	0.692307692307692	0.372549019607843	0.627450980392157	0.36	0.519480519480519	0.496376811594203
PLA017	33	15	18	36	11	0.576923076923077	0.666666666666667	0.333333333333333	0.454545454545455	0.3625	0.57518115942029
PLA018	36	15	21	33	11	0.576923076923077	0.611111111111111	0.388888888888889	0.416666666666667	0.4	0.542572463768116
PLA019	47	18	29	22	9	0.666666666666667	0.431372549019608	0.568627450980392	0.382978723404255	0.487179487179487	0.497282608695652
PLA020	47	18	29	22	9	0.666666666666667	0.431372549019608	0.568627450980392	0.382978723404255	0.487179487179487	0.497282608695652
PLA021	42	17	25	27	9	0.653846153846154	0.519230769230769	0.480769230769231	0.404761904761905	0.435897435897436	0.540760869565217
PLA022	42	17	25	27	9	0.653846153846154	0.519230769230769	0.480769230769231	0.404761904761905	0.435897435897436	0.540760869565217
PLA023	51	19	32	18	8	0.703703703703704	0.36	0.64	0.372549019607843	0.519480519480519	0.496376811594203
PLA024	50	19	31	19	8	0.703703703703704	0.38	0.62	0.38	0.506493506493506	0.507246376811594
PLA025	52	21	31	17	7	0.75	0.354166666666667	0.645833333333333	0.403846153846154	0.5	0.528079710144928
PLA026	54	20	34	15	8	0.714285714285714	0.306122448979592	0.693877551020408	0.37037037037037	0.545454545454545	0.47463768115942
PLA027	46	19	27	23	9	0.678571428571429	0.46	0.54	0.41304347826087	0.461538461538462	0.519021739130435
PLA028	39	17	22	30	11	0.607142857142857	0.576923076923077	0.423076923076923	0.435897435897436	0.4125	0.531702898550725
PLA029	37	17	20	32	11	0.607142857142857	0.615384615384615	0.384615384615385	0.459459459459459	0.3875	0.553442028985507
PLA030	38	18	20	31	10	0.642857142857143	0.607843137254902	0.392156862745098	0.473684210526316	0.379746835443038	0.574275362318841

Plasmodium reichenowi G01

	Total	TP	FP	TN	FN	Sensitivity	Speficity	Recall	Precision	Error rate	AUC
PLA000	57	21	36	13	5	0.807692307692308	0.26530612244898	0.73469387755102	0.368421052631579	0.546666666666667	0.486742424242424
PLA001	35	22	13	35	5	0.814814814814815	0.729166666666667	0.270833333333333	0.628571428571429	0.24	0.698863636363636
PLA002	35	22	13	35	5	0.814814814814815	0.729166666666667	0.270833333333333	0.628571428571429	0.24	0.698863636363636
PLA003	34	22	12	36	5	0.814814814814815	0.75	0.25	0.647058823529412	0.226666666666667	0.714015151515151
PLA004	34	22	12	36	5	0.814814814814815	0.75	0.25	0.647058823529412	0.226666666666667	0.714015151515151
PLA005	34	22	12	36	5	0.814814814814815	0.75	0.25	0.647058823529412	0.226666666666667	0.714015151515151
PLA006	34	22	12	36	5	0.814814814814815	0.75	0.25	0.647058823529412	0.226666666666667	0.714015151515151
PLA007	35	22	13	35	5	0.814814814814815	0.729166666666667	0.270833333333333	0.628571428571429	0.24	0.698863636363636
PLA008	35	22	13	35	5	0.814814814814815	0.729166666666667	0.270833333333333	0.628571428571429	0.24	0.698863636363636
PLA009	40	23	17	30	4	0.851851851851852	0.638297872340426	0.361702127659574	0.575	0.283783783783784	0.659090909090909
PLA010	40	23	17	30	4	0.851851851851852	0.638297872340426	0.361702127659574	0.575	0.283783783783784	0.659090909090909
PLA011	46	24	22	24	3	0.888888888888889	0.521739130434783	0.478260869565217	0.521739130434783	0.342465753424658	0.619318181818182
PLA012	46	24	22	24	3	0.888888888888889	0.521739130434783	0.478260869565217	0.521739130434783	0.342465753424658	0.619318181818182
PLA013	50	23	27	20	4	0.851851851851852	0.425531914893617	0.574468085106383	0.46	0.418918918918919	0.568181818181818
PLA014	51	23	28	19	4	0.851851851851852	0.404255319148936	0.595744680851064	0.450980392156863	0.432432432432432	0.553030303030303
PLA015	50	23	27	20	4	0.851851851851852	0.425531914893617	0.574468085106383	0.46	0.418918918918919	0.568181818181818
PLA016	48	23	25	22	4	0.851851851851852	0.468085106382979	0.531914893617021	0.479166666666667	0.391891891891892	0.598484848484849
PLA017	32	21	11	38	6	0.777777777777778	0.775510204081633	0.224489795918367	0.65625	0.223684210526316	0.708333333333333
PLA018	36	22	14	34	5	0.814814814814815	0.708333333333333	0.291666666666667	0.611111111111111	0.253333333333333	0.683712121212121
PLA019	39	22	17	31	5	0.814814814814815	0.645833333333333	0.354166666666667	0.564102564102564	0.293333333333333	0.638257575757576
PLA020	39	22	17	31	5	0.814814814814815	0.645833333333333	0.354166666666667	0.564102564102564	0.293333333333333	0.638257575757576
PLA021	41	23	18	29	4	0.851851851851852	0.617021276595745	0.382978723404255	0.560975609756098	0.297297297297297	0.643939393939394
PLA022	41	23	18	29	4	0.851851851851852	0.617021276595745	0.382978723404255	0.560975609756098	0.297297297297297	0.643939393939394
PLA023	47	23	24	23	4	0.851851851851852	0.48936170212766	0.51063829787234	0.48936170212766	0.378378378378378	0.613636363636364
PLA024	45	23	22	25	4	0.851851851851852	0.531914893617021	0.468085106382979	0.511111111111111	0.351351351351351	0.643939393939394
PLA025	47	24	23	23	3	0.888888888888889	0.5	0.5	0.51063829787234	0.356164383561644	0.649621212121212
PLA026	45	24	21	25	3	0.888888888888889	0.543478260869565	0.456521739130435	0.533333333333333	0.328767123287671	0.679924242424242
PLA027	38	23	15	32	4	0.851851851851852	0.680851063829787	0.319148936170213	0.605263157894737	0.256756756756757	0.689393939393939
PLA028	33	23	10	37	4	0.851851851851852	0.787234042553192	0.212765957446809	0.696969696969697	0.189189189189189	0.765151515151515
PLA029	32	22	10	38	5	0.814814814814815	0.791666666666667	0.208333333333333	0.6875	0.2	0.744318181818182
PLA030	33	22	11	37	5	0.814814814814815	0.770833333333333	0.229166666666667	0.666666666666667	0.213333333333333	0.729166666666667

Plasmodium vivax−like sp. Pvl01

	Total	TP	FP	TN	FN	Sensitivity	Speficity	Recall	Precision	Error rate	AUC
PLA000	37	7	30	20	17	0.291666666666667	0.4	0.6	0.189189189189189	0.635135135135135	0.251893939393939
PLA001	22	13	9	35	12	0.52	0.795454545454545	0.204545454545455	0.590909090909091	0.304347826086957	0.613636363636364
PLA002	25	14	11	32	11	0.56	0.744186046511628	0.255813953488372	0.56	0.323529411764706	0.604166666666667
PLA003	25	16	9	32	11	0.592592592592593	0.780487804878049	0.219512195121951	0.64	0.294117647058824	0.634469696969697
PLA004	26	16	10	31	11	0.592592592592593	0.75609756097561	0.24390243902439	0.615384615384615	0.308823529411765	0.619318181818182
PLA005	25	16	9	32	11	0.592592592592593	0.780487804878049	0.219512195121951	0.64	0.294117647058824	0.634469696969697
PLA006	26	16	10	31	11	0.592592592592593	0.75609756097561	0.24390243902439	0.615384615384615	0.308823529411765	0.619318181818182
PLA007	22	13	9	35	12	0.52	0.795454545454545	0.204545454545455	0.590909090909091	0.304347826086957	0.613636363636364
PLA008	25	14	11	32	11	0.56	0.744186046511628	0.255813953488372	0.56	0.323529411764706	0.604166666666667
PLA009	32	17	15	25	9	0.653846153846154	0.625	0.375	0.53125	0.363636363636364	0.600378787878788
PLA010	32	17	15	25	9	0.653846153846154	0.625	0.375	0.53125	0.363636363636364	0.600378787878788
PLA011	35	17	18	22	9	0.653846153846154	0.55	0.45	0.485714285714286	0.409090909090909	0.570075757575758
PLA012	35	17	18	22	9	0.653846153846154	0.55	0.45	0.485714285714286	0.409090909090909	0.570075757575758
PLA013	42	16	26	15	10	0.615384615384615	0.365853658536585	0.634146341463415	0.380952380952381	0.537313432835821	0.443181818181818
PLA014	41	16	25	16	10	0.615384615384615	0.390243902439024	0.609756097560976	0.390243902439024	0.522388059701492	0.458333333333333
PLA015	40	15	25	17	11	0.576923076923077	0.404761904761905	0.595238095238095	0.375	0.529411764705882	0.4375
PLA016	39	15	24	18	11	0.576923076923077	0.428571428571429	0.571428571428571	0.384615384615385	0.514705882352941	0.452651515151515
PLA017	22	12	10	35	13	0.48	0.777777777777778	0.222222222222222	0.545454545454545	0.328571428571429	0.577651515151515
PLA018	29	15	14	28	11	0.576923076923077	0.666666666666667	0.333333333333333	0.517241379310345	0.367647058823529	0.573863636363636
PLA019	28	15	13	29	11	0.576923076923077	0.69047619047619	0.30952380952381	0.535714285714286	0.352941176470588	0.589015151515151
PLA020	28	15	13	29	11	0.576923076923077	0.69047619047619	0.30952380952381	0.535714285714286	0.352941176470588	0.589015151515151
PLA021	31	15	16	26	11	0.576923076923077	0.619047619047619	0.380952380952381	0.483870967741935	0.397058823529412	0.543560606060606
PLA022	31	15	16	26	11	0.576923076923077	0.619047619047619	0.380952380952381	0.483870967741935	0.397058823529412	0.543560606060606
PLA023	38	15	23	19	11	0.576923076923077	0.452380952380952	0.547619047619048	0.394736842105263	0.5	0.46780303030303
PLA024	36	15	21	21	11	0.576923076923077	0.5	0.5	0.416666666666667	0.470588235294118	0.498106060606061
PLA025	38	16	22	19	10	0.615384615384615	0.463414634146341	0.536585365853659	0.421052631578947	0.477611940298507	0.503787878787879
PLA026	38	18	20	19	10	0.642857142857143	0.487179487179487	0.512820512820513	0.473684210526316	0.447761194029851	0.534090909090909
PLA027	31	18	13	26	10	0.642857142857143	0.666666666666667	0.333333333333333	0.580645161290323	0.343283582089552	0.609848484848485
PLA028	27	15	12	30	12	0.555555555555556	0.714285714285714	0.285714285714286	0.555555555555556	0.347826086956522	0.583333333333333
PLA029	24	15	9	33	12	0.555555555555556	0.785714285714286	0.214285714285714	0.625	0.304347826086957	0.613636363636364
PLA030	26	16	10	31	11	0.592592592592593	0.75609756097561	0.24390243902439	0.615384615384615	0.308823529411765	0.619318181818182

Plasmodium vivax P01

	Total	TP	FP	TN	FN	Sensitivity	Speficity	Recall	Precision	Error rate	AUC
PLA000	41	13	28	26	14	0.481481481481481	0.481481481481481	0.518518518518518	0.317073170731707	0.518518518518518	0.412878787878788
PLA001	30	16	14	37	10	0.615384615384615	0.725490196078431	0.274509803921569	0.533333333333333	0.311688311688312	0.632575757575758
PLA002	33	16	17	34	10	0.615384615384615	0.666666666666667	0.333333333333333	0.484848484848485	0.350649350649351	0.609848484848485
PLA003	33	18	15	34	10	0.642857142857143	0.693877551020408	0.306122448979592	0.545454545454545	0.324675324675325	0.621212121212121
PLA004	35	18	17	32	10	0.642857142857143	0.653061224489796	0.346938775510204	0.514285714285714	0.350649350649351	0.609848484848485
PLA005	33	18	15	34	10	0.642857142857143	0.693877551020408	0.306122448979592	0.545454545454545	0.324675324675325	0.621212121212121
PLA006	35	18	17	32	10	0.642857142857143	0.653061224489796	0.346938775510204	0.514285714285714	0.350649350649351	0.609848484848485
PLA007	30	16	14	37	10	0.615384615384615	0.725490196078431	0.274509803921569	0.533333333333333	0.311688311688312	0.632575757575758
PLA008	33	16	17	34	10	0.615384615384615	0.666666666666667	0.333333333333333	0.484848484848485	0.350649350649351	0.609848484848485
PLA009	40	17	23	27	9	0.653846153846154	0.54	0.46	0.425	0.421052631578947	0.5625
PLA010	40	17	23	27	9	0.653846153846154	0.54	0.46	0.425	0.421052631578947	0.5625
PLA011	43	18	25	24	8	0.692307692307692	0.489795918367347	0.510204081632653	0.418604651162791	0.44	0.560606060606061
PLA012	43	18	25	24	8	0.692307692307692	0.489795918367347	0.510204081632653	0.418604651162791	0.44	0.560606060606061
PLA013	51	17	34	16	9	0.653846153846154	0.32	0.68	0.333333333333333	0.565789473684211	0.448863636363636
PLA014	51	17	34	16	9	0.653846153846154	0.32	0.68	0.333333333333333	0.565789473684211	0.448863636363636
PLA015	48	15	33	19	11	0.576923076923077	0.365384615384615	0.634615384615385	0.3125	0.564102564102564	0.418560606060606
PLA016	48	15	33	19	11	0.576923076923077	0.365384615384615	0.634615384615385	0.3125	0.564102564102564	0.418560606060606
PLA017	30	14	16	37	12	0.538461538461538	0.69811320754717	0.30188679245283	0.466666666666667	0.354430379746835	0.579545454545455
PLA018	35	15	20	32	11	0.576923076923077	0.615384615384615	0.384615384615385	0.428571428571429	0.397435897435897	0.554924242424242
PLA019	39	17	22	28	10	0.62962962962963	0.56	0.44	0.435897435897436	0.415584415584416	0.553030303030303
PLA020	39	17	22	28	10	0.62962962962963	0.56	0.44	0.435897435897436	0.415584415584416	0.553030303030303
PLA021	37	15	22	30	11	0.576923076923077	0.576923076923077	0.423076923076923	0.405405405405405	0.423076923076923	0.53219696969697
PLA022	37	15	22	30	11	0.576923076923077	0.576923076923077	0.423076923076923	0.405405405405405	0.423076923076923	0.53219696969697
PLA023	50	16	34	17	10	0.615384615384615	0.333333333333333	0.666666666666667	0.32	0.571428571428571	0.428030303030303
PLA024	46	15	31	21	11	0.576923076923077	0.403846153846154	0.596153846153846	0.326086956521739	0.538461538461538	0.441287878787879
PLA025	48	16	32	19	10	0.615384615384615	0.372549019607843	0.627450980392157	0.333333333333333	0.545454545454545	0.450757575757576
PLA026	47	17	30	20	10	0.62962962962963	0.4	0.6	0.361702127659574	0.519480519480519	0.473484848484849
PLA027	39	17	22	28	10	0.62962962962963	0.56	0.44	0.435897435897436	0.415584415584416	0.553030303030303
PLA028	35	15	20	32	12	0.555555555555556	0.615384615384615	0.384615384615385	0.428571428571429	0.40506329113924	0.534090909090909
PLA029	32	15	17	35	12	0.555555555555556	0.673076923076923	0.326923076923077	0.46875	0.367088607594937	0.568181818181818
PLA030	34	17	17	33	10	0.62962962962963	0.66	0.34	0.5	0.350649350649351	0.609848484848485

Plasmodium vivax Sal−1

	Total	TP	FP	TN	FN	Sensitivity	Speficity	Recall	Precision	Error rate	AUC
PLA000	38	13	25	23	13	0.5	0.479166666666667	0.520833333333333	0.342105263157895	0.513513513513513	0.413377192982456
PLA001	33	19	14	28	9	0.678571428571429	0.666666666666667	0.333333333333333	0.575757575757576	0.328571428571429	0.62828947368421
PLA002	35	19	16	26	9	0.678571428571429	0.619047619047619	0.380952380952381	0.542857142857143	0.357142857142857	0.615131578947368
PLA003	33	19	14	28	9	0.678571428571429	0.666666666666667	0.333333333333333	0.575757575757576	0.328571428571429	0.62828947368421
PLA004	34	19	15	27	9	0.678571428571429	0.642857142857143	0.357142857142857	0.558823529411765	0.342857142857143	0.62828947368421
PLA005	33	19	14	28	9	0.678571428571429	0.666666666666667	0.333333333333333	0.575757575757576	0.328571428571429	0.62828947368421
PLA006	34	19	15	27	9	0.678571428571429	0.642857142857143	0.357142857142857	0.558823529411765	0.342857142857143	0.62828947368421
PLA007	33	19	14	28	9	0.678571428571429	0.666666666666667	0.333333333333333	0.575757575757576	0.328571428571429	0.62828947368421
PLA008	35	19	16	26	9	0.678571428571429	0.619047619047619	0.380952380952381	0.542857142857143	0.357142857142857	0.615131578947368
PLA009	41	20	21	20	8	0.714285714285714	0.48780487804878	0.51219512195122	0.48780487804878	0.420289855072464	0.570175438596491
PLA010	41	20	21	20	8	0.714285714285714	0.48780487804878	0.51219512195122	0.48780487804878	0.420289855072464	0.570175438596491
PLA011	44	21	23	17	7	0.75	0.425	0.575	0.477272727272727	0.441176470588235	0.56469298245614
PLA012	44	21	23	17	7	0.75	0.425	0.575	0.477272727272727	0.441176470588235	0.56469298245614
PLA013	48	19	29	13	8	0.703703703703704	0.30952380952381	0.69047619047619	0.395833333333333	0.536231884057971	0.478070175438596
PLA014	47	18	29	14	9	0.666666666666667	0.325581395348837	0.674418604651163	0.382978723404255	0.542857142857143	0.457236842105263
PLA015	45	16	29	16	11	0.592592592592593	0.355555555555556	0.644444444444444	0.355555555555556	0.555555555555556	0.415570175438596
PLA016	45	16	29	16	11	0.592592592592593	0.355555555555556	0.644444444444444	0.355555555555556	0.555555555555556	0.415570175438596
PLA017	30	15	15	31	12	0.555555555555556	0.673913043478261	0.326086956521739	0.5	0.36986301369863	0.565789473684211
PLA018	35	16	19	26	11	0.592592592592593	0.577777777777778	0.422222222222222	0.457142857142857	0.416666666666667	0.533991228070176
PLA019	36	17	19	25	10	0.62962962962963	0.568181818181818	0.431818181818182	0.472222222222222	0.408450704225352	0.554824561403509
PLA020	36	17	19	25	10	0.62962962962963	0.568181818181818	0.431818181818182	0.472222222222222	0.408450704225352	0.554824561403509
PLA021	37	16	21	24	11	0.592592592592593	0.533333333333333	0.466666666666667	0.432432432432432	0.444444444444444	0.507675438596491
PLA022	37	16	21	24	11	0.592592592592593	0.533333333333333	0.466666666666667	0.432432432432432	0.444444444444444	0.507675438596491
PLA023	46	17	29	15	10	0.62962962962963	0.340909090909091	0.659090909090909	0.369565217391304	0.549295774647887	0.43640350877193
PLA024	43	17	26	18	10	0.62962962962963	0.409090909090909	0.590909090909091	0.395348837209302	0.507042253521127	0.475877192982456
PLA025	46	19	27	15	9	0.678571428571429	0.357142857142857	0.642857142857143	0.41304347826087	0.514285714285714	0.483552631578947
PLA026	44	19	25	17	9	0.678571428571429	0.404761904761905	0.595238095238095	0.431818181818182	0.485714285714286	0.509868421052632
PLA027	38	19	19	23	9	0.678571428571429	0.547619047619048	0.452380952380952	0.5	0.4	0.575657894736842
PLA028	34	17	17	27	11	0.607142857142857	0.613636363636364	0.386363636363636	0.5	0.388888888888889	0.56030701754386
PLA029	32	17	15	29	11	0.607142857142857	0.659090909090909	0.340909090909091	0.53125	0.361111111111111	0.586622807017544
PLA030	34	19	15	27	9	0.678571428571429	0.642857142857143	0.357142857142857	0.558823529411765	0.342857142857143	0.62828947368421

Plasmodium yoelii yoelii 17XNL

	Total	TP	FP	TN	FN	Sensitivity	Speficity	Recall	Precision	Error rate	AUC
PLA000	38	5	33	60	20	0.2	0.645161290322581	0.354838709677419	0.131578947368421	0.449152542372881	0.390562248995984
PLA001	42	10	32	56	16	0.384615384615385	0.636363636363636	0.363636363636364	0.238095238095238	0.421052631578947	0.485943775100402
PLA002	46	10	36	52	16	0.384615384615385	0.590909090909091	0.409090909090909	0.217391304347826	0.456140350877193	0.461847389558233
PLA003	40	9	31	58	17	0.346153846153846	0.651685393258427	0.348314606741573	0.225	0.417391304347826	0.47113453815261
PLA004	44	9	35	54	17	0.346153846153846	0.606741573033708	0.393258426966292	0.204545454545455	0.452173913043478	0.4410140562249
PLA005	40	9	31	58	17	0.346153846153846	0.651685393258427	0.348314606741573	0.225	0.417391304347826	0.47113453815261
PLA006	44	9	35	54	17	0.346153846153846	0.606741573033708	0.393258426966292	0.204545454545455	0.452173913043478	0.4410140562249
PLA007	42	10	32	56	16	0.384615384615385	0.636363636363636	0.363636363636364	0.238095238095238	0.421052631578947	0.485943775100402
PLA008	46	10	36	52	16	0.384615384615385	0.590909090909091	0.409090909090909	0.217391304347826	0.456140350877193	0.461847389558233
PLA009	54	10	44	44	16	0.384615384615385	0.5	0.5	0.185185185185185	0.526315789473684	0.419678714859438
PLA010	54	10	44	44	16	0.384615384615385	0.5	0.5	0.185185185185185	0.526315789473684	0.419678714859438
PLA011	60	10	50	38	16	0.384615384615385	0.431818181818182	0.568181818181818	0.166666666666667	0.578947368421053	0.383534136546185
PLA012	60	10	50	38	16	0.384615384615385	0.431818181818182	0.568181818181818	0.166666666666667	0.578947368421053	0.383534136546185
PLA013	68	10	58	30	16	0.384615384615385	0.340909090909091	0.659090909090909	0.147058823529412	0.649122807017544	0.34136546184739
PLA014	66	10	56	32	16	0.384615384615385	0.363636363636364	0.636363636363636	0.151515151515152	0.631578947368421	0.353413654618474
PLA015	61	10	51	37	16	0.384615384615385	0.420454545454545	0.579545454545455	0.163934426229508	0.587719298245614	0.377510040160643
PLA016	59	10	49	39	16	0.384615384615385	0.443181818181818	0.556818181818182	0.169491525423729	0.570175438596491	0.389558232931727
PLA017	38	10	28	60	16	0.384615384615385	0.681818181818182	0.318181818181818	0.263157894736842	0.385964912280702	0.504016064257028
PLA018	46	10	36	52	16	0.384615384615385	0.590909090909091	0.409090909090909	0.217391304347826	0.456140350877193	0.461847389558233
PLA019	56	10	46	42	16	0.384615384615385	0.477272727272727	0.522727272727273	0.178571428571429	0.543859649122807	0.413654618473896
PLA020	56	10	46	42	16	0.384615384615385	0.477272727272727	0.522727272727273	0.178571428571429	0.543859649122807	0.413654618473896
PLA021	52	10	42	46	16	0.384615384615385	0.522727272727273	0.477272727272727	0.192307692307692	0.508771929824561	0.42570281124498
PLA022	52	10	42	46	16	0.384615384615385	0.522727272727273	0.477272727272727	0.192307692307692	0.508771929824561	0.42570281124498
PLA023	63	9	54	35	17	0.346153846153846	0.393258426966292	0.606741573033708	0.142857142857143	0.617391304347826	0.344628514056225
PLA024	62	9	53	36	17	0.346153846153846	0.404494382022472	0.595505617977528	0.145161290322581	0.608695652173913	0.350652610441767
PLA025	65	9	56	33	17	0.346153846153846	0.370786516853933	0.629213483146067	0.138461538461538	0.634782608695652	0.332580321285141
PLA026	75	9	66	23	17	0.346153846153846	0.258426966292135	0.741573033707865	0.12	0.721739130434783	0.278363453815261
PLA027	62	9	53	36	17	0.346153846153846	0.404494382022472	0.595505617977528	0.145161290322581	0.608695652173913	0.350652610441767
PLA028	55	9	46	43	17	0.346153846153846	0.48314606741573	0.51685393258427	0.163636363636364	0.547826086956522	0.392821285140562
PLA029	44	8	36	54	18	0.307692307692308	0.6	0.4	0.181818181818182	0.46551724137931	0.426204819277108
PLA030	46	9	37	52	17	0.346153846153846	0.584269662921348	0.415730337078652	0.195652173913043	0.469565217391304	0.428965863453815

Plasmodium yoelii yoelii 17X

	Total	TP	FP	TN	FN	Sensitivity	Speficity	Recall	Precision	Error rate	AUC
PLA000	42	11	31	29	14	0.44	0.483333333333333	0.516666666666667	0.261904761904762	0.529411764705882	0.395833333333333
PLA001	38	16	22	33	11	0.592592592592593	0.6	0.4	0.421052631578947	0.402439024390244	0.572916666666667
PLA002	40	16	24	31	11	0.592592592592593	0.563636363636364	0.436363636363636	0.4	0.426829268292683	0.552083333333333
PLA003	37	15	22	34	11	0.576923076923077	0.607142857142857	0.392857142857143	0.405405405405405	0.402439024390244	0.572916666666667
PLA004	38	15	23	33	11	0.576923076923077	0.589285714285714	0.410714285714286	0.394736842105263	0.414634146341463	0.5625
PLA005	37	15	22	34	11	0.576923076923077	0.607142857142857	0.392857142857143	0.405405405405405	0.402439024390244	0.572916666666667
PLA006	38	15	23	33	11	0.576923076923077	0.589285714285714	0.410714285714286	0.394736842105263	0.414634146341463	0.5625
PLA007	38	16	22	33	11	0.592592592592593	0.6	0.4	0.421052631578947	0.402439024390244	0.572916666666667
PLA008	40	16	24	31	11	0.592592592592593	0.563636363636364	0.436363636363636	0.4	0.426829268292683	0.552083333333333
PLA009	49	16	33	22	11	0.592592592592593	0.4	0.6	0.326530612244898	0.536585365853659	0.458333333333333
PLA010	49	16	33	22	11	0.592592592592593	0.4	0.6	0.326530612244898	0.536585365853659	0.458333333333333
PLA011	54	17	37	17	10	0.62962962962963	0.314814814814815	0.685185185185185	0.314814814814815	0.580246913580247	0.4375
PLA012	54	17	37	17	10	0.62962962962963	0.314814814814815	0.685185185185185	0.314814814814815	0.580246913580247	0.4375
PLA013	57	16	41	14	10	0.615384615384615	0.254545454545455	0.745454545454545	0.280701754385965	0.62962962962963	0.40625
PLA014	59	16	43	12	10	0.615384615384615	0.218181818181818	0.781818181818182	0.271186440677966	0.654320987654321	0.395833333333333
PLA015	57	16	41	14	10	0.615384615384615	0.254545454545455	0.745454545454545	0.280701754385965	0.62962962962963	0.416666666666667
PLA016	56	16	40	15	10	0.615384615384615	0.272727272727273	0.727272727272727	0.285714285714286	0.617283950617284	0.427083333333333
PLA017	36	15	21	35	11	0.576923076923077	0.625	0.375	0.416666666666667	0.390243902439024	0.59375
PLA018	46	15	31	25	11	0.576923076923077	0.446428571428571	0.553571428571429	0.326086956521739	0.51219512195122	0.489583333333333
PLA019	51	16	35	20	10	0.615384615384615	0.363636363636364	0.636363636363636	0.313725490196078	0.555555555555556	0.479166666666667
PLA020	51	16	35	20	10	0.615384615384615	0.363636363636364	0.636363636363636	0.313725490196078	0.555555555555556	0.479166666666667
PLA021	51	16	35	20	10	0.615384615384615	0.363636363636364	0.636363636363636	0.313725490196078	0.555555555555556	0.46875
PLA022	51	16	35	20	10	0.615384615384615	0.363636363636364	0.636363636363636	0.313725490196078	0.555555555555556	0.46875
PLA023	57	15	42	14	11	0.576923076923077	0.25	0.75	0.263157894736842	0.646341463414634	0.385416666666667
PLA024	56	15	41	15	11	0.576923076923077	0.267857142857143	0.732142857142857	0.267857142857143	0.634146341463415	0.395833333333333
PLA025	59	16	43	12	11	0.592592592592593	0.218181818181818	0.781818181818182	0.271186440677966	0.658536585365854	0.375
PLA026	59	16	43	12	11	0.592592592592593	0.218181818181818	0.781818181818182	0.271186440677966	0.658536585365854	0.385416666666667
PLA027	53	16	37	18	11	0.592592592592593	0.327272727272727	0.672727272727273	0.30188679245283	0.585365853658537	0.4375
PLA028	47	15	32	24	12	0.555555555555556	0.428571428571429	0.571428571428571	0.319148936170213	0.530120481927711	0.46875
PLA029	37	14	23	34	12	0.538461538461538	0.596491228070175	0.403508771929825	0.378378378378378	0.421686746987952	0.5625
PLA030	37	15	22	34	11	0.576923076923077	0.607142857142857	0.392857142857143	0.405405405405405	0.402439024390244	0.572916666666667

Plasmodium yoelii yoelii YM

	Total	TP	FP	TN	FN	Sensitivity	Speficity	Recall	Precision	Error rate	AUC
PLA000	41	11	30	24	14	0.44	0.444444444444444	0.555555555555556	0.268292682926829	0.556962025316456	0.393115942028985
PLA001	35	17	18	30	10	0.62962962962963	0.625	0.375	0.485714285714286	0.373333333333333	0.596014492753623
PLA002	37	17	20	28	10	0.62962962962963	0.583333333333333	0.416666666666667	0.459459459459459	0.4	0.574275362318841
PLA003	34	16	18	31	10	0.615384615384615	0.63265306122449	0.36734693877551	0.470588235294118	0.373333333333333	0.596014492753623
PLA004	35	16	19	30	10	0.615384615384615	0.612244897959184	0.387755102040816	0.457142857142857	0.386666666666667	0.585144927536232
PLA005	34	16	18	31	10	0.615384615384615	0.63265306122449	0.36734693877551	0.470588235294118	0.373333333333333	0.596014492753623
PLA006	35	16	19	30	10	0.615384615384615	0.612244897959184	0.387755102040816	0.457142857142857	0.386666666666667	0.585144927536232
PLA007	35	17	18	30	10	0.62962962962963	0.625	0.375	0.485714285714286	0.373333333333333	0.596014492753623
PLA008	37	17	20	28	10	0.62962962962963	0.583333333333333	0.416666666666667	0.459459459459459	0.4	0.574275362318841
PLA009	44	17	27	21	10	0.62962962962963	0.4375	0.5625	0.386363636363636	0.493333333333333	0.498188405797101
PLA010	44	17	27	21	10	0.62962962962963	0.4375	0.5625	0.386363636363636	0.493333333333333	0.498188405797101
PLA011	49	18	31	16	9	0.666666666666667	0.340425531914894	0.659574468085106	0.36734693877551	0.540540540540541	0.47554347826087
PLA012	49	18	31	16	9	0.666666666666667	0.340425531914894	0.659574468085106	0.36734693877551	0.540540540540541	0.47554347826087
PLA013	52	17	35	13	9	0.653846153846154	0.270833333333333	0.729166666666667	0.326923076923077	0.594594594594595	0.442934782608696
PLA014	53	17	36	12	9	0.653846153846154	0.25	0.75	0.320754716981132	0.608108108108108	0.432065217391304
PLA015	51	17	34	14	9	0.653846153846154	0.291666666666667	0.708333333333333	0.333333333333333	0.581081081081081	0.453804347826087
PLA016	50	17	33	15	9	0.653846153846154	0.3125	0.6875	0.34	0.567567567567568	0.464673913043478
PLA017	32	16	16	33	10	0.615384615384615	0.673469387755102	0.326530612244898	0.5	0.346666666666667	0.617753623188406
PLA018	40	16	24	25	10	0.615384615384615	0.510204081632653	0.489795918367347	0.4	0.453333333333333	0.530797101449275
PLA019	45	17	28	20	9	0.653846153846154	0.416666666666667	0.583333333333333	0.377777777777778	0.5	0.508152173913043
PLA020	45	17	28	20	9	0.653846153846154	0.416666666666667	0.583333333333333	0.377777777777778	0.5	0.508152173913043
PLA021	45	17	28	20	9	0.653846153846154	0.416666666666667	0.583333333333333	0.377777777777778	0.5	0.508152173913043
PLA022	45	17	28	20	9	0.653846153846154	0.416666666666667	0.583333333333333	0.377777777777778	0.5	0.508152173913043
PLA023	51	16	35	14	10	0.615384615384615	0.285714285714286	0.714285714285714	0.313725490196078	0.6	0.422101449275362
PLA024	50	16	34	15	10	0.615384615384615	0.306122448979592	0.693877551020408	0.32	0.586666666666667	0.432971014492754
PLA025	54	17	37	11	10	0.62962962962963	0.229166666666667	0.770833333333333	0.314814814814815	0.626666666666667	0.40036231884058
PLA026	53	17	36	12	10	0.62962962962963	0.25	0.75	0.320754716981132	0.613333333333333	0.411231884057971
PLA027	46	17	29	19	10	0.62962962962963	0.395833333333333	0.604166666666667	0.369565217391304	0.52	0.476449275362319
PLA028	41	16	25	24	11	0.592592592592593	0.489795918367347	0.510204081632653	0.390243902439024	0.473684210526316	0.499094202898551
PLA029	32	15	17	33	11	0.576923076923077	0.66	0.34	0.46875	0.368421052631579	0.586050724637681
PLA030	33	16	17	32	10	0.615384615384615	0.653061224489796	0.346938775510204	0.484848484848485	0.36	0.606884057971015

Plasmodium vinckei vinckei strain vinckei

	Total	TP	FP	TN	FN	Sensitivity	Speficity	Recall	Precision	Error rate	AUC
PLA000	33	13	20	22	12	0.52	0.523809523809524	0.476190476190476	0.393939393939394	0.477611940298507	0.493243243243243
PLA001	27	16	11	28	10	0.615384615384615	0.717948717948718	0.282051282051282	0.592592592592593	0.323076923076923	0.643018018018018
PLA002	28	16	12	27	10	0.615384615384615	0.692307692307692	0.307692307692308	0.571428571428571	0.338461538461538	0.629504504504505
PLA003	26	15	11	29	11	0.576923076923077	0.725	0.275	0.576923076923077	0.333333333333333	0.622184684684685
PLA004	27	16	11	28	10	0.615384615384615	0.717948717948718	0.282051282051282	0.592592592592593	0.323076923076923	0.643018018018018
PLA005	26	15	11	29	11	0.576923076923077	0.725	0.275	0.576923076923077	0.333333333333333	0.622184684684685
PLA006	27	16	11	28	10	0.615384615384615	0.717948717948718	0.282051282051282	0.592592592592593	0.323076923076923	0.643018018018018
PLA007	27	16	11	28	10	0.615384615384615	0.717948717948718	0.282051282051282	0.592592592592593	0.323076923076923	0.643018018018018
PLA008	28	16	12	27	10	0.615384615384615	0.692307692307692	0.307692307692308	0.571428571428571	0.338461538461538	0.629504504504505
PLA009	33	16	17	22	10	0.615384615384615	0.564102564102564	0.435897435897436	0.484848484848485	0.415384615384615	0.561936936936937
PLA010	33	16	17	22	10	0.615384615384615	0.564102564102564	0.435897435897436	0.484848484848485	0.415384615384615	0.561936936936937
PLA011	39	16	23	16	10	0.615384615384615	0.41025641025641	0.58974358974359	0.41025641025641	0.507692307692308	0.480855855855856
PLA012	39	16	23	16	10	0.615384615384615	0.41025641025641	0.58974358974359	0.41025641025641	0.507692307692308	0.480855855855856
PLA013	42	16	26	13	9	0.64	0.333333333333333	0.666666666666667	0.380952380952381	0.546875	0.474662162162162
PLA014	43	16	27	12	9	0.64	0.307692307692308	0.692307692307692	0.372093023255814	0.5625	0.461148648648649
PLA015	42	16	26	13	9	0.64	0.333333333333333	0.666666666666667	0.380952380952381	0.546875	0.474662162162162
PLA016	41	16	25	14	9	0.64	0.358974358974359	0.641025641025641	0.390243902439024	0.53125	0.488175675675676
PLA017	27	16	11	28	9	0.64	0.717948717948718	0.282051282051282	0.592592592592593	0.3125	0.663851351351351
PLA018	31	16	15	24	9	0.64	0.615384615384615	0.384615384615385	0.516129032258065	0.375	0.609797297297297
PLA019	39	16	23	16	9	0.64	0.41025641025641	0.58974358974359	0.41025641025641	0.5	0.501689189189189
PLA020	39	16	23	16	9	0.64	0.41025641025641	0.58974358974359	0.41025641025641	0.5	0.501689189189189
PLA021	38	16	22	17	9	0.64	0.435897435897436	0.564102564102564	0.421052631578947	0.484375	0.515202702702703
PLA022	38	16	22	17	9	0.64	0.435897435897436	0.564102564102564	0.421052631578947	0.484375	0.515202702702703
PLA023	42	16	26	13	9	0.64	0.333333333333333	0.666666666666667	0.380952380952381	0.546875	0.474662162162162
PLA024	38	15	23	17	10	0.6	0.425	0.575	0.394736842105263	0.507692307692308	0.494369369369369
PLA025	42	16	26	13	10	0.615384615384615	0.333333333333333	0.666666666666667	0.380952380952381	0.553846153846154	0.453828828828829
PLA026	44	16	28	11	10	0.615384615384615	0.282051282051282	0.717948717948718	0.363636363636364	0.584615384615385	0.426801801801802
PLA027	39	16	23	16	10	0.615384615384615	0.41025641025641	0.58974358974359	0.41025641025641	0.507692307692308	0.480855855855856
PLA028	32	16	16	23	10	0.615384615384615	0.58974358974359	0.41025641025641	0.5	0.4	0.575450450450451
PLA029	29	16	13	26	10	0.615384615384615	0.666666666666667	0.333333333333333	0.551724137931034	0.353846153846154	0.615990990990991
PLA030	27	16	11	28	10	0.615384615384615	0.717948717948718	0.282051282051282	0.592592592592593	0.323076923076923	0.643018018018018

Plasmodium vinckei petteri strain CR

	Total	TP	FP	TN	FN	Sensitivity	Speficity	Recall	Precision	Error rate	AUC
PLA000	28	10	18	20	15	0.4	0.526315789473684	0.473684210526316	0.357142857142857	0.523809523809524	0.4375
PLA001	27	13	14	21	13	0.5	0.6	0.4	0.481481481481481	0.442622950819672	0.52328431372549
PLA002	28	13	15	20	13	0.5	0.571428571428571	0.428571428571429	0.464285714285714	0.459016393442623	0.508578431372549
PLA003	26	13	13	22	13	0.5	0.628571428571429	0.371428571428571	0.5	0.426229508196721	0.537990196078431
PLA004	28	13	15	20	13	0.5	0.571428571428571	0.428571428571429	0.464285714285714	0.459016393442623	0.508578431372549
PLA005	26	13	13	22	13	0.5	0.628571428571429	0.371428571428571	0.5	0.426229508196721	0.537990196078431
PLA006	28	13	15	20	13	0.5	0.571428571428571	0.428571428571429	0.464285714285714	0.459016393442623	0.508578431372549
PLA007	27	13	14	21	13	0.5	0.6	0.4	0.481481481481481	0.442622950819672	0.52328431372549
PLA008	28	13	15	20	13	0.5	0.571428571428571	0.428571428571429	0.464285714285714	0.459016393442623	0.508578431372549
PLA009	34	13	21	14	13	0.5	0.4	0.6	0.382352941176471	0.557377049180328	0.420343137254902
PLA010	34	13	21	14	13	0.5	0.4	0.6	0.382352941176471	0.557377049180328	0.420343137254902
PLA011	35	13	22	13	13	0.5	0.371428571428571	0.628571428571429	0.371428571428571	0.573770491803279	0.405637254901961
PLA012	35	13	22	13	13	0.5	0.371428571428571	0.628571428571429	0.371428571428571	0.573770491803279	0.405637254901961
PLA013	37	13	24	11	13	0.5	0.314285714285714	0.685714285714286	0.351351351351351	0.60655737704918	0.39093137254902
PLA014	37	13	24	11	13	0.5	0.314285714285714	0.685714285714286	0.351351351351351	0.60655737704918	0.39093137254902
PLA015	35	12	23	13	13	0.48	0.361111111111111	0.638888888888889	0.342857142857143	0.590163934426229	0.405637254901961
PLA016	34	12	22	14	13	0.48	0.388888888888889	0.611111111111111	0.352941176470588	0.573770491803279	0.420343137254902
PLA017	24	12	12	24	13	0.48	0.666666666666667	0.333333333333333	0.5	0.409836065573771	0.552696078431372
PLA018	28	12	16	20	13	0.48	0.555555555555556	0.444444444444444	0.428571428571429	0.475409836065574	0.493872549019608
PLA019	30	13	17	18	13	0.5	0.514285714285714	0.485714285714286	0.433333333333333	0.491803278688525	0.479166666666667
PLA020	30	13	17	18	13	0.5	0.514285714285714	0.485714285714286	0.433333333333333	0.491803278688525	0.479166666666667
PLA021	30	12	18	18	13	0.48	0.5	0.5	0.4	0.508196721311475	0.464460784313726
PLA022	30	12	18	18	13	0.48	0.5	0.5	0.4	0.508196721311475	0.464460784313726
PLA023	36	13	23	12	13	0.5	0.342857142857143	0.657142857142857	0.361111111111111	0.590163934426229	0.405637254901961
PLA024	33	12	21	15	14	0.461538461538462	0.416666666666667	0.583333333333333	0.363636363636364	0.564516129032258	0.41421568627451
PLA025	36	12	24	12	14	0.461538461538462	0.333333333333333	0.666666666666667	0.333333333333333	0.612903225806452	0.370098039215686
PLA026	38	12	26	10	14	0.461538461538462	0.277777777777778	0.722222222222222	0.315789473684211	0.645161290322581	0.340686274509804
PLA027	32	12	20	16	14	0.461538461538462	0.444444444444444	0.555555555555556	0.375	0.548387096774194	0.41421568627451
PLA028	30	12	18	18	14	0.461538461538462	0.5	0.5	0.4	0.516129032258065	0.443627450980392
PLA029	26	12	14	22	14	0.461538461538462	0.611111111111111	0.388888888888889	0.461538461538462	0.451612903225806	0.502450980392157
PLA030	27	13	14	21	13	0.5	0.6	0.4	0.481481481481481	0.442622950819672	0.52328431372549
