# Supplementary material for: FT-GPI, a highly sensitive and accurate predictor of GPI-anchored proteins, reveals the composition and evolution of the GPI proteome in Plasmodium species
Source: Malar J. 2023 Jan 25;22:27. doi: 10.1186/s12936-022-04430-0 (PMC9876418; doi:10.1186/s12936-022-04430-0)
Supplement: Supplementary file 10 — Additional file 10: Fig. S1. Size distribution of GPI-AP present in UNIPROT database. The 29,901 proteins recovered using a query (keyword:KW-0336) were distributed among 2,136 organisms. A. Distribution of the log10 of GPI-AP protein sizes. The graph was generated with ggplot. The colour gradients distinguish organisms within each vertical bar. B. Distribution density based on the hypothesis that the histogram in (A) is a mixture of two normal distributions. Functions were obtained using normalmixEM function from the mixtools library in R. Similar results were obtained using the Mclust function (not shown). Fig. S2. Detection of orthologs of the P. falciparum 3D7 reference set from Gilson, et al. [17] using FT-GPI with varying parameter combinations (miniature vertical axes) in 46 Haemosporida isolates. These 31 FT-GPI parameter sets are described in Suppl. Table S2. The horizontal bars depict the number of GPI-AP orthologs detected by each setting of FT-GPI in each isolate. Bars colours are arbitrary. Fig. S3. Evolution of the GPI-Proteome among Haemosprida using PLA001 FT-GPI parameters set. Gene encoding proteins with size over 210 aa were selected for this analysis. A. Heatmap representing the distribution of genes among species. The presence of orthologs and paralogs was established using OrthoMCL annotation. Only orthology groups presenting orthologs in more than four species were included in the present analysis. Presence of paralogs were detected for some genes and represented by the red colour scale. A GPI-AP was absent (white) either because it was not detected by PLA001 or the gene was not present in the genome. Some genes were represented by more than one OrthoMCL group. The discrepancy between synteny and orthology groups was due to rapid sequence evolution and shared homologies. Complete species name is given in given in B and suppl Table 3. Laverania-Pg differentiated P. gaboni and close species from the P. falciparum/P. reichenowi group of parasites [file 12936_2022_4430_MOESM10_ESM.docx]

***Additional Figures***


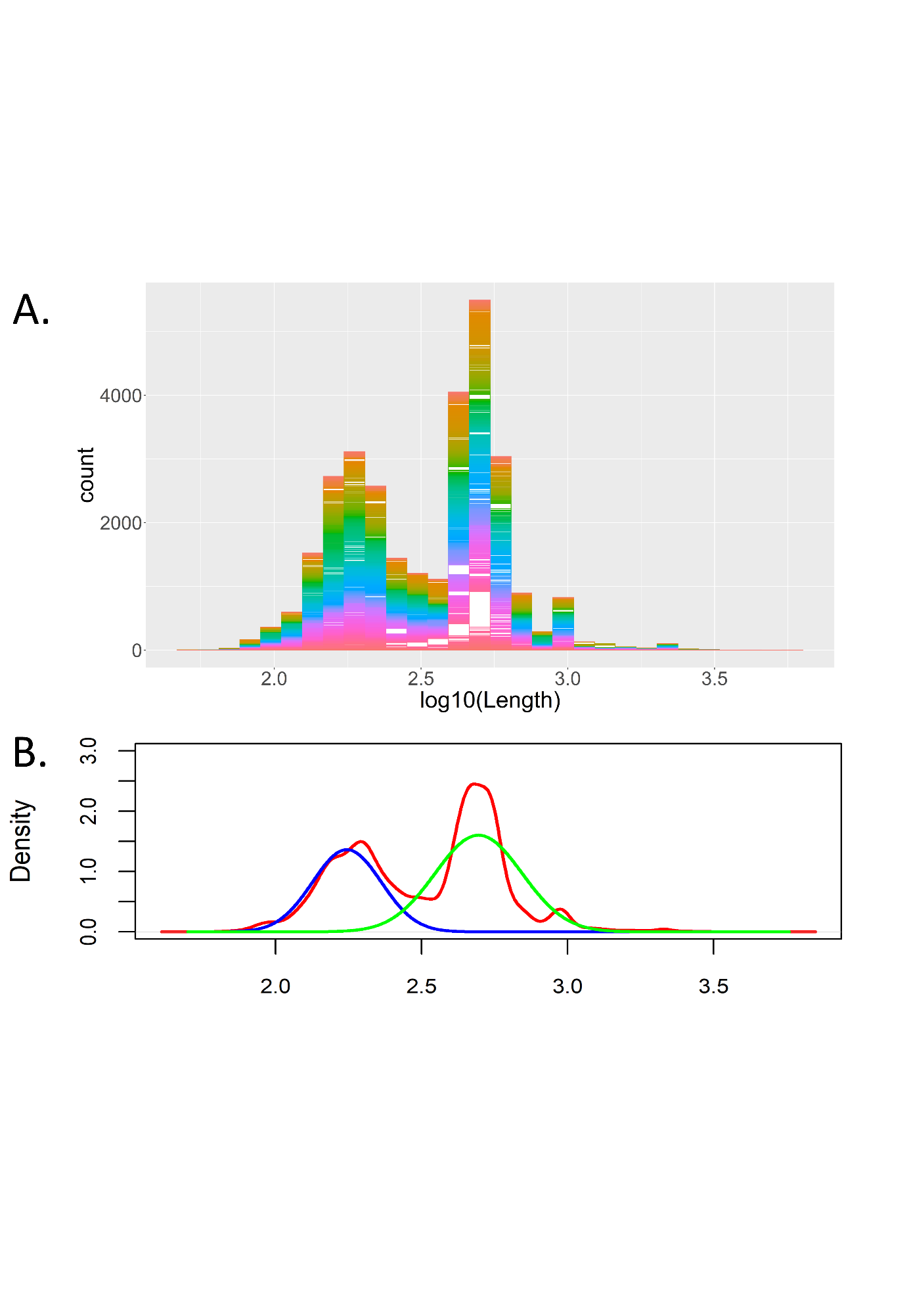


**Fig. S1. Size distribution of GPI-AP present in UNIPROT database.** The 29,901 proteins recovered using a query (keyword:KW-0336) were distributed among 2,136 organisms. **A.** Distribution of the log10 of GPI-AP protein sizes. The graph was generated with ggplot. The colour gradients distinguish organisms within each vertical bar. **B.** Distribution density based on the hypothesis that the histogram in (A) is a mixture of two normal distributions. Functions were obtained using normalmixEM function from the mixtools library in R. Similar results were obtained using the Mclust function (not shown).


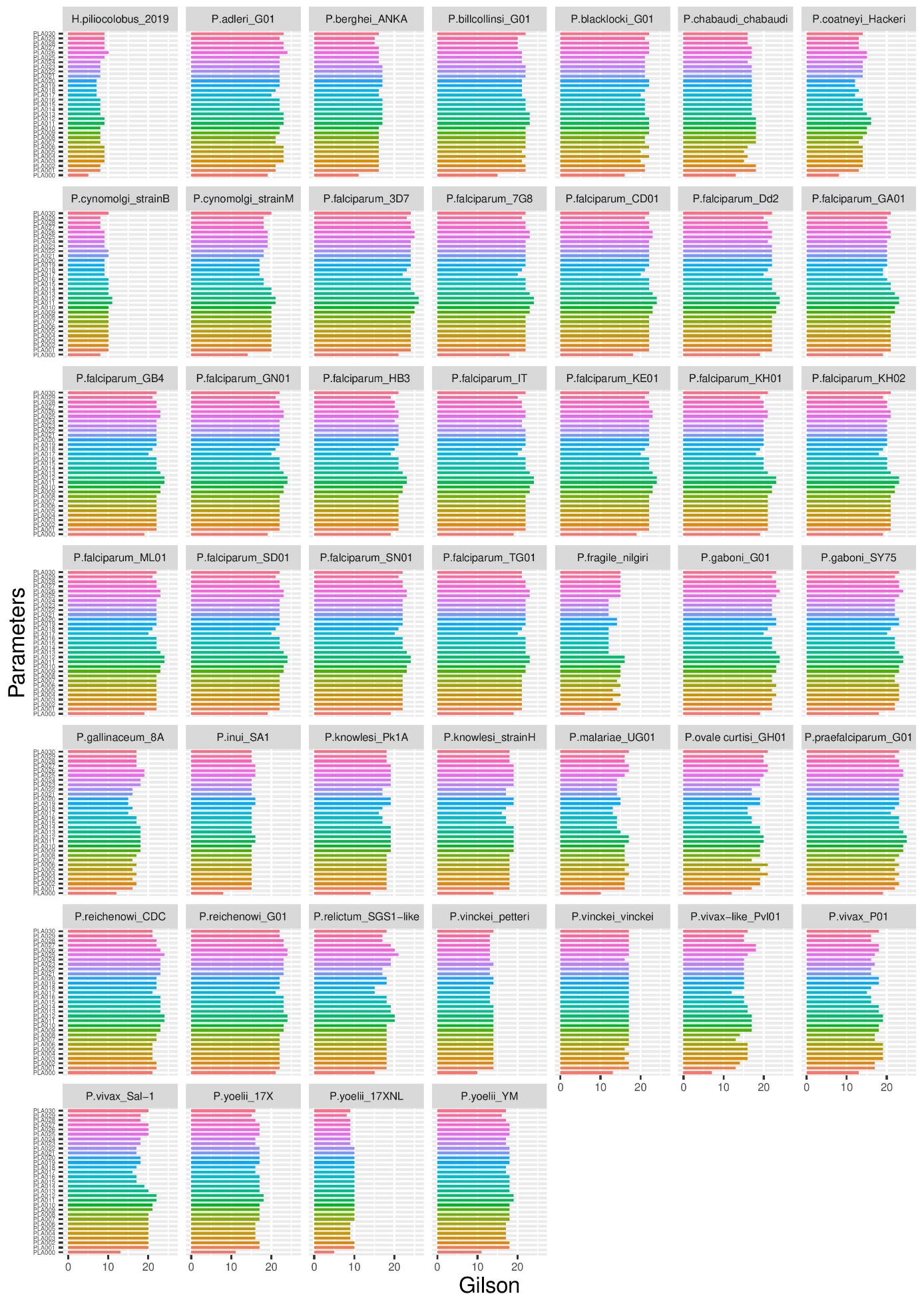


**Fig. S2. Detection of orthologs of the *P. falciparum* 3D7 reference set from Gilson, et al.** [17] **using FT-GPI with varying parameter combinations (miniature vertical axes) in 46 Haemosporida isolates.** These 31 FT-GPI parameter sets are described in Suppl. Table 2. The horizontal bars depict the number of GPI-AP orthologs detected by each setting of FT-GPI in each isolate. Bars colours are arbitrary.


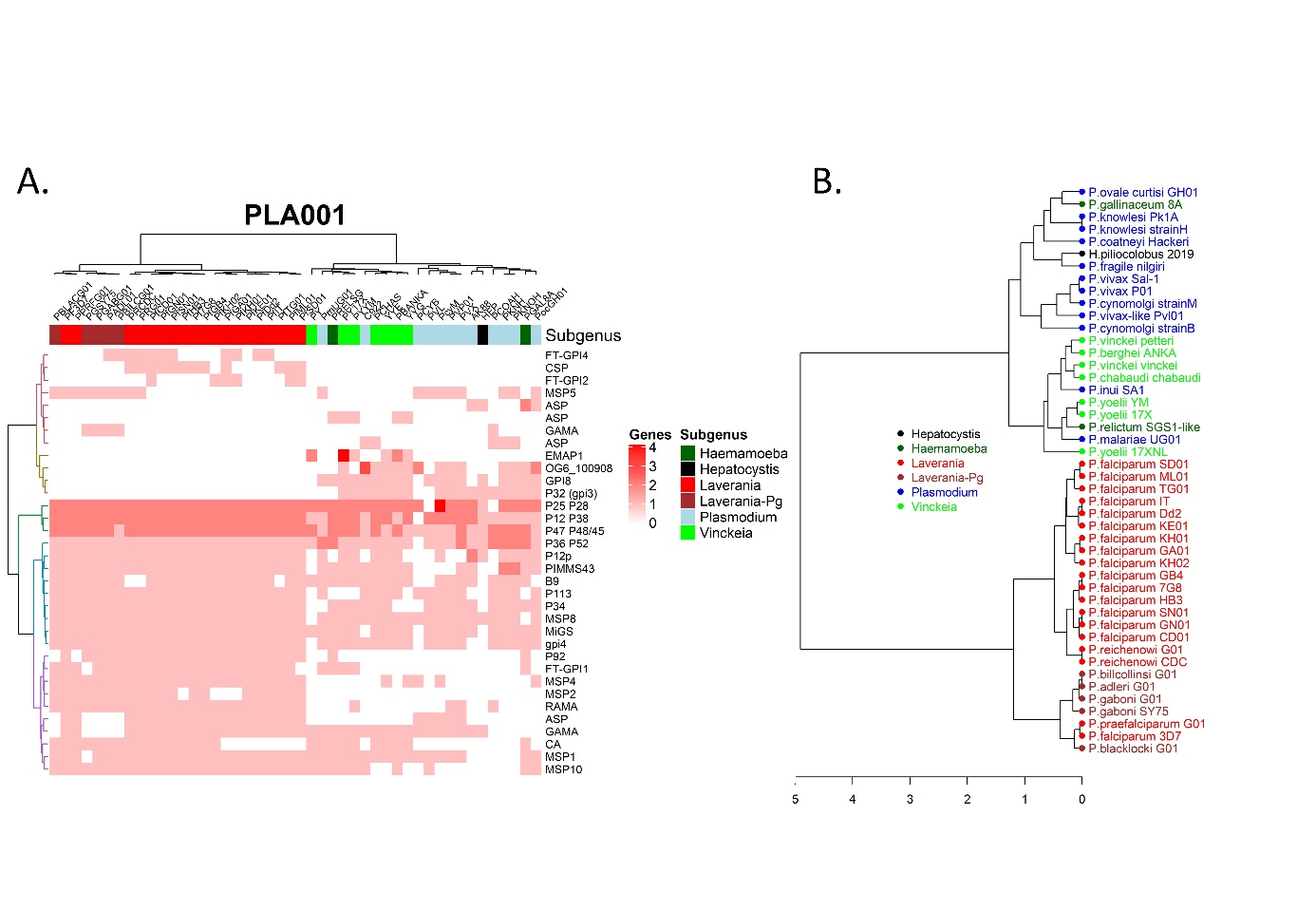


**Fig. S3. Evolution of the GPI-Proteome among Haemosprida using PLA001 FT-GPI parameters set.** Gene encoding proteins with size over 210 aa were selected for this analysis. **A. Heatmap representing the distribution of genes among species.** The presence of orthologs and paralogs was established using OrthoMCL annotation. Only orthology groups presenting orthologs in more than four species were included in the present analysis. Presence of paralogs were detected for some genes and represented by the red colour scale. A GPI-AP was absent (white) either because it was not detected by PLA001 or the gene was not present in the genome. Some genes were represented by more than one OrthoMCL group. The discrepancy between synteny and orthology groups was due to rapid sequence evolution and shared homologies. Complete species name is given in given in B and suppl Table 3. Laverania-Pg differentiated *P. gaboni* and close species from the *P. falciparum*/*P*. *reichenowi* group of parasites [68]. **B. Evolution of the GPI-proteome is related to speciation.** The genes were the same as in A. The number of paralogs was set to 1 to compute Jaccard distance. The Ward-2 method was used to build the tree.
